# Supplementary material for: Adaptive particle representation of fluorescence microscopy images
Source: Nat Commun. 2018 Dec 4;9:5160. doi: 10.1038/s41467-018-07390-9 (PMC6279843; doi:10.1038/s41467-018-07390-9)
Supplement: Supplementary file 1 — Supplementary Information [file 41467_2018_7390_MOESM1_ESM.pdf]

Supplementary material for: Adaptive Particle Representation of Fluorescence Microscopy Images

Cheeseman et al.

Supplementary Notes

1 Supplementary Note 1: Mathematical notation **3**

2 Supplementary Note 2: Main mathematical results **3**

3 Supplementary Note 3: General dimension Reconstruction Condition and Resolution Bound **6**

4 Supplementary Note 4: Particle Cells Definitions **7**

5 Supplementary Note 5: The Pulling Scheme **13**

6 Supplementary Note 6: Particle sampling **23**

7 Supplementary Note 7: Discrete sampling error and noise **25**

8 Supplementary Note 8: Comparison with optimal continuous Resolution Functions **34**

9 Supplementary Note 9: Generalized higher-order Reconstruction Conditions **39**

10 Supplementary Note 10: Image reconstruction methods **40**

11 Supplementary Note 11: 1D Validation **42**

12 Supplementary Note 12: 2D wavelet comparison **47**

13 Supplementary Note 13: 3D florescence image APR pipeline implementation **48**

14 Supplementary Note 14: Pipeline parameter summary **54**

15 Supplementary Note 15: 3D synthetic data **56**

16 Supplementary Note 16: Validation benchmark details **59**

17 Supplementary Note 17: Benchmark data **66**

18 Supplementary Note 18: Data structures **67**

19 Supplementary Note 19: APR formation timing **71**

20 Supplementary Note 20: File-storage of the APR **74**

21 Supplementary Note 21: APR particle graph **75**

22 Supplementary Note 22: Processing benchmark details **76**

Supplementary References **86**

A significant portion of these supplementary notes was originally presented in the doctoral thesis of Bevan L. Cheeseman [1] and is reproduced here as a service to the reader.

Supplementary Note 1: Mathematical notation

When dealing with a function, round brackets, for example for $f : \mathbb{R}^2 \to \mathbb{R}$, then $f(x, y)$ denotes the function f evaluated at (x, y). Later, we also use $f(\mathbf{y})$ and multi-index notation, where for the same example in 2D, $\mathbf{y} = (x, y)$ and $f(x, y) = f(\mathbf{y})$.

We consider a pixel image as the set generated from evaluating a function at a set of regularly spaced collocation points. For example, we define a 1D image of size N with spatial resolution h sampled from $[a, b]$ from some function $f : [a, b] \to \mathbb{R}$ as

$$f\{\bar{x}\} = \{f(x_i)|x_i \in \bar{x}\} \tag{1}$$

where $\bar{x} = \{x_i|x_i = a + h(i-1), i = 1, 2, .., N\}$ are the evenly spaced collocation points, and $h = (b-a)/(N-1)$. The notation holds not just for pixel images, but also for arbitrary vectors and samplings with $\bar{x} \in \mathbb{R}^N$. To avoid ambiguity with vector norms, we denote the cardinality of a set by $\#$, so in the 1D image example we have $\#\bar{x} = \#f\{\bar{x}\} = N$.

Pixel images in higher dimensions follow the convention as follows, with a 3D pixel image, being defined for $f : [a_x, b_x] \times [a_y, b_y] \times [a_z, b_z] \to \mathbb{R}$, being defined as $f\{(\bar{x}, \bar{y}, \bar{z})\} = \{f(x_i, y_i, z_i)|(x_i, y_i, z_i) \in \bar{x} \times \bar{y} \times \bar{z}\}$. Where the number of samples in each direction is N_x, N_y, N_z, with h_x, h_y, h_z, for the x, y, z directions respectively. Therefore, the total number of samples in the pixel image $N = \#f\{(\bar{x}, \bar{y}, \bar{z})\} = N_x N_y N_z$. When written as $f\{x, y, z\}$, where the arguments are not explicitly vectors, or defined constants, the expression can be interpreted as to hold for any of sampled collocation points.

Supplementary Note 2: Main mathematical results

The Adaptive Particle Representation (APR) takes a regularly sampled input function, such as pixel images, and resamples it as a set of particles $\mathcal{P}$ and a Resolution Function $R(y)$ defined for all locations y in the domain Ω. Where particles p are collocation points in space, x_p that 'carry' properties evaluated at that location, for example the function value $f_p = f(x_p)$ (or an estimate). In this way, then $\mathcal{P}$ is the set carrying all information for describing N_p particles, i.e. $\mathcal{P} = \{\{x_p\}_{p=1}^{N_p}, \{f_p\}_{p=1}^{N_p}\}$, being extended to include sets of other properties if required. The Resolution Function $R : \Omega \to \mathbb{R}$ defines a local isotropic neighborhood $\mathcal{N}$ at each point in the domain $\Omega \subset \mathbb{R}$ defining a subset of particles that can be used in the reconstruction of the function at that y. Therefore, $R(y)$ defines a isotropic spatial length scale at every point in the domain.

Formally, we consider a differentiable function $f : \Omega \to \mathbb{R}$, that is known at sampled location with fixed spacing, and is denoted as $f\{\bar{x}\}$ where $\#\bar{x} = N$. We represent the function for a given $\mathcal{P}$ and $R(y)$ in the following way

$$\hat{f}(y) = \sum_{x_p \in \mathcal{N}(y, R(y))} f(x_p)\xi_p(y) \tag{2}$$

where $\mathcal{N}(y, R(y)) = \{x \in \Omega : |x - y| \leq R(y)\}$ and $\xi_p(y) = \xi(y, x_p)$ are constants that satisfy

$$\sum_{x_p \in \mathcal{N}(y, R(y))} \xi_p(y) = 1$$

with $\xi_p(y) \geq 0$. (This positivity constraint can be relaxed, with a slight adjustment to the results, with addition of reconstruction dependent constant.) Where $x_p \in \mathcal{N}(y, R(y))$, means all particles in $\mathcal{P}$ that are in the neighborhood defined by $\mathcal{N}(y, R(y))$. The Resolution Function is set such that the reconstruction follows,

$$\|\frac{f - \hat{f}}{\sigma}\|_\infty \leq E \tag{3}$$

where $\|\mathbf{x}\|_\infty = \max_{x_i \in \bar{x}} x_i$, E is a user-specified relative error threshold and $\sigma(y)$ called the Local Intensity Scale ($\sigma : \Omega \to \mathbb{R}$, and is required to satisfy an additional 'smoothness constraint' given in 16). We call this bound the Reconstruction Condition, and this holds for any ξ_p satisfying the conditions above and any $\mathcal{P}$, where $\#(x_p \in \mathcal{N}(\mathbf{y}, R(\mathbf{y}))) > 0$ for all $y \in \Omega$ (That is, there is at least one support particle in the isotropic neighborhood set by $R(y)$).

The APR places two further restrictions on the problem defined above. First, we constrain the resolution function $R(y)$ to also satisfy

$$R(y) \leq \min_{x \in \mathcal{N}(y, R(y))} (L(x)) \tag{4}$$

where $L(y) = |\frac{E\sigma(y)}{\frac{\partial f}{\partial y}}|$ is called the Local Resolution Estimate and we assume we have access to $\frac{\partial f}{\partial y}$. We call the constraint 4 the Resolution Bound. If $R(y)$ satisfies the Resolution Bound, then it also satisfies the Reconstruction Condition (for special σ). We note, that when $\frac{\partial f}{\partial y} = 0$, and hence $L(y)$ is not defined, we can interpret this simply as the point not placing any constraint on the global resolution function at that point. Therefore, practically, divergent $L(y)$ does not present an issue. Second, we constrain $R(y)$ to be an Implied Resolution Function $R^*(y)$, that is, it is constructed out of piecewise constant blocks we call Particle Cells.

Result 1

Here we present a worst-case linear complexity in N algorithm, known as the Pulling Scheme, that can find the optimal $R^*(y)$ and particle set $\mathcal{P}$ that satisfy problems in the form of the Resolution Bound (4) for general $L(y)$. Where the optimal Implied Resolution Function is the R^* that satisfies

$$\arg\max_{R^* \in \mathcal{R}^*} \int_\Omega R^*(y) dy \tag{5}$$

where $\mathcal{R}^*$ is the set of all Implied Resolution Functions $R^*(y)$ that satisfy the Resolution Bound 4. The optimal $\mathcal{P}^*$ is then the particle set that satisfies, $\#(x_p \in \mathcal{N}(y, R(y))) > 0$, and $\#\mathcal{P} = \int_\Omega \frac{1}{R(y)} dy$.

Result 2

Given the local resolution function $\sigma(y)$ is sufficiently slowly varying (see 16), and $L(y) = |\frac{E\sigma(y)}{\frac{\partial f}{\partial y}}|$, then the reconstructions $\hat{f}$ formed using $R^*(y)$ and $\mathcal{P}$ will satisfy the Reconstruction Condition 3.

Result 3

The Implied Resolution Function $R^*(y)$ and Particle Cell set $\mathcal{P}$, can be completely described by a set of Particle Cells $\mathcal{V} = \{\{c_{i_p, l_p}\}_{p=1}^{N_p} | (i_p, l_p) \in \mathbb{Z}\}$ (i.e. it can be defined by N_p length sets of integers) and $\mathcal{P}^* = \{\{f_p\}_{p=1}^{N_p}\}$. The combination of these two sets $\{\mathcal{V}, \mathcal{P}^*\}$ we call the Adaptive Particle Representation (APR). (With the difference between $\mathcal{P}$ and $\mathcal{P}^*$ being the explicit storage of the particle locations in the former.)

1D Reconstruction Condition and Resolution Bound

In this sub-section, we describe and explain the derivation of the main results from main text in 1D for simplicity of notation and explaining ideas. First, we go through the derivation of the Resolution Bound, and how it relates to the Reconstruction Condition.

Let us continue with the problem as outlined above, and consider the function represented as

$$\hat{f}(y) = \sum_{x_p \in \mathcal{N}(y,R(y))} f_p \xi_p(y) \tag{6}$$

where we do not assume any particular distribution of particles $\mathcal{P}$, but assume there is at least one $p \in \mathcal{N}(\mathbf{y}, R(\mathbf{y}))$ for all y. We then consider the reconstruction error at each point $y \in \Omega$ as

$$\epsilon(y) = f(y) - \hat{f}(y), \tag{7}$$

which by assuming the function has a continuous derivative can express this by taking Taylor series expansions of f_p centered at y and using the integral form of the remainder [2] as,

$$\begin{aligned}\epsilon(y) &= f(y) - \sum_{x_p \in \mathcal{N}(y,R(y))} f_p \xi_p(y) \\ &= f(y) - \sum_{x_p \in \mathcal{N}(y,R(y))} \Bigg(f(y)\xi_p(y) + \\ & (y - x_p)\xi_p(y) \int_0^1 \frac{\partial}{\partial y} f(y + s(x_p - y))ds \Bigg)\end{aligned} \tag{8}$$

now by using that $\sum_{x_p \in \mathcal{N}(y,R(y)))} \xi_p(y) = 1$

$$\epsilon(y) = \sum_{x_p \in \mathcal{N}(y,R(y))} (y - x_p)\xi_p(y) \int_0^1 \frac{\partial}{\partial y} f(y + s(x_p - y))ds. \tag{9}$$

We can bound this exact expression of the error, using a uniform estimate, by bounding each integral using the maximum gradient over the interval and using the triangle inequality and the fact that by definition $|(y - x_p)| \le R(y)$ we get

$$|\epsilon(y)| \le \left(\sum_{x_p \in \mathcal{N}(y,R(y))} |\xi_p(y)| \right) R(y) \max_{x^* \in \mathcal{N}(y,R(y))} |\frac{\partial f(x_i)}{\partial y}| \tag{10}$$

and in now assuming also $\xi_p > 0$ (the procedure from here can be done without this assumption, however this leaves the sum of the coefficients in the resulting expressions) therefore $\left(\sum_{x_p \in \mathcal{N}(y,R(y))} |\xi_p(y)| \right) = 1$ so we get

$$|\epsilon(y)| \le R(y) \max_{x^* \in \mathcal{N}(y,R(y))} |\frac{\partial f(x^*)}{\partial y}|. \tag{11}$$

Now returning to the Reconstruction Condition, we can re-write the inifnity norm as a bound on each $y \in \Omega$ as

$$|\epsilon(y)| \le E\sigma(y). \tag{12}$$

So, using Supplementary Equation 11, Supplementary Equation 12 will be satisfied, if

$$R(y)\max_{x^*\in\mathcal{N}(y,R(y))}|\frac{\partial f(x^*)}{\partial y}|\leq E\sigma(y) \tag{13}$$

which we can formulation in terms of the Resolution Function as

$$R(y)\leq\frac{E\sigma(y)}{\max_{x^*\in\mathcal{N}(y,R(y))}|\frac{\partial f(x^*)}{\partial y}|}. \tag{14}$$

This we can then re-write as

$$R(y)\leq\sigma(y)\min_{x^*\in\mathcal{N}(y,R(y))}(g(x^*)) \tag{15}$$

where $g(x)=\frac{E}{|\frac{\partial f(x)}{\partial y}|}$ which we can see is almost the Resolution Bound, only the local intensity scale $\sigma(y)$ is outside the max.

Restriction on Local Intensity Scale

To get Supplementary Equation 15, into the form the Resolution Bound requires an assumption that

$$\sigma(y)\min_{x\in\mathcal{N}(y,R(y))}(g(x))=\min_{x\in\mathcal{N}(y,R(y))}(\sigma(y)g(x)) \tag{16}$$

this, therefore, provides a constraint for the information scale $\sigma(y)$ to ensure this approximation is valid. For this to approximately hold, $\sigma(y)$ must be sufficiently slowly varying. That is it must be approximately constant over $\mathcal{N}(\mathbf{y},R(\mathbf{y}))$. In general, this can not be guaranteed except in the case where $\sigma(y)=\sigma_0$ is a constant. However, in the resultspresented here indicate that the reconstruction condition still holds when $\sigma(y)$ is a smoothed local estimate of the range of $f(y)$. Further, the restriction is slightly relaxed for Implied Resolution Functions as discussed in Supplementary Note: 4.

Supplementary Note 3: General dimension Reconstruction Condition and Resolution Bound

Here we present derivation of the Resolution Bound in general dimension, it differs little from the one-dimensional case. We begin with the Reconstruction Condition state point wise as

$$|f(\mathbf{y})-\sum_{\mathbf{x}_p\in\mathcal{N}(\mathbf{y},R(\mathbf{y}))}f_p\xi_p(\mathbf{y})|\leq\frac{E}{\sigma(\mathbf{y})} \tag{17}$$

which holds must hold for all $\mathbf{y}\in\Omega$. Therefore again we proceed by considering the exact formulation of the error as

$$\epsilon(\mathbf{y})=f(\mathbf{y})-\sum_{\mathbf{x}_p\in\mathcal{N}(\mathbf{y},R(\mathbf{y}))}f_p\xi_p(\mathbf{y}) \tag{18}$$

now if we again assume function is C^1 can express by taking Taylor series expansions of f_p centered at $\mathbf{y}$ and using the integral form of the remainder for the Taylor series [2]

$$\epsilon(\mathbf{y})=\sum_{\mathbf{x}_p\in\mathcal{N}(\mathbf{y},R(\mathbf{y}))}\sum_{|\mathbf{k}|=1}(\mathbf{y}-\mathbf{x}_p)^{\mathbf{k}}\int_0^1\frac{\partial}{\partial\mathbf{x}^{\mathbf{k}}}f(\mathbf{y}+s(\mathbf{x}_p-\mathbf{y}))ds\xi_p(\mathbf{y}) \tag{19}$$

where $\mathbf{k}$ is using multi-index notation (See [2] for a brief description). In this case, it simply denotes summing over each spatial direction. Which we note is equivalent to the fundamental theorem of calculus and can be written as a path integral, and again using the triangle inequality

$$|\epsilon(\mathbf{y})| \leq \sum_{\mathbf{x}_p \in \mathcal{N}(\mathbf{y}, R(\mathbf{y}))} |\xi_p(\mathbf{y})||(\mathbf{y} - \mathbf{x}_p)| \int_0^1 |\nabla f(\mathbf{y} + s(\mathbf{x}_p - \mathbf{y}))| ds \tag{20}$$

where $\nabla f(\mathbf{x})$, represents the gradient operator. Now again given that $|\mathbf{y} - \mathbf{x}_p| \leq R(\mathbf{y})$ then

$$|\epsilon(\mathbf{y})| \leq \left(\sum_{\mathbf{x}_p \in \mathcal{N}(\mathbf{y}, R(\mathbf{y}))} |\xi_p(\mathbf{y})| \right) R(\mathbf{y}) \max_{\mathbf{x} \in \mathcal{N}(\mathbf{y}, R(\mathbf{y}))} (|\nabla f(\mathbf{y})|) \tag{21}$$

and so then again given we assume $\xi_p(\mathbf{y}) > 0$ then using this bound, Supplementary Equation 17 will hold if

$$R(\mathbf{y}) \leq \min_{\mathbf{x} \in \mathcal{N}(\mathbf{y}, R(\mathbf{y}))} \left(\frac{E\sigma(\mathbf{y})}{|\nabla f(\mathbf{x})|} \right), \tag{22}$$

which then assuming sufficient smoothness of $\sigma(\mathbf{y})$, such that the approximation

$$\max_{\mathbf{x} \in \mathcal{N}(\mathbf{y}, R(\mathbf{y}))} \left(\frac{|\nabla f(\mathbf{x})|}{\sigma(\mathbf{y})} \right) = \max_{\mathbf{x} \in \mathcal{N}(\mathbf{y}, R(\mathbf{y}))} \left(\frac{|\nabla f(\mathbf{x})|}{\sigma(\mathbf{x})} \right) \tag{23}$$

holds then

$$R(\mathbf{y}) \leq \min_{\mathbf{x} \in \mathcal{N}(\mathbf{y}, R(\mathbf{y}))} \left(\frac{E\sigma(\mathbf{x})}{|\nabla f(\mathbf{x})|} \right). \tag{24}$$

which is of the required form

$$R(\mathbf{y}) \leq \min_{\mathbf{x} \in \mathcal{N}(\mathbf{y}, R(\mathbf{y}))} (L(\mathbf{x})). \tag{25}$$

where the local resolution estimate is $L(\mathbf{y}) = \frac{E\sigma(\mathbf{y})}{|\nabla f(\mathbf{y})|}$.

Supplementary Note 4: Particle Cells Definitions

In this section, we introduce the general dimension treatment of Particle Cells. We begin with several definitions that will be useful.

For our given domain $\Omega \subset \mathbb{R}^n$, with maximum side length Ω_0. We begin by extending the Ω to a square domain $\Omega^* \in \mathbb{R}^d$, with edge length Ω_0, such that $\Omega \subseteq \Omega^*$. Next we introduce Particle Cells $\mathcal{C}$ that form a partition of the extended solution domain Ω^* and $\mathcal{R}$ the range of the possible resolution functions, $\mathcal{R} = \{R : \Omega \to \mathbb{R}^+\}$. Formally we enumerate the set $\mathcal{C}$, as

$$\mathcal{C} = \{c_{\mathbf{i},l}, \forall(\mathbf{i}, l) : l \in \mathbb{N}, i_k = 0, .., 2^l - 1\} \tag{26}$$

where $\mathbf{i} = i_1, .., i_n$ is multi-index notation for the spatial indices in each direction, and l indicates the level of the Particle Cell resolution. These Particle Cells form a partition using divisions of powers of 2, as follows,

$$\gamma(c_{\mathbf{i},l}) = [\frac{\Omega_0}{2^l}, \frac{\Omega_0}{2^{l+1}}) \times \prod_{\mathbf{i}} [i_k \frac{\Omega_0}{2^l}, (i_k + 1) \frac{\Omega_0}{2^l}) \tag{27}$$

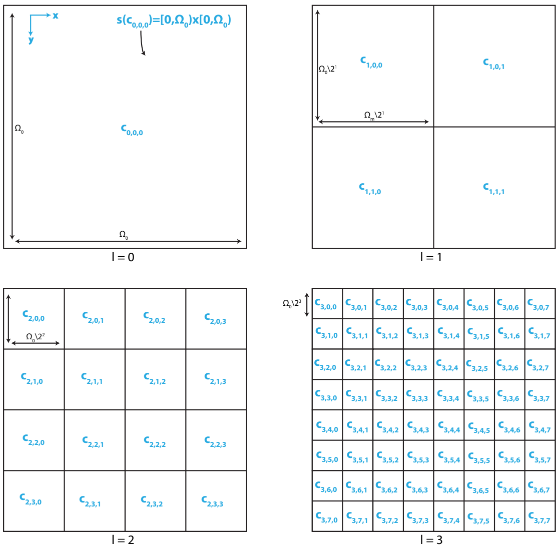

Supplementary Figure 1: Four levels l of $\mathcal{C}$ showing how Particle Cells in 2D $c_{\mathbf{i},l}$ partition the domain Ω on levels $l = \{0, 1, 2, 3\}$. Each square in the figure represents the spatial domain $s(c_{\mathbf{i},l})$ of a given Particle Cell $c_{\mathbf{i},l}$.

where the product is over all spatial indices and therefore,

$$\bigcup_{c_{\mathbf{i},l} \in \mathcal{C}} \gamma(c_{\mathbf{i},l}) = \Omega^* \times \mathcal{R}. \tag{28}$$

Each Particle Cell forms regular elements, rectangles in 1D, a half-cubes in 2D, and half-hypercubes in 3D. The 1D example is given in Figure 2. This partitioning is similar to those often used in quad and octree data structures, and as used in adaptive particle cell lists [3].

We define further properties of Particle Cells, reflecting their spatial domain, and resolution domain separately. The spatial domain of a Particle Cell is defined as,

$$s(c_{\mathbf{i},l}) = \prod_{\mathbf{i}} [i_k \frac{\Omega_0}{2^l}, (i_k + 1)\frac{\Omega_0}{2^l}) \tag{29}$$

Supplementary Figure 1 shows an example of $s(c_{\mathbf{i},l})$ of different Particle Cells for a range of l in 2D. The spatial domain of a Particle Cell $s(c_{\mathbf{i},l})$ is the area of the domain Ω^* of which it partitions. Effectively forming dyadic cubes of the domain Ω [4]. The resolution domain of a Particle Cell is defined as

$$r(c_{\mathbf{i},l}) = [\frac{\Omega_0}{2^l}, \frac{\Omega_0}{2^{l+1}}). \tag{30}$$

Further, we define $l(c_{\mathbf{i},l}) = l$, gives the level of $c_{\mathbf{i},l}$ and $\mathbf{i}(c_{\mathbf{i},l}) = \mathbf{i}$ can be used to give the spatial coordinate of $c_{\mathbf{i},l}$. Now given these definitions we can now define relationships between the Particle Cells considering them as constructing a tree structure as shown in Figure 2. We define the set of descendants of a particle cell $c_{\mathbf{i},l}$ as

$$\mathcal{D}(c_{\mathbf{i},l}) = \{c^d_{\mathbf{i},l} \in \mathcal{C} : s(c^d_{\mathbf{i},l}) \subset s(c_{\mathbf{i},l})\}, \tag{31}$$

which is the set of all Particle Cells who's spatial domain overlaps with $c_{\mathbf{i},l}$ but have a smaller resolution than $r(c_{\mathbf{i},l})$. The first set of descendants, called children, are shown for a cell in green in Figure 2F. Formally, children of $c_{\mathbf{i},l}$ are those $c^c_{\mathbf{i},l} \in D(c_{\mathbf{i},l})$ such that $l(c^c_{\mathbf{i},l}) = l(c_{\mathbf{i},l}) - 1$. We also then denote the parent of $c_{\mathbf{i},l}$, as $c_{\mathbf{i}/2,l-1}$, where $c_{\mathbf{i},l}$ is simply then the child of $c_{\mathbf{i}/2,l-1}$.

We also define the set of neighbors of a Particle Cell $c_{\mathbf{i},l}$, by first defining the interaction Particle Set

$$\mathcal{I}(c_{\mathbf{i},l}) = \{c^n_{\mathbf{i},l} \in \mathcal{C} : \exists \mathbf{x} \in s(c^n_{\mathbf{i},l}), \mathbf{y} \in s(c_{\mathbf{i},l}) : \mathbf{x} \in \mathcal{N}(\mathbf{y}, R(\mathbf{y}))\} \tag{32}$$

which is the set of all Particle Cells $c^n_{\mathbf{i},l}$ for which there is exists a $\mathbf{x}$ in its spatial domain and also a $\mathbf{y}$ in the spatial domain of $c_{\mathbf{i},l}$ such that they could interact, i.e. $\mathbf{x} \in \mathcal{N}(\mathbf{y}, R(\mathbf{y}))$. Then using the interaction Particle Cell set, we define the neighbor Particle Cell set as

$$\mathcal{B}(c_{\mathbf{i},l}) = \{c^n_{\mathbf{i},l} \in \mathcal{I}(c_{\mathbf{i},l}) : \nexists c^{n'}_{\mathbf{i},l} \in \mathcal{I} : s(c^n_{\mathbf{i},l}) \subset s(c^{n'}_{\mathbf{i},l})\} \tag{33}$$

which is the set of all neighboring Particle Cells of highest level that $c_{\mathbf{i},l}$ can interact with (including $c_{\mathbf{i},l}$). This definition and the theorems proven below hold across general definitions of the interaction neighborhood $\mathcal{N}(\mathbf{y}, R(\mathbf{y}))$. For simplicity of explanation, here we present examples with the isotropic interaction neighbourhood $\mathcal{N}(\mathbf{y}, R(\mathbf{y})) = \{\mathbf{x} \in \Omega : |\mathbf{x} - \mathbf{y}| \leq R(\mathbf{y})\}$, as introduced earlier. For the isotropic interaction neighborhood, the neighbor Particle Cell set is simply the neighboring Particle Cells of $c_{\mathbf{i},l}$ on the same level. This is illustrated in Figure 2F with a 1D example of a neighbor Particle Cell set $\mathcal{B}(c_{\mathbf{i},l})$ in blue.

Using these we define a set $\mathcal{ND} \in \mathcal{C}$ that contains all descendants of a particular Particle Cell $c_{\mathbf{i},l}$ and its neighbors as

$$\mathcal{ND}(c_{\mathbf{i},l}) = \bigcup_{c^n_{\mathbf{i},l} \in \mathcal{B}(c^v_{\mathbf{i},l})} \bigcup_{c^d_{\mathbf{i},l} \in \mathcal{D}(c^c_{\mathbf{i},l})} c^d_{\mathbf{i},l}. \tag{34}$$

Then any Particle Cell set $\mathcal{V} \subset \mathcal{C}$ forms a partition of the spatial domain Ω^* iff,

$$\bigcup_{c^v_{\mathbf{i},l} \in \mathcal{V}} s(c^v_{\mathbf{i},l}) = \Omega^*. \tag{35}$$

Then we can also define the set of Particle Cell sets $\mathcal{V}$ that form a spatial partition as

$$\mathcal{S} = \{\mathcal{V} : \mathcal{V} \subset \mathcal{C}, \bigcup_{c^v_{\mathbf{i},l} \in \mathcal{V}} s(c^v_{\mathbf{i},l}) = \Omega^*\}. \tag{36}$$

Lastly, we formally introduce an additional property of a Particle Cell called type, $t(c_{\mathbf{i},l})$, discussed in the previous section for Particle Cells when compared to a Particle Cell set $\mathcal{T}$ in the following way

$$t(c_{\mathbf{i},l}, \mathcal{T}) = \begin{cases} 1, & c_{\mathbf{i},l} \in \mathcal{T} \\ 2, & c_{\mathbf{i},l} \notin \mathcal{T} \text{ and } \exists c^n_{\mathbf{i},l} \in \mathcal{B}(c_{\mathbf{i},l}) : c^n_{\mathbf{i},l} \in \mathcal{T} \\ 3, & otherwise \end{cases}$$

where we name the three different Particle Cell types as seed, boundary, and filler respectively.

Implied Resolution Function

Now we define the Implied Resolution Function for a set of Particle Cells $\mathcal{V}$ that forms a spatial partition. We begin by now defining a characteristic function in general dimension as

$$\phi(\mathbf{y}, c_{\mathbf{i},l}) = \begin{cases} 1 & \mathbf{y} \in s(c_{\mathbf{i},l}) \\ 0 & otherwise \end{cases} \tag{37}$$

then the Implied Resolution Function $R^*(\mathbf{y})$ for a set of Particle Cells $\mathcal{V}$ that forms a partition of the spatial domain is

$$R^*(\mathbf{y}, \mathcal{V}) = \sum_{c_{i,l} \in \mathcal{V}} \phi(\mathbf{y}, c_{\mathbf{i},l}) \frac{\Omega_0}{2^l} \tag{38}$$

where we often drop the dependence on $\mathcal{V}$ below, unless required. One can interpret this Resolution Function as being built out of cube blocks of length $\frac{\Omega_0}{2^l}$, as shown in Figure 2D for 1D. In 1D the blocks are squares, 2D cubes, and 3D hypercubes. (Note: this is different from how Particle Cells are used to partition the resolution domain)

Local Particle Cell set

Given these definitions, we can now represent the Local Resolution Estimate $L(\mathbf{y})$ as Particle Cells. We assume that we have the following inequality to satisfy

$$R(\mathbf{y}) \leq \min_{\mathbf{x} \in \mathcal{N}(\mathbf{y}, R(\mathbf{y}))} L(\mathbf{x}). \tag{39}$$

We introduce the general dimension Local Particle Cell (LPC) set $\mathcal{L} \subseteq \mathcal{C}$ that has members such that

$$\mathcal{L} = \{c_{\mathbf{i},l} \in \mathcal{C} \Big| \exists\, (L(\mathbf{y}), \mathbf{y}) : \mathbf{y} \in s(c_{\mathbf{i},l}), L(\mathbf{y}) \in r(c_{\mathbf{i}/2,l-1})\}, \tag{40}$$

where $c_{\mathbf{i}/2,l-1}$ indicates the parent of $c_{\mathbf{i},l}$. In words, this takes the Local Resolution Estimate $L(\mathbf{y})$ and finds those Particle Cells $c_{\mathbf{i},l}$ whose parents intersect with $L(y)$ at locations inside the spatial domain $s(c_{\mathbf{i},l})$. An example was given is given for 1D in Figure 2G. We also define another set we call the natural Local Particle Cell (nLPC) set

$$\mathcal{L}_n = \{c_{\mathbf{i},l} \in \mathcal{C} \Big| \exists\, (L(\mathbf{y}), \mathbf{y}) \in \gamma(c_{\mathbf{i},l})\}, \tag{41}$$

in words takes the Local Resolution Estimate $L(\mathbf{y})$ and finds those Particle Cells that the function intersects. The second definition comes in use slightly later for the equivalence optimization and is called 'natural' due to its simpler definition. In all except special cases, $\mathcal{L}$ does not form a partition of the spatial domain.

In practice it is useful to specify a minimum level l_{min} and maximum level l_{max}. In these cases there then is effectively a minimum $L_{min}(\mathbf{y}) = \frac{\Omega_0}{2^{l_{min}}}$ and maximum value $L_{max}(\mathbf{y}) = \frac{\Omega_0}{2^{l_{max}}}$. Where for both $\mathcal{L}$ and $\mathcal{L}_n$ this effectively truncates any values with l below l_{min} to l_{min} and above l_{max} to l_{max}. (See Supplementary Note: 13 for a description of implementation and constructing these sets).

Optimal Valid Particle Cell sets

Now we have a way to relate, a Particle Cell set to a Resolution Function, if now we re-formulate Supplementary Equation 39, in terms of this Implied Resolution Function we have

$$R^*(\mathbf{y}) \leq \min_{\mathbf{x} \in \mathcal{N}(\mathbf{y}, R(\mathbf{y}))} L(\mathbf{x}). \tag{42}$$

we can now use the Implied Resolution Function and present the following theorem:

Theorem 1. $\mathcal{V}$ will define an Implied Resolution Function $R^*(\mathbf{y})$ thats satisfies Supplementary Equation 42 for all $\mathbf{y} \in \Omega^*$, for a given $\mathcal{L}$, and called valid iff it forms a spatial partition and

1. $\forall c^v_{\mathbf{i},l} \in \mathcal{V}$ then $\{\mathcal{L} \cap \mathcal{N}\mathcal{D}(c^v_{\mathbf{i},l})\} = \emptyset$

In words, for all Particle Cells $c_{\mathbf{i},l}$ in $\mathcal{V}$, the set is valid, if and only if, there are no Particle Cells that are descendants of $c_{\mathbf{i},l}$ or its neighbors in $\mathcal{L}$.
Proof:
Given a valid Particle Cell set $\mathcal{V}$, we suppose there exists at least one combination of $\mathbf{y} \in \Omega^*$ and $\mathbf{y}^* \in \mathcal{N}(\mathbf{y}, R(\mathbf{y}))$, such that

$$L(\mathbf{y}^*) < R^*(\mathbf{y}) \tag{43}$$

is true and therefore condition Supplementary Equation 42 is violated. In addition, there must exist $c^v_{\mathbf{i},l} \in \mathcal{V}$ such that $\mathbf{y} \in s(c^v_{\mathbf{i},l})$. From Supplementary Equation 38, we have

$$R^*(\mathbf{y}) = \frac{\Omega^*}{2^{l(c^v_{\mathbf{i},l})}} \tag{44}$$

and therefore if $L(\mathbf{y}^*) < \frac{\Omega^*}{2^{l(c^c_{\mathbf{i},l})}}$ then there must exist some $c^*_{\mathbf{i},l} \in \mathcal{L}$, for which $l(c^*_{\mathbf{i},l}) < l(c^v_{\mathbf{i},l})$ and $\mathbf{y}^* \in s(c^*_{\mathbf{i},l})$. Now since $\mathbf{y}^* \in \mathcal{N}(\mathbf{y}, R(\mathbf{y}))$ and $l(c^*_{\mathbf{i},l}) < l(c^v_{\mathbf{i},l})$ it implies that

$$c^*_{\mathbf{i},l} \in \mathcal{N}\mathcal{D}(c^v_{\mathbf{i},l}) \tag{45}$$

and therefore

$$\exists c^a_{\mathbf{i},l} \in \mathcal{V} : \{\mathcal{L} \cap \mathcal{N}\mathcal{D}(c^a_{\mathbf{i},l})\} \neq \emptyset \tag{46}$$

and proves Theorem. 1 by contradiction. □
Now we consider conditions on $\mathcal{V}$ that would define it as optimal. Consider $\mathbf{V}$ to be the set of all Particle Cell sets $\mathcal{V}$ that satisfy Theorem 1 and are valid. Then a Particle Cell set $\mathcal{V}$ will be optimal if it satisfies

$$\underset{\mathcal{V}^* \in \mathbb{V}}{\arg\max} \int_{\Omega^*} R^*(\mathbf{y}, \mathcal{V}^*) d\Omega^*. \tag{47}$$

which is equivalent to finding the largest everywhere $R^*(y)$ that satisfies Supplementary Equation 42. Which is equivalent to

$$\underset{R^* \in \mathcal{R}^*}{\arg\max} \int_{\Omega^*} R^*(\mathbf{y}) d\Omega^*. \tag{48}$$

where $\mathcal{R}^*$ is the set of all Implied Resolution Functions defined as Supplementary Equation 38 ($R^* : \Omega^* \to \mathbb{R}^+$) that satisfy Supplementary Equation 42. We can now state the following theorem for satisfying Supplementary Equation 47,

Theorem 2. Given $\mathcal{V} \subset \mathcal{C}$, that is valid, $\mathcal{V}$ will satisfy Supplementary Equation 47 and be optimal, iff, there does not exist a $\mathcal{W} \subset \mathcal{C}$ where $\mathcal{W} \neq \mathcal{V}$ and is valid for $\mathcal{L}$ and where $\mathcal{V}$ can be formed from the elements of $\mathcal{W}$ and its descendants. Formally, $\mathcal{V}$ is optimal, if there does not exist any valid $\mathcal{W}$ such that for any $c^w_{\mathbf{i},l}$ or $c^v_{\mathbf{i},l}$ the following holds

$$\left(c^w_{\mathbf{i},l} \in \mathcal{W}, c^v_{\mathbf{i},l} \in \mathcal{V}\right) : c^v_{\mathbf{i},l} \in D(c^w_{\mathbf{i},l}). \tag{49}$$

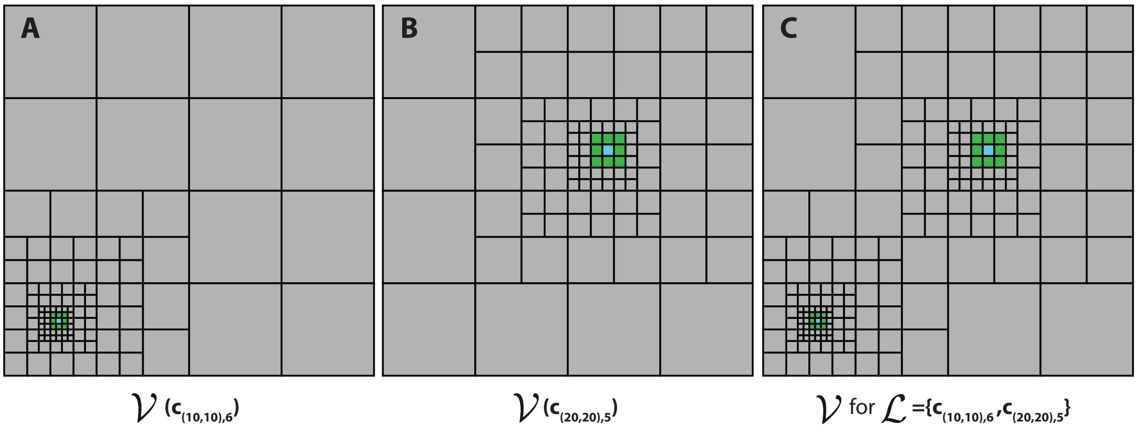

Supplementary Figure 2: Example of the optimal particle cell set $\mathcal{V}$ (**C**) for $\mathcal{L} = \{c_{(10,10),6}, c_{(20,20),5}\}$ in 2D, and the individual optimal solutions $\mathcal{V}^*(c_{(10,10),6})$ (**A**) and $\mathcal{V}^*(c_{(20,20),5})$ (**B**) that can be used to combined using the separability property to construct $\mathcal{V}$. The particle cells are colored in the following way, a particle cell is blue if its type is a seed if it is in the local particle cell set, $c_{\mathbf{i},l} \in \mathcal{L}$, a cell is green if it is of type boundary and therefore has a neighbor that is in the local particle cell set and is grey if it as of type filler.

In words, $\mathcal{V}$, is optimal, if there does not exist another arrangement of Particle Cells that form a spatial partition and is valid while having a larger resolution anywhere in the domain.
Proof:
Lets consider two Particle Cell sets $\mathcal{V}$ and $\mathcal{W}$, where both are valid with respect to $\mathcal{L}$, and $\mathcal{W} \neq \mathcal{V}$, and $\mathcal{V}$ is optimal. Now we suppose that, Supplementary Equation 47 is violated, that is

$$\int_{\Omega^*} R^*(\mathbf{y}, \mathcal{V}) d\Omega^* < \int_{\Omega^*} R^*(\mathbf{y}, \mathcal{W}) d\Omega^*, \tag{50}$$

that we can re-write as

$$\int_{\Omega^*} \sum_{c_{i,l} \in \mathcal{V}} \phi(\mathbf{y}, c_{\mathbf{i},l}) \frac{\Omega_0}{2^l} d\Omega^* < \int_{\Omega^*} \sum_{c_{i,l} \in \mathcal{W}} \phi(\mathbf{y}, c_{\mathbf{i},l}) \frac{\Omega_0}{2^l} d\Omega^*. \tag{51}$$

Given the above inequality to hold, there must exist $\mathbf{y} \in \Omega^*$ where the following holds for some $c^v_{\mathbf{i},l} \in \mathcal{V}$ and $c^w_{\mathbf{i},l} \in \mathcal{W}$,

$$0 < \phi(\mathbf{y}, c^v_{\mathbf{i},l}) \frac{\Omega_0}{2^{l(c^v_{\mathbf{i},l})}} < \phi(\mathbf{y}, c^w_{\mathbf{i},l}) \frac{\Omega_0}{2^{l(c^w_{\mathbf{i},l})}} \tag{52}$$

which implies that $l(c^w_{\mathbf{i},l}) < l(c^v_{\mathbf{i},l})$ and further that $s(c^v_{\mathbf{i},l}) \subset s(c^w_{\mathbf{i},l})$ and hence

$$c^v_{\mathbf{i},l} \in D(c^w_{\mathbf{i},l}), \tag{53}$$

which violates Theorem 2, and thus concludes the proof through contradiction $\square$.

Therefore a Particle Cell set $\mathcal{V}$ is an Optimal Valid Particle Cell (OVPC) set if it satisfies both Theorem 1 and Theorem 2.

Particle Cells and smoothness of the Local Intensity Scale

In practice, the use of an Implied Resolution Function relaxes the smoothness assumption on the Local Intensity Scale σ (Supplementary Note: 2). For a continuous Resolution Function an equality for the expression

$$\sigma(y) \min_{x \in \mathcal{N}(y,R(y))} (g(x)) \approx \min_{x \in \mathcal{N}(y,R(y))} (\sigma(y) g(x)) \tag{54}$$

would require a constant $\sigma(y)$ for general f. However, we note when using an Implied Resolution Function as in the APR we only will detect changes that would change the Particle Cell level l. Now for a given problem the Particle Cell level can be calculated as,

$$l = \lceil \log \left(\frac{|\Omega| \sigma(x)}{g(x)} \right) \rceil \tag{55}$$

with a -1 if the equivalence optimization is being used. We find that Supplementary Equation 54 is then equivalent to

$$\lceil \log \left(\frac{|\Omega|}{\sigma(x) g(x)} \right) \rceil = \lceil \log \left(\frac{|\Omega|}{\sigma(y) g(x)} \right) \rceil \tag{56}$$

for all $x \in \mathcal{N}(y, R(y))$ and $y \in \Omega$. This is a weaker bound then Supplementary Equation 54 potentially allowing non-constant $\sigma(y)$. Hence, the situation is not quite as restrictive as Supplementary Equation 54 implies when using an Implied Resolution Function.

Supplementary Note 5: The Pulling Scheme

Here we present the additional results based on the above that are used by the Pulling Scheme. These are the general definitions of the three properties from the previous chapter. We begin by defining

Definition 1. $\mathcal{V}^*(c_{\mathbf{i},l})$ is the optimal Particle Cell set for $\mathcal{L} = \{c_{\mathbf{i},l}\}$

That is the OVPC set for a LPC set with only one Particle Cell $c_{\mathbf{i},l}$.

Self-similarity and production of individual solutions

The first is an observation that the solution $\mathcal{V}^*(c_{\mathbf{i},l})$ is highly predictable and shows self-similarity regarding its relative local structure. This is shown for two different $c_{\mathbf{i},l}$ in 2D in Supplementary Note: 2, where the Particle Cells are colored by their type. The solutions are defined by a central seed Particle Cell, surrounded by a layer of boundary and then filler cells. The remainder of the domain is then filled with Particle Cells increasing by one level across neighbors, adding Particle Cells on the same level to maintain a spatial partition. An illustrative example in 1D is given in Supplementary Note: 3.

Separability

We present Lemma 1, that is the basis of the separability property used in Pulling Scheme.

Lemma 1. Given $\mathcal{V} \subset \mathcal{C}$ is optimal, with respect to $\mathcal{L}$, and let $\mathcal{V}^*(c_{\mathbf{i},l}) \subset \mathcal{C}$ be optimal for the local set $\mathcal{L}^*(c_{\mathbf{i},l}) = \{c_{\mathbf{i},l}\}$. Then,

$$\mathcal{V} = \text{minhull} \left(\bigcup_{c^l_{\mathbf{i},l} \in \mathcal{L}} \mathcal{V}^*(c^l_{\mathbf{i},l}) \right) \tag{57}$$

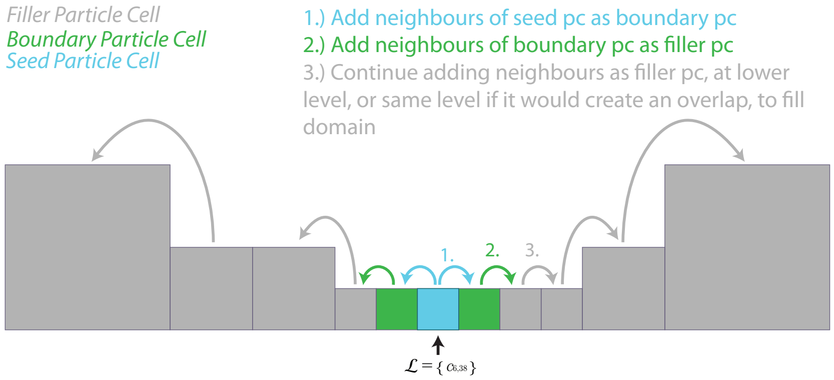

Supplementary Figure 3: Schematic how an OVPC set $\mathcal{V}$ can be generated when $\mathcal{L} = \{c_{i,l}\}$ has only one Particle Cell. We give Particle Cells an additional property called type, based on how the Particle Cell was added to the set $\mathcal{V}$. Particle cells that are in both $\mathcal{V}$ and $\mathcal{L}$ are of type seed. Particle cells that are neighbors to a seed cell are type boundary. All others, are of type filler. $\mathcal{V}$ is created by first adding $c_{i,l}$ (1.), and its neighbors (2.) on the same level and their neighbors (3.). The domain is then filled, allowing only one level change at once, and ensuring the resulting set forms a spatial partition.

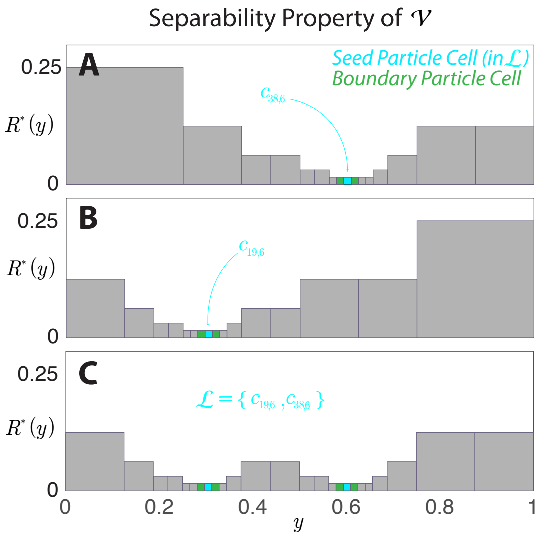

Supplementary Figure 4: The Pulling Scheme computes the local Optimal Valid Particle Cell (OVPC) set $\mathcal{V}$ (Particle Cells of all colours) for a given Local Particle Cell (LPC) set $\mathcal{L}$ (light blue Particle Cell). Due to the separability property (see Supplementary Note: 5), this can be done separately for each Local Particle Cell (**A** and **B**). The complete result for the combined set is then formed by taking the smallest Particle Cell at each location (**C**). Both axis are in spatial units.

where for $\mathcal{T} \subseteq \mathcal{C}$,

$$\text{minhull}(\mathcal{T}) = \{c^s_{\mathbf{i},l} \in \mathcal{T} : \{D(c^s_{\mathbf{i},l}) \cap \mathcal{T}\} = \emptyset\}. \tag{58}$$

In words, Lemma 1 states that the optimal solution $\mathcal{V}$, for a given LPC set $\mathcal{L}$, can be constructed by forming the valid and optimal set for each Particle Cell in $\mathcal{L}$ separately $\mathcal{V}^*(c_{\mathbf{i},l})$, and then forming a set with the Particle Cells $c_{\mathbf{i},l}$ at each point y with the smallest Implied Resolution Function $R^*(\mathbf{y}, \mathcal{V}^*(c_{\mathbf{i},l}))$ (highest level l). We call the above property, separability. Supplementary Note: 2 shows the property in 2D and Figure 3A in 1D. One can intuitively confirm that the configurations are optimal, by replacing any Particle Cell by its parent, and then checking if Theorem. 1 holds.

Proof:

Lets consider $\mathcal{V}$, which is optimal for $\mathcal{L}$, and $\hat{\mathcal{V}} = \text{minhull}\left(\bigcup_{c^l_{\mathbf{i},l} \in \mathcal{L}} \mathcal{V}^*(c^l_{\mathbf{i},l})\right)$. Now propose that, there exists some $c_{\hat{\mathbf{i}},l} \in \hat{\mathcal{V}}$ such that

$$\exists c^*_{\mathbf{i},l} \in \mathcal{L} : c^*_{\mathbf{i},l} \in \mathcal{ND}(c_{\hat{\mathbf{i}},l}) \tag{59}$$

and therefore $\hat{\mathcal{V}}$ would not be valid by Theorem 1. However, given that $\mathcal{V}^*(c^*_{\mathbf{i},l})$ is valid, it forms a spatial partition, and $c_{\hat{\mathbf{i}},l} \notin \mathcal{V}^*(c^*_{\mathbf{i},l})$, therefore

$$\exists c_{\bar{\mathbf{i}},l} \in \mathcal{V}^*(c^*_{\mathbf{i},l}) : c_{\bar{\mathbf{i}},l} \in D(c_{\hat{\mathbf{i}},l}) \tag{60}$$

and since $\mathcal{V}^*(c^*_{\mathbf{i},l}) \subset \bigcup_{c^l_{\mathbf{i},l} \in \mathcal{L}} \mathcal{V}^*(c^l_{\mathbf{i},l})$ then,

$$D(c_{\hat{\mathbf{i}},l}) \cap \bigcup_{c^l_{\mathbf{i},l} \in \mathcal{L}} \mathcal{V}^*(c^l_{\mathbf{i},l}) \supseteq c_{\bar{\mathbf{i}},l} \tag{61}$$

$$\neq \emptyset \tag{62}$$

therefore violating Lemma 1 as $c_{\hat{\mathbf{i}},l} \in \hat{\mathcal{V}}$. Therefore, by contradiction, given Lemma. 1 holds, $\hat{\mathcal{V}}$ will be valid.

Now, let us propose, that $\hat{\mathcal{V}}$ is not optimal, that is there exists some $\mathcal{W}$ such that

$$\int_{\Omega^*} R^*(\mathbf{y}, \hat{\mathcal{V}}) d\Omega^* < \int_{\Omega^*} R^*(\mathbf{y}, \mathcal{W}) d\Omega^*, \tag{63}$$

following the arguments for the proof of Theorem 2 above this implies there would exist some $c^w_{\mathbf{i},l} \in \mathcal{W}$ and some $c_{\hat{\mathbf{i}},l} \in \hat{\mathcal{V}}$ such that

$$c_{\hat{\mathbf{i}},l} \in D(c^w_{\mathbf{i},l}). \tag{64}$$

Given that $c_{\hat{\mathbf{i}},l} \in \hat{\mathcal{V}}$ there exists some $c_{\bar{\mathbf{i}},l} \in \mathcal{L}$ such that $c_{\hat{\mathbf{i}},l} \in \mathcal{V}^*(c_{\bar{\mathbf{i}},l})$. However, given $c^w_{\mathbf{i},l} \notin \mathcal{V}^*(c_{\bar{\mathbf{i}},l})$ and $\mathcal{V}^*(c_{\bar{\mathbf{i}},l})$ is optimal then,

$$\left(\mathcal{ND}(c^w_{\mathbf{i},l}) \cap \mathcal{V}^*(c_{\bar{\mathbf{i}},l})\right) \supseteq c_{\hat{\mathbf{i}},l} \neq \emptyset. \tag{65}$$

Since $c_{\bar{\mathbf{i}},l} \in \mathcal{L}$, then $\mathcal{W}$ cannot be valid. Implying that $\hat{\mathcal{V}}$ must be optimal for $\mathcal{L}$ and given the optimal solution is unique implies

$$\hat{\mathcal{V}} = \mathcal{V} \tag{66}$$

□.

Redundancy of Particle Cells

The third property relates to the redundancy of Particle Cells in $\mathcal{L}$ that have descendants in $\mathcal{L}$,

Lemma 2. Given any two Particle Cells $c_{\mathbf{i},l}$ and $c^p_{\mathbf{i},l}$, where $c_{\mathbf{i},l} \in D(c^p_{\mathbf{i},l})$ then

$$\text{minhull}(\{\mathcal{V}^*(c_{\mathbf{i},l}), \mathcal{V}^*(c^p_{\mathbf{i},l})\}) = \mathcal{V}^*(c_{\mathbf{i},l}). \tag{67}$$

In words, the optimal valid solution of Particle Cells for which one is the descendant of the other will be the individual valid solution of the descendant Particle Cell.

Proof:

Lets suppose that,

$$\exists c_{\hat{\mathbf{i}},l} \in \text{minhull}(\{\mathcal{V}^*(c_{\mathbf{i},l}), \mathcal{V}^*(c^p_{\mathbf{i},l})\}) : c_{\hat{\mathbf{i}},l} \notin \mathcal{V}^*(c_{\mathbf{i},l}) \tag{68}$$

and then

$$c_{\hat{\mathbf{i}},l} \in \mathcal{V}^*(c^p_{\mathbf{i},l}) \tag{69}$$

and Lemma 2 is violated. However, given the definition of the minhull operation, and the fact that $\mathcal{V}^*(c_{\mathbf{i},l})$ must form a spatial partition this implies that

$$\exists c^*_{\mathbf{i},l} \in \mathcal{V}^*(c_{\mathbf{i},l}) : c_{\hat{\mathbf{i}},l} \in D(c^*_{\mathbf{i},l}). \tag{70}$$

However, given that $\mathcal{V}^*(c^p_{\mathbf{i},l})$ is optimal by definition, then if $c^*_{\mathbf{i},l} \notin \mathcal{V}^*(c^p_{\mathbf{i},l})$, then by Theorem 2

$$c^p_{\mathbf{i},l} \in \mathcal{ND}(c^*_{\mathbf{i},l}) \tag{71}$$

and since $c_{\mathbf{i},l} \in D(c^p_{\mathbf{i},l})$ by construction then also

$$c_{\mathbf{i},l} \in \mathcal{ND}(c^*_{\mathbf{i},l}) \tag{72}$$

but this results in a contradiction, as then $\mathcal{V}^*(c_{\mathbf{i},l})$ would not be valid by Theorem. 1 $\square$.

Equivalence Optimization

The following results show that the OVPC set $\mathcal{V}$ can be obtained, by solving for a smaller set of Particle Cells with a maximum level one less than $\mathcal{L}$ and then directly replacing some Particle Cells in the produced sets with their child cells. First, let us define

Definition 2. Let $\mathcal{V}_n$ be the optimal Particle Cell set for the natural Local Particle Cell set $\mathcal{L}_n$ formed from $L(\mathbf{y})$ as in Supplementary Equation 41

Then we have the following result,

Lemma 3. Given $\mathcal{V}_n$ is optimal and valid for $\mathcal{L}_n$ then

$$\mathcal{V} = \left\{ c_{\mathbf{i},l} \in \mathcal{C} \,\middle|\, (c_{\mathbf{i},l} \in \mathcal{V}_n, t(c_{\mathbf{i},l}, \mathcal{L}_n) = 3) \text{ or } \left(c_{\mathbf{i}/2,l-1} \in \mathcal{V}_n, t(c_{\mathbf{i}/2,l-1}, \mathcal{L}_n) < 3\right) \right\} \tag{73}$$

where $\mathcal{V}$ is the optimal valid Particle Cell set for $\mathcal{L}$ and $c_{\mathbf{i}/2,l-1}$ denotes the parent Particle Cell of $c_{\mathbf{i},l}$.

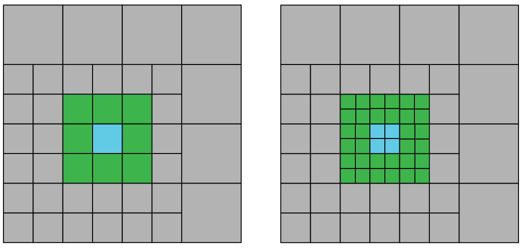

Supplementary Figure 5: Example of the Equivalence property and how the natural Local Particle Cell $\mathcal{L}_n$ set can be used to form $\mathcal{V}_n$, that can then be directly used to compute the OVPC $\mathcal{V}$ for $\mathcal{L}$ using Lemma 3.

In words, $\mathcal{V}$ is constructed by taking all those Particle Cells that have type filler in $\mathcal{V}$, and taking the children of all Particle Cells in $\mathcal{V}_n$ that are of type seed or boundary (Where type is defined relative to $\mathcal{L}_n$). Which means that finding for $\mathcal{V}_n$ with respect to $\mathcal{L}_n$ is equivalent to $\mathcal{V}$ for $\mathcal{L}$. This is useful because, $\#\mathcal{L} > \#\mathcal{L}_n$ and the maximum level l^n_{max} in $\mathcal{L}_n$ is one level less than l_{max} of $\mathcal{L}$ by construction (See Supplementary Note: 5). See Supplementary Figure 5 provides an example in 2D. *Proof*:
Here we need to show that if $\mathcal{V}_n$ is the OVPC for $\mathcal{L}_n$ and we define

$$\hat{\mathcal{V}}(\mathcal{V}_n) = \left\{ c_{\mathbf{i},l} \in \mathcal{C} \,\middle|\, (c_{\mathbf{i},l} \in \mathcal{V}_n, t(c_{\mathbf{i},l}, \mathcal{L}_n) = 3) \text{ or } \left(c_{\mathbf{i}/2,l-1} \in \mathcal{V}_n, t(c_{\mathbf{i}/2,l-1}, \mathcal{L}_n) < 3\right) \right\} \tag{74}$$

then $\hat{\mathcal{V}} = \mathcal{V}$ where $\mathcal{V}$ is the OVPC for $\mathcal{L}$. We do this by relying on Lemma 1. That we can decompose our solution of

$$\mathcal{V}_n = \text{minhull}\left(\bigcup_{c^l_{\mathbf{i},l} \in \mathcal{L}_n} \mathcal{V}^*(c^l_{\mathbf{i},l}) \right) \tag{75}$$

and

$$\mathcal{V} = \text{minhull}\left(\bigcup_{c^l_{\mathbf{i},l} \in \mathcal{L}} \mathcal{V}^*(c^l_{\mathbf{i},l}) \right) \tag{76}$$

then since by construction for $c^*_{\mathbf{i},l} \in \mathcal{L}$ there exists a $c^{*n}_{\mathbf{i},l} \in \mathcal{L}_n$ such that $c^*_{\mathbf{i},l} \in D(c^{*n}_{\mathbf{i},l})$, and $l(c^*_{\mathbf{i},l}) = l(c^{*n}_{\mathbf{i},l}) + 1$ that if we can show that $\hat{\mathcal{V}}(\mathcal{V}^*(c^{n*}_{\mathbf{i},l})) = \mathcal{V}^*(c^*_{\mathbf{i},l})$ then

$$\begin{aligned}\mathcal{V} &= \text{minhull}\left(\bigcup_{c^l_{\mathbf{i},l} \in \mathcal{L}_n} \hat{\mathcal{V}}(\mathcal{V}^*(c^l_{\mathbf{i},l})) \right) \\ &= \hat{\mathcal{V}}\left(\text{minhull}\left(\bigcup_{c^l_{\mathbf{i},l} \in \mathcal{L}_n} \mathcal{V}^*(c^l_{\mathbf{i},l}) \right) \right) \\ &= \hat{\mathcal{V}}(\mathcal{V}_n)\end{aligned} \tag{77}$$

the operations can be taken out of the union and minhull, due to the operation always taking the smallest Particle Cell and the direct correspondence between the two sets.

Therefore, we consider such a $\mathcal{L} = \{c^*_{\mathbf{i},l}\}$ and $\mathcal{L}_n = \{c^{*n}_{\mathbf{i},l}\}$ and consider $\mathcal{V}_n = \mathcal{V}^*(c^{*n}_{\mathbf{i},l})$ and $\bar{\mathcal{V}} = \hat{V}(\mathcal{V}^*(c^{*n}_{\mathbf{i},l}))$. Now lets assume that $\mathcal{V}_n$ is valid and optimal solution with respect to $\mathcal{L}_n$, and assume that $\bar{\mathcal{V}}$ is not valid with respect to $\mathcal{L}$.

If $\bar{\mathcal{V}}$ in not valid, then there must exist $c^v_{\mathbf{i},l} \in \bar{\mathcal{V}}$ such that

$$c^*_{\mathbf{i},l} \in \mathcal{ND}(c^v_{\mathbf{i},l}). \tag{78}$$

Given this holds, then we consider the validity of $\mathcal{V}_n$ with respect to $\mathcal{L}_n$. We treat this in two cases.

First, suppose that $l(c^*_{\mathbf{i},l}) < l(c^v_{\mathbf{i},l}) + 1$, and $c^v_{\mathbf{i},l} \in \mathcal{V}_n$ which implies that $l(c^{n*}_{\mathbf{i},l}) < l(c^v_{\mathbf{i},l})$. Also, given $c^*_{\mathbf{i},l} \in D(c^{n*}_{\mathbf{i},l})$ then

$$c^{n*}_{\mathbf{i},l} \in \mathcal{ND}(c^v_{\mathbf{i},l}). \tag{79}$$

If $c^{vn}_{\mathbf{i},l} \in \mathcal{V}_n$, where $c^v_{\mathbf{i},l}$ is the child of $c^{vn}_{\mathbf{i},l}$, then since $\mathcal{ND}(c^v_{\mathbf{i},l}) \subset \mathcal{ND}(c^{nv}_{\mathbf{i},l})$ then also

$$c^{n*}_{\mathbf{i},l} \in \mathcal{ND}(c^{nv}_{\mathbf{i},l}). \tag{80}$$

This leads to $\mathcal{V}_n$ violating Theorem 1 with respect to $\mathcal{L}_n$.

Now in the second case, $l(c^*_{\mathbf{i},l}) = l(c^v_{\mathbf{i},l}) + 1$, implying that $l(c^{n*}_{\mathbf{i},l}) = l(c^v_{\mathbf{i},l})$. However, this implies that $c^{vn}_{\mathbf{i},l} \in \mathcal{V}_n$ as otherwise $t(c^v_{\mathbf{i},l}, \mathcal{L}_n) = 1$ and $l(c^v_{\mathbf{i},l}) = l(c^*_{\mathbf{i},l})$. Therefore again since $\mathcal{ND}(c^v_{\mathbf{i},l}) \subset \mathcal{ND}(c^{nv}_{\mathbf{i},l})$ then also

$$c^{n*}_{\mathbf{i},l} \in \mathcal{ND}(c^{nv}_{\mathbf{i},l}) \tag{81}$$

and $\mathcal{V}_n$ is invalid with respect to $\mathcal{L}_n$.

Therefore, given we assume $\mathcal{V}_n$ is valid w.r.t $\mathcal{L}_n$ then $\bar{\mathcal{V}}$ must also be valid w.r.t $\mathcal{L}$.

The second step is to show that when $\bar{\mathcal{V}}$ is optimal w.r.t $\mathcal{L}$ then also $\mathcal{V}_n$ is to $\mathcal{L}_n$. So again we assume that $\mathcal{V}_n$ is optimal, but $\bar{\mathcal{V}}$ is not. We follow on from the proof of Theorem 2 which gives us that therefore there exists some $\mathcal{W}$ such that $c^v_{\mathbf{i},l} \in \bar{\mathcal{V}}$ and $c^w_{\mathbf{i},l} \in \mathcal{W}$ and $s(c^v_{\mathbf{i},l}) \subset s(c^w_{\mathbf{i},l})$ and hence

$$c^v_{\mathbf{i},l} \in D(c^w_{\mathbf{i},l}). \tag{82}$$

For this there are again two cases, one where $c^v_{\mathbf{i},l} \in \mathcal{V}_n$ and the other where $c^{nv}_{\mathbf{i},l} \in \mathcal{V}_n$ where $c^v_{\mathbf{i},l} \in D(p^{nv})$ and $l(c^{nv}_{\mathbf{i},l}) = l(c^v_{\mathbf{i},l}) - 1$.

First, let us consider the case of $c^c_{\mathbf{i},l} \in \mathcal{V}_n$. We then have that $c^v_{\mathbf{i},l} \in D(c^w_{\mathbf{i},l})$. However, since $c^*_{\mathbf{i},l} \in D(c^{n*}_{\mathbf{i},l})$ and $c^w_{\mathbf{i},l} \notin \bar{\mathcal{V}}$ means that

$$c^*_{\mathbf{i},l} \in \mathcal{ND}(c^w_{\mathbf{i},l}) \tag{83}$$

which directly implies that

$$c^{n*}_{\mathbf{i},l} \in \mathcal{ND}(c^w_{\mathbf{i},l}). \tag{84}$$

However, this would make $\mathcal{V}_n$ not valid.

Now in the second case we have $c^{nv}_{\mathbf{i},l} \in \mathcal{V}_n$, and hence $t(c^{nv}_{\mathbf{i},l}, \mathcal{L}_n) < 3$. So we know that,

$$c^{*n}_{\mathbf{i},l} \in \mathcal{B}(c^{nv}_{\mathbf{i},l}) \tag{85}$$

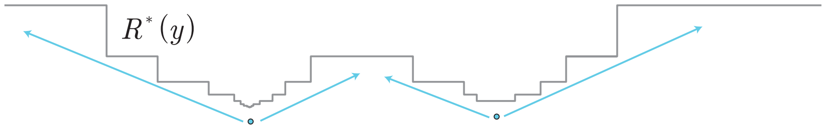

Supplementary Figure 6: The basic concept underlying the Pulling Scheme. R^* is propagated outwards from higher levels to lower levels utilizing property 1 and property 2 of OVPC sets. When two solutions meet, only one needs to be propagated. Therefore, by propagating solutions from l_{max} to l_{min} and propagating the solution to higher levels using the filler type Particle Cells, the solution can be constructed directly, without checking the validity.

such that

$$c^*_{\mathbf{i},l} \in \mathcal{ND}(c^w_{\mathbf{i},l}). \tag{86}$$

This now contradicts that $\mathcal{W}$ can be valid for $\mathcal{L}$. Hence given $\mathcal{V}_n$ is optimal for $\mathcal{L}_n$, $\bar{\mathcal{V}}$ must also be optimal for $\mathcal{L}$.

Now given there is a unique optimal solution then necessarily,

$$\hat{V}(\mathcal{V}^*(c^{*n}_{\mathbf{i},l})) = \mathcal{V}^*(c^{*n}_{\mathbf{i},l}) \tag{87}$$

for any $c^*_{\mathbf{i},l} \in D(c^{*n}_{\mathbf{i},l})$, and $l(c^*_{\mathbf{i},l}) = l(c^{*n}_{\mathbf{i},l}) + 1$, and from our arguments above this leads to $\hat{\mathcal{V}}(\mathcal{V}_n) = \mathcal{V}$ and concludes the proof. $\square$

Additional Algorithm Description

The Pulling Scheme uses all these properties to directly construct $\mathcal{V}$ by propagating solutions from individual Particle Cells in $\mathcal{L}$ using property 1, one level at a time starting from the highest level of the Particle Cells in $\mathcal{L}$. Supplementary Figure 6 shows a schematic of two solutions being propagated from two Particle Cells. When two solutions meet at a Particle Cell, the precedence of one solution depends on the Particle Cells type where they meet. Precedence is ordered from seed>boundary>filler. This order represents the solution that provides the 'tighter' constraint on the resolution function. Then only the solution with precedence needs to be propagated. The Pulling Scheme can be implemented in many different ways here we use an implimentation that uses a data structure that explicitly stores $\mathcal{C}$ the full Particle Cell tree. This is the form of Pulling Scheme used in this paper. However, other forms are possible that do not require the explicit storing of the tree structure. Algorithm 1, gives explicit pseudo-code of the algorithm described in the main text.

Integral neighborhood optimization

From Supplementary Figure 9, we observe that a single $c_{\mathbf{i},l}$ results in a large, high-resolution area in the solution. If we instead take $\mathcal{V}_n$ and create $\mathcal{V}_i$ in the following way

$$\mathcal{V}_i = \left\{ c_{\mathbf{i},l} \in \mathcal{C} \,\middle|\, (c_{\mathbf{i},l} \in \mathcal{V}_n, t(c_{\mathbf{i},l}, \mathcal{L}_n) > 1) \text{ or } \left(c_{\mathbf{i}/2,l-1} \in \mathcal{V}_n, t(c_{\mathbf{i}/2,l-1}, \mathcal{L}_n) = 1\right) \right\} \tag{88}$$

where now boundary Particle Cells are also kept at their original resolution, then, if we use the alternative neighborhood of

```
Data: Local Particle Cell set ℒ
Result: Optimal Valid Particle Cell set 𝒱(ℒ)
Function pulling_scheme(ℒ)
    Represent all possible Particle Cells 𝒞 from l_max to l_min in a multi-resolution pyramid and set all
      Particle Cells type to EMPTY;
    forall Particle Cells c ∈ 𝒞 where c ∈ ℒ do
        c.type = SEED
    end
    for l_c = l_max : l_min do
        /* Fill neighbors (Step 1)                                              */
        forall neighbors n of c ∈ 𝒞(l_c) where c.type is (SEED or PROPAGATE) do
            if n.type is EMPTY then
                n.type = BOUNDARY
        end
        /* Set Parents (Step 2)                                                 */
        forall parents p of c ∈ 𝒞(l_c) where c.type is (SEED, PROPAGATE, or ASCENDANT) do
            p.type = ASCENDANT
        end
        if l_c > l_min then
            /* Set Ascendant Neighbors (Step 3)                                 */
            forall neighbors n of c ∈ 𝒞(l_c − 1) where c.type is ASCENDANT do
                if n.type is EMPTY then
                    n.type = ASCENDANT_NEIGHBOR
                if n.type is SEED then
                    n.type = PROPAGATE
            end
            /* Set Fillers (Step 4)                                             */
            forall children d of c ∈ 𝒞(l_c − 1) where c.type is (ASCENDANT_NEIGH or PROPAGATE)
              do
                if (d.type is EMPTY then
                    d.type = FILLER
            end
    end
    return all type SEED, BOUNDARY and FILLER Particle Cells in 𝒞 as 𝒱;
```

Algorithm 1: The Pulling Scheme algorithm. The Pulling Scheme efficiently computes the OVPC set $\mathcal{V}$ from the Local Particle Cell set $\mathcal{L}$ using a temporary pyramid mesh data structure. $\mathcal{C}(l)$ denotes all Particle Cells on level l. See Supplementary Note: 7 for a schematic of the main steps.

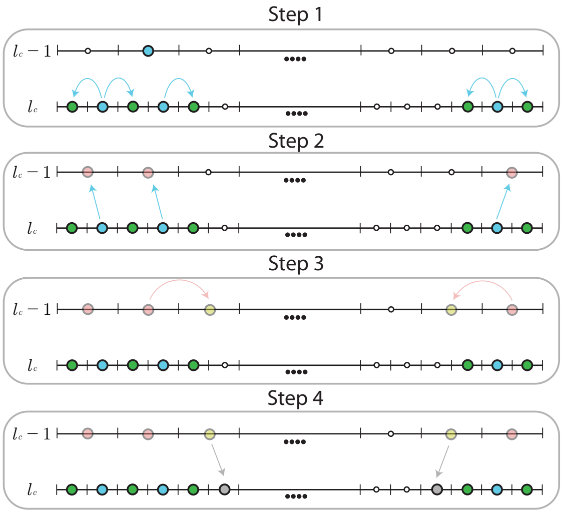

Supplementary Figure 7: Schematic illustrating the four different steps in Algorithm 1 in Supplementary Note: 5 for the Pulling Scheme. The colour of the dots, identifies the type of Particle Cell. Blue dots represent seed, boundary in green, filler in grey, ascendant in red, ascendant neighbour in yellow. These four steps occur on each level from the highest level l_{max} to lowest l_{min}. Step 1, seed Particle Cells, or propogate, add neighbour Particle Cells as boundaries on level l_c. Step 2, seed and ascendant Particle Cells set their parents $(l_c - 1)$ to ascendant. Step 3, the ascendant particles on $l_c - 1$ set their vacant neighbours to ascendant neighbours. Step 4, Particle Cells of type ascendant neighbours and propogate on level $l_c - 1$ set empty children in l_c to filler.

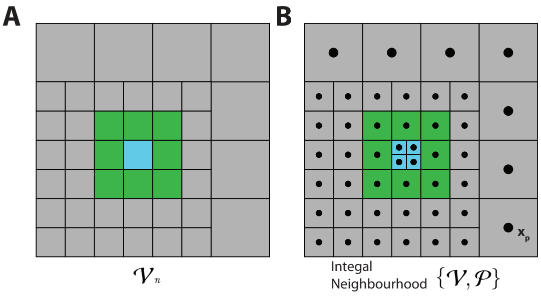

Supplementary Figure 8: **A** the nOVPC $\mathcal{V}_n$ generated using $\mathcal{L}_n$ and the corresponding $\mathcal{V}$ and **B** particle sampling $\mathcal{P}$ for the integral neighborhood optimization that has been used in for the results in this paper.

$$\mathcal{N}(\mathbf{y},R(\mathbf{y}))_i = \{\mathbf{x} \in \Omega : |(\mathbf{y}-\mathbf{x})| \int_0^1 \frac{1}{R(\mathbf{y}+s(\mathbf{x}-\mathbf{y}))} ds \leq 1\} \tag{89}$$

for the representation of the function as in Supplementary Equation 17, instead of the isotropic neighborhood, then $R^*(\mathbf{y},\mathcal{V}_i)$ will also satisfy the Reconstruction Condition. This then results in a smaller $\mathcal{P}^*$ as shown in Supplementary Figure 2. In practice this results in a $\approx 10-30\%$ reduction in $\#\mathcal{V}$, and is used for the 3D APR implimentation used in this paper.

As in the isotropic neighborhood case, we can sample directly using $\mathcal{V}_n$, with only slight adjustment

$$\mathbf{x}_p(c_{\mathbf{i},l}) = \begin{cases} \prod_{\mathbf{i}}\{\frac{\Omega^*}{2^l}(i_k+1/4), \frac{\Omega^*}{2^l}(i_k+3/4)\} & t(c_{\mathbf{i},l}) = \{1\} \\ \{\frac{\Omega^*}{2^l}(i_k+1/2)\} & t(c_{\mathbf{i},l}) = 2,3 \end{cases} \tag{90}$$

Now we briefly show that Reconstruction Condition is satisfied for the integral neighborhood definition and $R^*(\mathcal{V}_i,\mathbf{y})$.

We have the integral interaction neighborhood

$$\mathcal{N}(\mathbf{y},R(\mathbf{y}))_i = \{\mathbf{x} \in \Omega : |(\mathbf{y}-\mathbf{x})| \int_0^1 \frac{1}{R(\mathbf{y}+s(\mathbf{x}-\mathbf{y}))} ds \leq 1\} \tag{91}$$

and show that if we are using the local resolution estimate $L(\mathbf{y}) = \frac{E\sigma(\mathbf{y})}{|\nabla f(\mathbf{y})|}$ that this neighborhood guarantees satisfaction of the Reconstruction Condition, given that $R(\mathbf{y}) \geq L(\mathbf{y})$ and the assumption on the Local Intensity Scale $\sigma(\mathbf{y})$ being sufficiently smooth over the integral path that $\sigma(\mathbf{y}) \approx \sigma(\mathbf{x})$ can be used.

Starting from the following bound as presented above,

$$\epsilon(\mathbf{y}) \leq \sum_{p \in \mathcal{N}(\mathbf{y},R(\mathbf{y}))} |(\mathbf{y}-\mathbf{x}_p)| \int_0^1 |\nabla f(\mathbf{y}+s(\mathbf{x}_p-\mathbf{y}))| ds \xi_p(\mathbf{y}) \tag{92}$$

which we wish to satisfy the Reconstruction Condition, so

$$E\sigma(\mathbf{y}) \geq \sum_{p \in \mathcal{N}(\mathbf{y},R(\mathbf{y}))} |(\mathbf{y}-\mathbf{x}_p)| \int_0^1 |\nabla f(\mathbf{y}+s(\mathbf{x}_p-\mathbf{y}))| ds \xi_p(\mathbf{y}) \tag{93}$$

which we can re-write as

$$\frac{1}{E\sigma(\mathbf{y})} \sum_{p \in \mathcal{N}(\mathbf{y},R(\mathbf{y}))} |(\mathbf{y}-\mathbf{x}_p)| \int_0^1 |\nabla f(\mathbf{y}+s(\mathbf{x}_p-\mathbf{y}))| ds \xi_p(\mathbf{y}) \leq 1 \tag{94}$$

and substituting for $L(\mathbf{y})$ and assuming $\sigma(\mathbf{y})$ is $O(1)$ over the interval gives

$$\sum_{p \in \mathcal{N}(\mathbf{y},R(\mathbf{y}))} |(\mathbf{y}-\mathbf{x}_p)| \int_0^1 \frac{1}{L(\mathbf{y}+s(\mathbf{x}_p-\mathbf{y}))} ds \xi_p(\mathbf{y}) \leq 1 \tag{95}$$

now given our reconstruction kernel conditions, this will hold if for every point,

$$|(\mathbf{y}-\mathbf{x}_p)| \int_0^1 \frac{1}{L(\mathbf{y}+s(\mathbf{x}_p-\mathbf{y}))} ds \leq 1 \tag{96}$$

now given the assumption that $R(\mathbf{y}) \leq L(\mathbf{y})$ then the above will hold if the following also holds

$$|(\mathbf{y}-\mathbf{x}_p)| \int_0^1 \frac{1}{R(\mathbf{y}+s(\mathbf{x}_p-\mathbf{y}))} ds \leq 1 \tag{97}$$

which is the integral interaction neighborhood stated above.

Computational and memory complexity

Here we address the computational and memory complexity of the Pulling Scheme using explicitly storage of $\mathcal{C}$ as described above. We will discuss the equivalence optimized version described in the previous section as this is used in practice.

First we consider the size of $\mathcal{C}$, for a given problem with l_{min} and l_{max}, where $N = 2^{dl_{max}}$, then storing $\mathcal{C}$ requires a data-structure with

$$\begin{aligned} N_{\mathcal{C}} &= \sum_{l=l_{min}}^{l_{max}-1} 2^{dl} \\ &= \frac{2^{d(l_{max}+1)} - 2^{dl_{min}}}{(2^d - 1)} \end{aligned} \tag{98}$$

entries, because the highest level in the structures is $l_{max} - 1$. If we then consider the ratio of the size of the data-structure to the original data size N, we get

$$\frac{N_{\mathcal{C}}}{N} \leq \frac{1}{2^d - 1}\left(1 - \frac{1}{N}\right) \tag{99}$$

where we have set $l_{min} = 0$ as a worst case. Therefore, in the large N limit, we get $\frac{N_{\mathcal{C}}}{N} \approx \frac{1}{2^d-1}$. Which gives us upper bounds of N in 1D, $\frac{N}{3}$ in 2D, and $\frac{N}{7}$ in 3D for the size of the required data-structure. Given there are only seven unique values that are needed for the algorithm, then each only requires 3 bits of information to be stored. Although this is not likely in practice, due to available data types, the Pulling Scheme requires at most $\frac{3N}{2^d-1}$ bits in memory.

For the worst-case computational complexity, we can consider $\mathcal{L}^* = \mathcal{C}^*$, where $\mathcal{C}^*$ is $\mathcal{C}$ restricted to $l_{max} - 1$. That is every Particle Cell is in $\mathcal{L}^*$. Now each, step requires iteration over the data-structure given $\mathcal{O}(N_{\mathcal{C}})$ operations. All parent and neighbour operations scale with dimension d, and therefore for fixed d, have a fixed cost. Therefore, again we can get an upper bound on all steps taken across the algorithm described in Algorithm 1 in Supplementary Note: 5 are $\mathcal{O}(N_{\mathcal{C}})$. Therefore, the whole algorithm is worst-case $\mathcal{O}(N_{\mathcal{C}})$ which is $\mathcal{O}(N)$.

In practice, the performance of this algorithm is more complicated depending on N, $\mathcal{L}$ and the spatial distribution of Particle Cells. From the steps above, we can see that the number neighbor searches at the highest resolution are the number of seed Particle Cells at that level. Further, if we ignore the PROPOGATE nodes, the most costly steps scale with the number of seed Particle Cells ($\#(\mathcal{L} \cap \mathcal{V})$). This is compared to the neighbor and filler Particle Cells that incur proportionally fewer operations. Hence tentatively we would expect the performance to scale as $\mathcal{O}(N + \#(\mathcal{L} \cap \mathcal{V}))$, with the different term dominating depending on situation. Further, the exact cost also would depend on the spatial distribution of $\mathcal{L}$.

Supplementary Note 6: Particle sampling

As in the 1D case, the last step given $\mathcal{V}$, is to determine the particle locations and sample them forming $\mathcal{P}^*$ and the APR. In general dimension, we take the identical approach to 1D. The set of points in $\mathcal{P} = \{\mathbf{x}_p\}_{p=1}^{N_p}$ are chosen such that for each Particle Cell $c_{\mathbf{i},l} \in \mathcal{P}$ a particle p is added to $\mathcal{P}$ as

$$\mathbf{x}_p(c_{\mathbf{i},l}) = \{\frac{\Omega^*}{2^l}(i_k + 1/2)\} \tag{100}$$

for $i_k = 1, .., d$ and $N_p = \#\mathcal{V}$. The function, is then sampled at locations $f_p = f(\mathbf{x}_p)$ to form $\mathcal{P}^* = \{f_p\}_{p=1}^{N_p}$. Such a sample satisfies the requirement that $\#(\mathbf{x}_p \in \mathcal{N}(\mathbf{y}, R(\mathbf{y}))) > 0$. Supplementary Figure 9, shows an

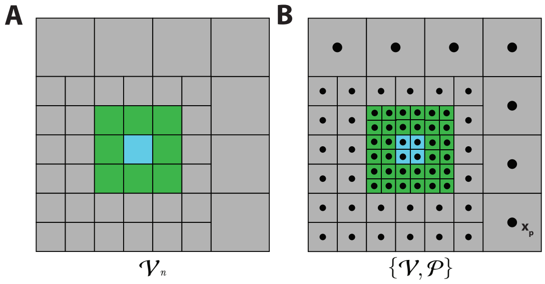

Supplementary Figure 9: **A** the nOVPC $\mathcal{V}_n$ generated using $\mathcal{L}_n$ and the corresponding $\mathcal{V}$ and **B** particle sampling $\mathcal{P}$.

example of $\mathcal{V}_n$ in **A** and then $\mathcal{V}$ and $\mathcal{P}$ **B**. $\#(\mathbf{x}_p \in \mathcal{N}(\mathbf{y}, R(\mathbf{y})))$ is in all cases greater then one, with maximum resolution areas, producing a local grid identical to a pixel image representation. If, different constrainsts on the reconstruction function are required, i.e. a different number of particles, or different layout, these could also be used. Here, we again present the simplest case.

Optimality

Although simple, such a sampling is also in a sense optimal for a given Implied Resolution Function. We define an optimal sampling of a given $R(\mathbf{y})$ as the sampling that satisfies

$$\#\mathcal{P} = \int_\Omega \frac{1}{R(\mathbf{y})^d} d\mathbf{y} \tag{101}$$

and $\#(\mathbf{y} \in \mathcal{N}(\mathbf{y}, R(\mathbf{y}))) > 0$ for all $\mathbf{y} \in \Omega$. Intuitively, if we consider $\frac{1}{R(y)}^d$ as the point-wise required density (defined now for the 'hyper-volume' dependent on the dimension d), then again ignoring edge effects, this means that satisfying Supplementary Equation 101 leads to this density being everywhere exactly realized. Further, this integral is also satisfied for a constant regular sampling such as pixels.

If we consider, the integral Supplementary Equation 101 for the implied resolution function $R^*(\mathbf{y})$, as

$$\int_\Omega \frac{1}{R^*(\mathbf{y})^d} dy = \int_\Omega \frac{1}{\left(\sum_{c_{\mathbf{i},l} \in \mathcal{V}} \phi(\mathbf{y}, c_{\mathbf{i},l}) \frac{\Omega}{2^l}\right)^d} d \tag{102}$$

$$y$$

$$= \sum_{c_{\mathbf{i},l} \in \mathcal{V}} 1$$

$$= \#\mathcal{V} = \#\mathcal{P} \tag{103}$$

as required, and therefore $\mathcal{P}^*$ is optimal in the sense of Supplementary Equation 101. Hence, the Pulling Scheme in addition to providing an optimal Implied Resolution Function also provides an inherent 'optimal' sampling in general dimension.

APR as $\{\mathcal{V}_n, \mathcal{P}^*\}$

From Supplementary Figure 9 and Lemma 3, there is a redundancy in directly storing $\mathcal{V}$, instead $\mathcal{V}_n$ could be stored along with each $t(c_{\mathbf{i},l})$ for each cell (w.r.t $\mathcal{L}_n$). In this way, the particles could be sampled from $\mathcal{V}_n$

using

$$\mathbf{x}_p(c_{\mathbf{i},l}) = \begin{cases} \prod_{\mathbf{i}}\{\frac{\Omega^*}{2^l}(i_k+1/4), \frac{\Omega^*}{2^l}(i_k+3/4)\} & t(c_{\mathbf{i},l}) = \{1,2\} \\ \{\frac{\Omega^*}{2^l}(i_k+1/2)\} & t(c_{\mathbf{i},l}) = 3 \end{cases} \tag{104}$$

where then the locations of $\mathcal{P}$ are still implicit now from $\mathcal{V}_n$ and their type $t(c_{\mathbf{i},l})$. This representation results in lower memory overhead, but at the cost of complexity, therefore depending on the use-case the simpler combination of $\{\mathcal{V}, \mathcal{P}^*\}$ may be preferable. Such a formulation is used for file-storage in this paper.

Supplementary Note 7: Discrete sampling error and noise

In the above, we have ignored particle considerations of, how do we estimate $\frac{\partial f}{\partial y}$ and the impact of noise. Here we will briefly discuss these issues, including a discussion on the continuous resolution functions. The results here are presented in 1D but apply to the general dimension case.

Discrete sampling

First, we consider the what the ideal sampling of $\frac{\partial f}{\partial y}\{\bar{x}\}$ would be that would allow reconstruction of all $y \in \Omega$ (at off particle locations) then the samples

$$\frac{\partial f}{\partial y}\{x_i\} = \max_{x \in [x_i - h/2, x_i + h/2)} \frac{\partial f}{\partial y}(x) \tag{105}$$

where h is the sampling distance between points for $\bar{x}$. These estimates would guarantee the APR reconstructs the function $y \in \Omega$, and not just at sample locations. This follows from the fact that this would produce an upper bound on the true derivative across every interval.

Impact of noisy Local Resolution Estimate $L(\mathbf{y})$

However, even in noise-free situations, we do not have the ability to sample the derivative directly. Instead, we observe $|\widehat{\frac{\partial f}{\partial x}}| = |\frac{\partial f}{\partial x}| + \epsilon$. Therefore, it is interesting how errors from the estimation of the derivative translate into the violation of the Reconstruction Condition for a given relative error bound E.

Therefore, we consider how an error in $L(y)$ translates into the error in the solution compared to the user-set relative error bound E. That is we assume that instead of $L(y)$ we observe,

$$L^*(y) = \frac{E\sigma(y)}{|\nabla f|(1-\alpha)} \tag{106}$$

where α represents the maximum relative error in $|\frac{\partial f}{\partial x}|$ (We assume here the $0 \geq \alpha < 1$). We need only consider reductions of the magnitude of the gradient as increases will not impact the Reconstruction Condition (they simply increase the resolution wastefully). So then if we consider what the worst-case observed E^* is relative to the desired E for a given α (See below Supplementary Note: 7 for derivation) we get

$$\frac{E^* - E}{E} = \frac{1}{1-\alpha} - 1 \tag{107}$$

where the error is taken to occur at a local maximum of the derivative such that the error has an impact on the solution. Interpreting this, we can see that if $\alpha = 0.1$, i.e. ten percent absolute error in the gradient, then the

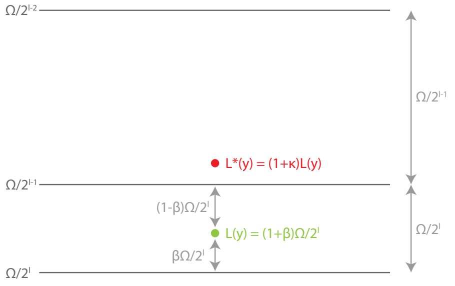

Supplementary Figure 10: Analysis of errors in local resolution estimate $L(\mathbf{y})$

ratio $\frac{E^*-E}{E} = 0.111$, and so if $E = 0.1$ then the observed relative error worst case would be 0.111. So a ten percent error has been related to an eleven percent increase in the realizable relative error.

This bound is insightful, as it tells us that if we have a given α and want to guarantee some realized $\hat{E}$, we can increase the user set E, to retain the bound despite the error (i.e. such that $E^* = \hat{E}$). However, this is at the cost of a higher number of particles. Alternatively, we can re-arrange the bound, as

$$\alpha = 1 - \frac{1}{1 + \frac{(E^*-E)}{E}}. \tag{108}$$

which then tells us how large a relative error in our derivative we can tolerate if we wish to have a set accuracy for the relative error bound.

The analysis above is based on relative errors. How do we then consider absolute errors ϵ? For a given ϵ, the relative error will be greatest with the derivative is small. Interestingly, these are the regions of our solution where it is likely $R^*(y) \leq L(y)$. That is, the large relative error will not impact the solution. The $y \in \Omega$ that are most likely to contribute to $\mathcal{V}$ will have a 'relatively' smaller α due to the larger magnitude of $L(y)$.

Here we derive the above bound for the impact of error in the estimation of $L(\mathbf{y})$ on the ability of the APR to reconstruct the function within the Reconstruction Condition. Now if we suppose that we have the following scenario, as shown in Supplementary Figure 10

$$L(\mathbf{y}) = (1+\beta)\frac{\Omega}{2^l} \tag{109}$$

that is subject to some error, such that the observed local resolution estimate is

$$L^*(\mathbf{y}) = L(\mathbf{y})(1+\kappa) \tag{110}$$

for this error to impact the solution such that it reduces the reconstruction error, the Particle Cell level must decrease, giving a new Particle Cell level $l^* = l - \phi$ which requires

$$L(\mathbf{y})\kappa > (1-\beta)\frac{\Omega}{2^l} \tag{111}$$

which gives us

$$\kappa > \frac{1-\beta}{1+\beta} \tag{112}$$

and that $\phi = \lceil \kappa \rceil$. Now we wish to consider the worse case change in the resolution that would occur, given a particular relative error κ. That is we wish to consider when the error between the true local estimate and the observed quantized value from the particle cell, given by

$$\begin{aligned}\Delta &= \frac{\Omega}{2^{l-\phi}} - L(y) \\ &= \frac{\Omega}{2^l}(2^\phi - 1 - \beta)\end{aligned} \tag{113}$$

is at its largest, which occurs when

$$\kappa = \frac{(2^\phi - 1 - \beta)}{1 + \beta}. \tag{114}$$

Now for the APR we have $L(\mathbf{y}) = \frac{E\sigma(\mathbf{y})}{|\nabla f|}$, and then our observed local resolution estimate can be written as

$$L^*(y) = \frac{E\sigma(\mathbf{y})}{|\nabla f|}(1 + \kappa) \tag{115}$$

and if we now assume that the error in the observed $L(y)$ comes from a underestimate of the gradient magnitude we can re-write this as

$$L^*(y) = \frac{E\sigma(\mathbf{y})}{|\nabla f|(1 - \alpha)} \tag{116}$$

where

$$\alpha = 1 - \frac{1}{1 + \kappa}. \tag{117}$$

Now we wish to know how a change in α would impact our observed relative error bound E^*, compared to our desired relative error bound E, that is

$$E^* - E = |\nabla f| \frac{\Omega}{2^l}(2^\phi - 1 - \beta)\frac{1}{\sigma(\mathbf{y})} \tag{118}$$

and therefore the relative error is

$$\frac{E^* - E}{E} = |\nabla f| \frac{\Omega}{2^l}(2^\phi - 1 - \beta)\frac{1}{E\sigma(\mathbf{y})} \tag{119}$$

where we assume that the maximum gradient magnitude occurs at $\mathbf{y}$ within its neighborhood $\mathcal{N}(\mathbf{y}, R(\mathbf{y}))$. Substituting in for the true gradient magnitude $|\nabla f| = \frac{E\sigma}{(1+\beta)\frac{\Omega}{2^l}}$ we have

$$\begin{aligned}\frac{E^* - E}{E} &= \frac{(2^\phi - 1 - \beta)}{1 + \beta} \\ &= \kappa \\ &= \frac{1}{1 - \alpha} - 1\end{aligned} \tag{120}$$

and similarly

$$\alpha = 1 - \frac{1}{1 + \frac{(E^* - E)}{E}}. \tag{121}$$

This is derived for the error occurring occuring across a particle cell. How does this extend to other Particle Cells? The above analysis has assumed that the error occurs at its maximum value across the whole path. Any paths that cover more than one Particle Cell, will have contributions proportional to the length of the path in each cell. The worst case, would be that this largest relative error occurs everywhere. It is in this case, that this upper bound should hold. Further development of this bound and analysis in the future seems warranted, in addition, to the increase in cost for particles through increases in resolution.

Impact of noisy particles $\hat{f}(y_p)$

Now if we consider that we are only able to sample noisy estimates of the function for our particles, $\hat{f}_p = f(\mathbf{x}_p) + \eta(\mathbf{x}_p)$, where η is some noise process. If we assume that we are still able to estimate $L(\mathbf{y})$ such that R^* is the true optimal solution we will find our observed error is

$$E^* = \frac{|f(\mathbf{y}) - \sum_{\mathbf{x}_p \in \mathcal{N}(\mathbf{y}, R(\mathbf{y}))} (f(\mathbf{x}_p) + \eta(\mathbf{x}_p))\xi_p(y)|}{\sigma(\mathbf{y})} \tag{122}$$

then given $L(\mathbf{y})$ is the noise-free solution then the Reconstruction Condition holds and we get

$$E^* = |A + \frac{1}{\sigma(\mathbf{y})} \sum_{\mathbf{x}_p \in \mathcal{N}(\mathbf{y}, R(\mathbf{y}))} (\mathbf{x}_p)\xi_p(y)| \tag{123}$$

where we have $|A| \leq E$. Therefore as $E \to 0$, $A \to 0$ and therefore the infinity norm of the observed relative error $|E^*|_\infty$ will tend to the maximum $|\frac{1}{\sigma(\mathbf{y})} \sum_{\mathbf{x}_p \in \mathcal{N}(\mathbf{y}, R(\mathbf{y}))} \eta(\mathbf{x}_p)\xi_p(y)|$ across the domain.

Therefore, the noisy input data, provides an upper bound of the observed relative error E^* regardless of adaptation, and user set E. Note, we provide a more detailed analysis of the impact of noise in the following section Supplementary Note: 7 showing that the APR converges at an optimal rate to a bias estimate of the noise-free APR given a non-trivial Resolution Function that satisfies the Resolution Bound. This is consistent with the simple analysis here.

Convergence rate of MSE of APR

Next we consider the case of again known $R(\mathbf{y})$ and the convergence behavior of the MSE of the APR as the number of input samples N that the APR can be estimated from is increased, aligning with the analysis of wavelet thresholding in [5].

We first provide the results, and then the derivation follows. If we assume that $R^*(\mathbf{y})$ satisfies the Reconstruction Condition for parameter $E = \epsilon$ and particles values are estimated by the original Gaussian distributed noisy samples in $R(\mathbf{y})$ of every particle, then the APR will have the following properties as the total sampling N increases:

1. Reconstruction at particle locations from the image follows, $|\mathbb{E}[\hat{f}\{\mathbf{x}_p\} - f(\mathbf{x}_p)]| < \frac{1}{2^{1/d}}$ and $\text{Var}[\hat{f}\{\mathbf{x}_p\} - f(\mathbf{x}_p)] \leq \frac{\sigma}{NC_p}$

2. Reconstruction using the noisy particles at arbitrary locations follows $|\text{E}[\hat{f}(\mathbf{y}) - f(\mathbf{y})]| < (1 + \frac{1}{2^{1/d}})\epsilon$ with $\text{Var}[\hat{f}\{\mathbf{x}_p\} - f(\mathbf{x}_p)] \leq \frac{A_0\sigma^2}{N}$, i.e. an introduced error factor of $\frac{1}{2^{1/d}}\epsilon$

3. The expected MSE of the reconstruction follows $\mathcal{R}(\hat{f}, f, N) \leq \frac{A_1\sigma^2}{N} + ((1 + \frac{1}{2^{1/d}})\epsilon)^2$

where C_p is a constant that depends on the size of $R(\mathbf{x}_p)$, A_0 a constant that depends on the Resolution Function around $\mathbf{y}$ and the reconstruction method and sampling used. Lastly A_1 depends on the Resolution

Function and reconstruction method across the domain. It is worth noting that the bound $(1 + \frac{1}{2^{1/d}})\epsilon$ is not tight and given assumptions on function within $R(\mathbf{y})$ of y, and better than worst-case reconstruction this could be reduced to be closer to ϵ. Effectively the bias introduced into the representation is a result of the spatial average used to estimate the particles.

Hence, we can see that assuming an $R(y)$ that satisfies the Resolution Bound, guarantees optimal convergence to a solution that is within bounded distance of our noise-free reconstruction. However, we have assumed that for the given APR that $R^*(\mathbf{y})$ satisfies the Reconstruction Condition and Resolution Bound. However, with noisy function input, this would not be guaranteed to hold. However, if knowledge of the relative error bounds of estimation of $L(y)$ are known, then the above results could be adapted using to incorporate the results on noisy adaptation in Supplementary Note: 7.

Lets consider a reconstruction of the APR, where we assume that $R(y)$ is noise free, either a continuous solution satisfying the Resolution Bound, or the implied Resolution Function $R^*(y)$ for an APR with $\sigma(y) = 1$ and relative error $E = \epsilon$ (to avoid confusion with the expectations below), where we have estimated the particle intensity values $\hat{f}(\mathbf{x}_p)$ from some noisy sampling $g\{\bar{\mathbf{x}}\}$, with N points, estimated as

$$\hat{f}(\mathbf{x}_p) = \frac{1}{\sum_{\mathbf{x}\in\mathcal{N}(\mathbf{y},R(\mathbf{y}))}} \sum_{\mathbf{x}\in\mathcal{N}(\mathbf{x}_p,R(\mathbf{x}_p))} g\{\mathbf{x}\} \tag{124}$$

that is simply the weighted sum of all points in $g\{\bar{\mathbf{x}}\}$ within $R(y)$ of $\mathbf{x}_p$. As we have done in the benchmark results above. We assume, as above, that each value of g can be decomposed as

$$g\{x\} = f(x) + \eta(x) \tag{125}$$

where $\eta(x) \sim \mathcal{N}(0, \sigma)$. That is, each value is normally distributed with zero mean and standard deviation σ, and the process is independent of each x. For comparison with the wavelet results in [5] we are interested in the statistical properties of the estimate $\hat{f}(\mathbf{x}_p)$ as $N \to \infty$.

First, let's consider the expected value of the error in the estimated particle intensity,

$$\mathbb{E}[\hat{f}(\mathbf{x}_p) - f(\mathbf{x}_p)] = \mathbb{E}[\frac{1}{\sum_{\mathbf{x}\in\mathcal{N}(\mathbf{y},R(\mathbf{y}))}} \sum_{\mathbf{x}\in\mathcal{N}(\mathbf{y},R(\mathbf{y}))} g\{\mathbf{x}\} - f(\mathbf{x}_p)] \tag{126}$$

now let us consider a further decomposition of this sum in the following way

$$\mathbb{E}[\hat{f}(\mathbf{x}_p) - f(\mathbf{x}_p)] = \mathbb{E}[\frac{1}{\sum_{\mathbf{x}\in\mathcal{N}(\mathbf{y},R(\mathbf{y}))}} \sum_{\mathbf{x}\in\mathcal{N}(\mathbf{y},R(\mathbf{y}))} (f(\mathbf{x}_p) + h(\mathbf{x}) + \eta(\mathbf{x}))] - f(\mathbf{x}_p) \tag{127}$$

where we use 125, and we define $h(\mathbf{x})$ by decomposing the noisy function component in terms of $g\{\mathbf{x}\} = f(\mathbf{x}_p) + h(\mathbf{x}) + \eta(\mathbf{x})$. Now given that the expectation is linear, we can isolate the random variable, giving us

$$\begin{aligned}\mathbb{E}[\hat{f}(\mathbf{x}_p) - f(\mathbf{x}_p)] = &\frac{1}{\sum_{\mathbf{x}\in\mathcal{N}(\mathbf{y},R(\mathbf{y}))}} \sum_{\mathbf{x}\in\mathcal{N}(\mathbf{y},R(\mathbf{y}))} h(\mathbf{x}) \\ &+ \frac{1}{\sum_{\mathbf{x}\in\mathcal{N}(\mathbf{y},R(\mathbf{y}))}} \sum_{\mathbf{x}\in\mathcal{N}(\mathbf{y},R(\mathbf{y}))} \mathbb{E}[\eta(\mathbf{x})]\end{aligned} \tag{128}$$

now given that each $\eta(x)$ is independent and identically distributed (i.i.d) with mean zero, then,

$$\mathbb{E}[\hat{f}(\mathbf{x}_p) - f(\mathbf{x}_p)] = \frac{1}{\sum_{\mathbf{x}\in\mathcal{N}(\mathbf{y},R(\mathbf{y}))}} \sum_{\mathbf{x}\in\mathcal{N}(\mathbf{y},R(\mathbf{y}))} h(\mathbf{x}). \tag{129}$$

here we relate the two as $g(\mathbf{x}) = h(\mathbf{x}) + f(\mathbf{x}_p)$. Now, given that $R(y)$ satisfies the Reconstruction Condition for ϵ, and let $M = \frac{1}{\sum_{\mathbf{x}\in\mathcal{N}(\mathbf{y},R(\mathbf{y}))}}$ (the inverse of the number of sample points used in the neighborhood), then we can then bound this as,

$$
\begin{aligned}
|\mathbb{E}[\hat{f}(\mathbf{x}_p) - f(\mathbf{x}_p)]| &= |\frac{1}{\sum_{\mathbf{x}\in\mathcal{N}(\mathbf{y},R(\mathbf{y}))}} \sum_{\mathbf{x}\in\mathcal{N}(\mathbf{y},R(\mathbf{y}))} h(\mathbf{x})| \\
&\leq \epsilon\left(\frac{M-1}{M}\right) \qquad (130)
\end{aligned}
$$

where the factor comes from the assumption that $h(\mathbf{x}_p) = 0$. However, given we have also that $R(y)$ satisfies the Resolution Bound in addition to the Reconstruction Condition then the maximum gradient is bounded across the interval. This allows us to get an upper bound on the growth of $h(\mathbf{x})$ by assuming the it is at the worst case the local minimum or maximum of a piece-wise linear (in 1D) sections. Using this upper bound we get the tighter bound that,

$$
\begin{aligned}
|\mathbb{E}[\hat{f}(\mathbf{x}_p) - f(\mathbf{x}_p)]| &= |\frac{1}{\sum_{\mathbf{x}\in\mathcal{N}(\mathbf{y},R(\mathbf{y}))}} \sum_{\mathbf{x}\in\mathcal{N}(\mathbf{y},R(\mathbf{y}))} h(\mathbf{x})| \\
&\leq \frac{\epsilon}{2^{1/d}} \qquad (131)
\end{aligned}
$$

where d is the dimension. Hence, our estimate will converge to a value within $\frac{\epsilon}{2^{1/d}}$ of the true value as $N \to \infty$. Now lets consider the variance of this estimate, that is what is the asymptotic behavior of the MSE of our estimate as again $N \to \infty$. So we have

$$
\mathrm{Var}[\hat{f}(\mathbf{x}_p) - f(\mathbf{x}_p)] = \mathrm{Var}[\frac{1}{\sum_{\mathbf{x}\in\mathcal{N}(\mathbf{y},R(\mathbf{y}))}} \sum_{\mathbf{x}\in\mathcal{N}(\mathbf{y},R(\mathbf{y}))} g\{\mathbf{x}\} - f(\mathbf{x}_p)] \qquad (132)
$$

which following the same steps as above we get,

$$
\mathrm{Var}[\hat{f}(\mathbf{x}_p) - f(\mathbf{x}_p)] = \mathrm{Var}[\frac{1}{\sum_{\mathbf{x}\in\mathcal{N}(\mathbf{y},R(\mathbf{y}))}} \sum_{\mathbf{x}\in\mathcal{N}(\mathbf{y},R(\mathbf{y}))} \eta(\mathbf{x})] \qquad (133)
$$

which is the variance of the uniformly minimum variance unbiased estimator of the normally random variable η and is therefore

$$
\mathrm{Var}[\hat{f}(\mathbf{x}_p) - f(\mathbf{x}_p)] = \frac{\sigma^2}{M}. \qquad (134)
$$

Now assuming that f has a bounded first derivative, then for sufficiently large M that $M > 0$ such that the above makes sense then

$$
\mathrm{Var}[\hat{f}(\mathbf{x}_p) - f(\mathbf{x}_p)] = \frac{\sigma^2}{C_0 N}. \qquad (135)
$$

since $R(y)$ defines an isotropic region representing a constant (hyper) volume fraction of the domain, and C_0 is some point dependent constant. Therefore, each estimate $\hat{f}(\mathbf{x}_p)$, converges to within ϵ of $f(\mathbf{x}_p)$, with assymptotic rate of $\frac{1}{N}$.

Next, we consider what we asymptotically get for the expected MSE that we align with [5] and call the risk,

$$\mathcal{R}(\hat{f}, f, N) = \mathbb{E}[\frac{1}{N}\sum_{i=1}^{N}(\hat{f}(\mathbf{x}_i) - f(\mathbf{x}_i))^2] \tag{136}$$

$$= \frac{1}{N}\sum_{i=1}^{N}\mathbb{E}[(\hat{f}(\mathbf{x}_i) - f(\mathbf{x}_i))^2] \tag{137}$$

now using the fact that $\mathbb{E}[X^2] = \mathrm{Var}[X] + (\mathbb{E}[X])^2$, we get,

$$\mathcal{R}(\hat{f}, f, N) = \frac{1}{N}\sum_{i=1}^{N}\Big(\mathrm{Var}[(\hat{f}(\mathbf{x}_i) + f(\mathbf{x}_i))] + (\mathbb{E}[(\hat{f}(\mathbf{x}_i) - f(\mathbf{x}_i))])^2\Big) \tag{138}$$

and now using the same steps from Supplementary Equation 130 and Supplementary Equation 135 above,

$$\mathcal{R}(\hat{f}, f, N) = \frac{\sigma^2}{N^2}\sum_{i=1}^{N}\frac{1}{C_i} + \frac{1}{N}\sum_{i=1}^{N}\left(\frac{1}{\sum_{\mathbf{x}\in\mathcal{N}(\mathbf{y},R(\mathbf{y}))}}\sum_{\mathbf{x}\in\mathcal{N}(\mathbf{y},R(\mathbf{y}))} h(\mathbf{x})\right)^2 \tag{139}$$

where C_i is a point dependent volume scaling constant. Now we using the fact that $R(\mathbf{y})$ follows the Reconstruction Condition we have

$$\mathcal{R}(\hat{f}, f, N) \leq \frac{\sigma^2}{N^2}\sum_{i=1}^{N}\frac{1}{C_i} + \frac{\epsilon^2}{2^{2/d}} \tag{140}$$

Now since C_i are non-zero constants, we can then bound them by $\frac{1}{C_i} \leq A$, such that we get

$$\mathcal{R}(\hat{f}, f, N) \leq \frac{A\sigma^2}{N} + \frac{\epsilon^2}{2^{2/d}} \tag{141}$$

therefore, we see that we have asymptotic convergence to a biased estimator that behaves as $\frac{1}{N}$, which is the optimal rate with convergence that depends on A, which is a function of $R(\mathbf{y})$.

APR reconstruction

In the above, we assumed that for every point in the domain we used $R(\mathbf{y})$ to estimate the point. What if instead we only estimate $f(\mathbf{x}_p)$, and then reconstruct the intensities at the other points, as in the APR. We now repeat the above steps. So now we have

$$\mathbb{E}[f(\mathbf{y}) - \hat{f}(\mathbf{y})] = \mathbb{E}[f(\mathbf{y}) - \sum_{\mathbf{x}_p\in\mathcal{N}(\mathbf{y},R(\mathbf{y}))}\hat{f}\{\mathbf{x}_p\}\xi_p] \tag{142}$$

$$= f(\mathbf{y}) - \sum_{\mathbf{x}_p\in\mathcal{N}(\mathbf{y},R(\mathbf{y}))}\mathbb{E}[\hat{f}\{\mathbf{x}_p\}]\xi_p \tag{143}$$

using our result from Supplementary Equation 130 above, we have

$$\mathbb{E}[f(\mathbf{y}) - \hat{f}(\mathbf{y})] = f(\mathbf{y}) - \sum_{\mathbf{x}_p \in \mathcal{N}(\mathbf{y}, R(\mathbf{y}))} \left(f(\mathbf{x}_p) + \frac{1}{\sum_{\mathbf{x} \in \mathcal{N}(\mathbf{x}_p, R(\mathbf{x}_p))}} \sum_{\mathbf{x} \in \mathcal{N}(\mathbf{x}_p, R(\mathbf{x}_p))} h(\mathbf{x}) \right) \xi_p$$

$$= \sum_{\mathbf{x}_p \in \mathcal{N}(\mathbf{y}, R(\mathbf{y}))} \left(h_1(\mathbf{x}_p) + \frac{1}{\sum_{\mathbf{x} \in \mathcal{N}(\mathbf{x}_p, R(\mathbf{x}_p))}} \sum_{\mathbf{x} \in \mathcal{N}(\mathbf{x}_p, R(\mathbf{x}_p))} h_2(\mathbf{x}) \right) \xi_p \quad (144)$$

where h is defined similarly as above. Now $h_2(\mathbf{x})$ is bounded by $\frac{\epsilon}{2^{1/d}}$, for arbitrary particles and worst case the Reconstruction Condition gurantees $h_1(\mathbf{x}) \leq \epsilon$. Hence we have

$$|\mathbb{E}[f(\mathbf{y}) - \hat{f}(\mathbf{y})]| \leq \sum_{\mathbf{x}_p \in \mathcal{N}(\mathbf{y}, R(\mathbf{y}))} \left(\epsilon + \frac{1}{\sum_{\mathbf{x} \in \mathcal{N}(\mathbf{x}_p, R(\mathbf{x}_p))}} \sum_{\mathbf{x} \in \mathcal{N}(\mathbf{x}_p, R(\mathbf{x}_p))} \frac{\epsilon}{2^{1/d}} \right) \xi_p$$

$$\leq (1 + \frac{1}{2^{1/d}})\epsilon. \quad (145)$$

Therefore, any reconstruction will have a an expected value with bias smaller than $(1 + \frac{1}{2^{1/d}})\epsilon$ at all points $\mathbf{y} \in \Omega$. What is the variance of our estimator? So we have

$$\mathrm{Var}[f(\mathbf{y}) - \hat{f}(\mathbf{y})] = \mathrm{Var}[\sum_{\mathbf{x}_p \in \mathcal{N}(\mathbf{y}, R(\mathbf{y}))} \hat{f}\{\mathbf{x}_p\}\xi_p] \quad (146)$$

$$= \mathrm{Var}[\sum_{\mathbf{x}_p \in \mathcal{N}(\mathbf{y}, R(\mathbf{y}))} \left(\frac{1}{\sum_{\mathbf{x} \in \mathcal{N}(\mathbf{x}_p, R(\mathbf{x}_p))}} \sum_{\mathbf{x} \in \mathcal{N}(\mathbf{x}_p, R(\mathbf{x}_p))} g\{\mathbf{x}\} \right) \xi_p]$$

$$= \mathrm{Var}[\sum_{\mathbf{x}_p \in \mathcal{N}(\mathbf{y}, R(\mathbf{y}))} \left(\frac{1}{\sum_{\mathbf{x} \in \mathcal{N}(\mathbf{x}_p, R(\mathbf{x}_p))}} \sum_{\mathbf{x} \in \mathcal{N}(\mathbf{x}_p, R(\mathbf{x}_p))} \eta(\mathbf{x}) \right) \xi_p] \quad (147)$$

here we have to be careful because the $\eta(\mathbf{x})$ are no longer all independent due to the overlap of the neighborhood causing different original sample points enter the variance multiple times. Therefore we have to take care of these by also considering the covariance between the samples,

$$\mathrm{Var}[f(\mathbf{y}) - \hat{f}(\mathbf{y})] = \sum_{\mathbf{x}_p \in \mathcal{N}(\mathbf{y}, R(\mathbf{y}))} \xi_p^2 \mathrm{Var}[\frac{1}{\sum_{\mathbf{x} \in \mathcal{N}(\mathbf{x}_p, R(\mathbf{x}_p))}} \sum_{\mathbf{x} \in \mathcal{N}(\mathbf{x}_p, R(\mathbf{x}_p))} \eta(\mathbf{x})] +$$

$$2 \sum_{(\mathbf{x}_p, \mathbf{x}_q) \in \mathcal{N}(\mathbf{y}, R(\mathbf{y})) : \mathbf{x}_p \neq \mathbf{x}_q} \xi_p \xi_q \mathrm{Cov}[\frac{1}{\sum_{\mathbf{x}_0 \in \mathcal{N}(\mathbf{x}_p, R(\mathbf{x}_p))}}$$

$$\sum_{\mathbf{x}_0 \in \mathcal{N}(\mathbf{x}_p, R(\mathbf{x}_p))} \eta(\mathbf{x}_0), \frac{1}{\sum_{\mathbf{x}_1 \in \mathcal{N}(\mathbf{x}_q, R(\mathbf{x}_q))}} \sum_{\mathbf{x}_1 \in \mathcal{N}(\mathbf{x}_q, R(\mathbf{x}_q))} \eta(\mathbf{x}_1)] \quad (148)$$

which we evaluate, as the only terms that will be non-zero in the covariance will be for the cases with $\mathbf{x}_0 = \mathbf{x}_1$. If we let $\gamma_{p,q}$ be the number of shared points in the original noisy sampling for the support particles p and q then we get,

$$\mathrm{Var}[f(\mathbf{y}) - \hat{f}(\mathbf{y})] = \sigma^2 \sum_{\mathbf{x}_p \in \mathcal{N}(\mathbf{y}, R(\mathbf{y}))} \frac{1}{\sum_{\mathbf{x} \in \mathcal{N}(\mathbf{x}_p, R(\mathbf{x}_p))}} \xi_p^2 + \quad (149)$$

$$2 \sum_{(\mathbf{x}_p, \mathbf{x}_q) \in \mathcal{N}(\mathbf{y}, R(\mathbf{y})) : \mathbf{x}_p \neq \mathbf{x}_q} \xi_p \xi_q \frac{\gamma_{p,q} \sigma^2}{(\sum_{\mathbf{x}_1 \in \mathcal{N}(\mathbf{x}_q, R(\mathbf{x}_q))})(\sum_{\mathbf{x}_0 \in \mathcal{N}(\mathbf{x}_p, R(\mathbf{x}_p))})} \quad (150)$$

Next, we introduce the constants C_p such that $NC_p = \sum_{\mathbf{x}\in\mathcal{N}(\mathbf{x}_p,R(\mathbf{x}_p))}$, (ignoring complications due to the discrete nature of N) . We then note that $\gamma_{p,q}$ is bounded by the smaller of the number of points in p or q, we shall choose p to be the larger, then we have,

$$\mathrm{Var}[f(\mathbf{y}) - \hat{f}(\mathbf{y})] \leq \frac{\sigma^2}{N}\left(\sum_{\mathbf{x}_p\in\mathcal{N}(\mathbf{y},R(\mathbf{y}))}\frac{\xi_p^2}{C_p} + 2\sum_{(\mathbf{x}_p,\mathbf{x}_q)\in\mathcal{N}(\mathbf{y},R(\mathbf{y})):\mathbf{x}_p\neq\mathbf{x}_q}\frac{\xi_p\xi_q}{C_p}\right) \tag{151}$$

hence, our estimator converges to a biased estimate at the optimal rate of $\frac{1}{N}$ for all $\mathbf{y}\in\Omega$.

So then lastly, we consider the Risk for our reconstruction, that is the expected asymptotic behavior of the MSE, following the steps again as above,

$$\mathcal{R}(\hat{f},f,N) = \mathbb{E}[\frac{1}{N}\sum_{i=1}^{N}(\hat{f}(\mathbf{x}_i) - f(\mathbf{x}_i))^2] \tag{152}$$

$$= \frac{1}{N}\sum_{i=1}^{N}\mathrm{Var}[\hat{f}(\mathbf{x}_i)] + \frac{1}{N}\sum_{i=1}^{N}\Big(\mathbb{E}[\hat{f}(\mathbf{x}_i) - f(\mathbf{x}_i)]\Big)^2 \tag{153}$$

$$\leq \frac{1}{N}\sum_{i=1}^{N}\left(\frac{\sigma^2}{N}\left(\sum_{\mathbf{x}_p\in\mathcal{N}(\mathbf{y},R(\mathbf{y}))}\frac{\xi_p^2}{C_p} + 2\sum_{(\mathbf{x}_p,\mathbf{x}_q)\in\mathcal{N}(\mathbf{y},R(\mathbf{y})):\mathbf{x}_p\neq\mathbf{x}_q}\frac{\xi_p\xi_q}{C_p}\right)\right)$$
$$+ ((1+\frac{1}{2^{1/d}})\epsilon)^2 \tag{154}$$

again we can see that if we bound our constants that are independent of N by some constant A_1 then we have

$$\mathcal{R}(\hat{f},f,N) \leq \frac{A_1\sigma^2}{N} + ((1+\frac{1}{2^{1/d}})\epsilon)^2 \tag{155}$$

therefore, we again have that the MSE will converge with optimal rate $\frac{1}{N}$, to a value with bias $((1+\frac{1}{2^{1/d}})\epsilon)^2 > \epsilon^2$, where ϵ is the user set parameter E.

Noise distribution of APR particles

Following the same procedure as the analysis above for the convergence of the APR for increasing N, we can also compare the noise distribution of the original input pixels ($g(\mathbf{x})$) and the computed particle values ($f(\mathbf{x}_p)$). This depends on both the original noise distribution in the image and technique used to compute particle values from the original pixels. If we assume, as above, that the particle intensities are estimated by the average of all pixels within the particles Particle Cell in the following way

$$\hat{f}(\mathbf{x}_p) = \frac{1}{2^{d(l-l_{max})}}\sum_{\mathbf{x}\in s(c_{\mathbf{i},l})} g\{\mathbf{x}\} \tag{156}$$

where d is the dimension and l is the Particle Cell level of $c_{\mathbf{i},l}$ and the sum is all ($2^{d(l-l_{max})}$) pixels in the Particle Cell. Immediately we observe that for Particle Cells at pixel resolution, that is with $l = l_{max}$ the particle and pixel noise distributions are identical. For particles on lower levels, we have the following general results, again following from above we get the expectation of the distrbution as

$$\mathbb{E}[\hat{f}(\mathbf{x}_p) - f(\mathbf{x}_p)] = \frac{1}{2^{d(l-l_{max})}}\sum_{\mathbf{x}\in s(c_{\mathbf{i},l})} h(\mathbf{x})$$
$$+ \frac{1}{2^{d(l-l_{max})}}\sum_{\mathbf{x}\in s(c_{\mathbf{i},l})}\mathbb{E}[\eta(\mathbf{x})] \tag{157}$$

and variance of

$$\mathrm{Var}[\hat{f}(\mathbf{x}_p) - f(\mathbf{x}_p)] = \mathrm{Var}[\frac{1}{2^{d(l-l_{max})}} \sum_{\mathbf{x} \in s(c_{\mathbf{i},l})} \eta(\mathbf{x})] \tag{158}$$

where $\eta(\mathbf{x})$ is the noise distribution of the original pixel image at $\mathbf{x}$. Further analysis requires information on the distribution $\eta(\mathbf{x})$. We consider two special cases of Gaussian and Poisson noise below.

First, under the assumption that $\eta(\mathbf{x})$ are independent and Gaussian with zero mean and fixed variance σ^2, this is the problem we consider above in Supplementary Note: 7, giving us that the particles will remain Gaussian distributed with variance that depends on the Particle Cell level l given by

$$\mathrm{Var}[\hat{f}(\mathbf{x}_p) - f(\mathbf{x}_p)] = \frac{\sigma^2}{2^{d(l-l_{max})}}, \tag{159}$$

and a bias bounded by $\frac{E}{2^{1/d}}$.

Second, we also consider the case for sufficiently large intensity values such that for a image with Poisson noise the noise can be approximated as being approximately Gaussian distributed with zero mean and pixel dependent variance of $f(\mathbf{x})$. Giving us that

$$\mathrm{Var}[\hat{f}(\mathbf{x}_p) - f(\mathbf{x}_p)] = \frac{1}{2^{2d(l-l_{max})}} \sum_{\mathbf{x} \in s(c_{\mathbf{i},l})} f(\mathbf{x}), \tag{160}$$

that with a bounded error of $\frac{E}{2^{1/d}}$ can be approximated by

$$\mathrm{Var}[\hat{f}(\mathbf{x}_p) - f(\mathbf{x}_p)] = \frac{f(\mathbf{x}_p)}{2^{d(l-l_{max})}}. \tag{161}$$

That is each particle can be approximated by a Gaussian distribution with again a bias mean, and a particle dependent variance that will also depend on the level of the Particle Cell. This is the case for the synthetic images we use here, with results for a synthetic image shown in Supplementary Figure 11.

Supplementary Note 8: Comparison with optimal continuous Resolution Functions

In the formulation of the APR, we have restricted the solution in two main ways. Firstly, by satisfying the more restrictive Resolution Bound instead of simply the Reconstruction Condition, and also by using an Implied Resolution Function $R^*(\mathbf{y})$ instead of a more generation continuous Resolution Function. Below we provide a few results and discussion on the impact of these added restrictions and their impact on the adaptivity of the APR.

Reconstruction Condition vs. Resolution Bound

First, we consider the relationship between the optimal solution to the Reconstruction Condition $R_c(\mathbf{y})$ and optimal solution to the Resolution Bound $R_b(\mathbf{y})$. Since the Resolution Bound is derived from an upper bound on the Reconstruction Condition (given the appropriate assumptions), a Resolution Function satisfying the Resolution Bound also satisfies the Reconstruction Condition. Hence the Reconstruction Condition is a tighter bound on $R(\mathbf{y})$ and $R_c(\mathbf{y}) \leq R_b(\mathbf{y})$ for all $\mathbf{y}$. The difference between R_b and R_c is the results of bounding the error by taking the uniform estimate of the maximum of the gradient across the interval

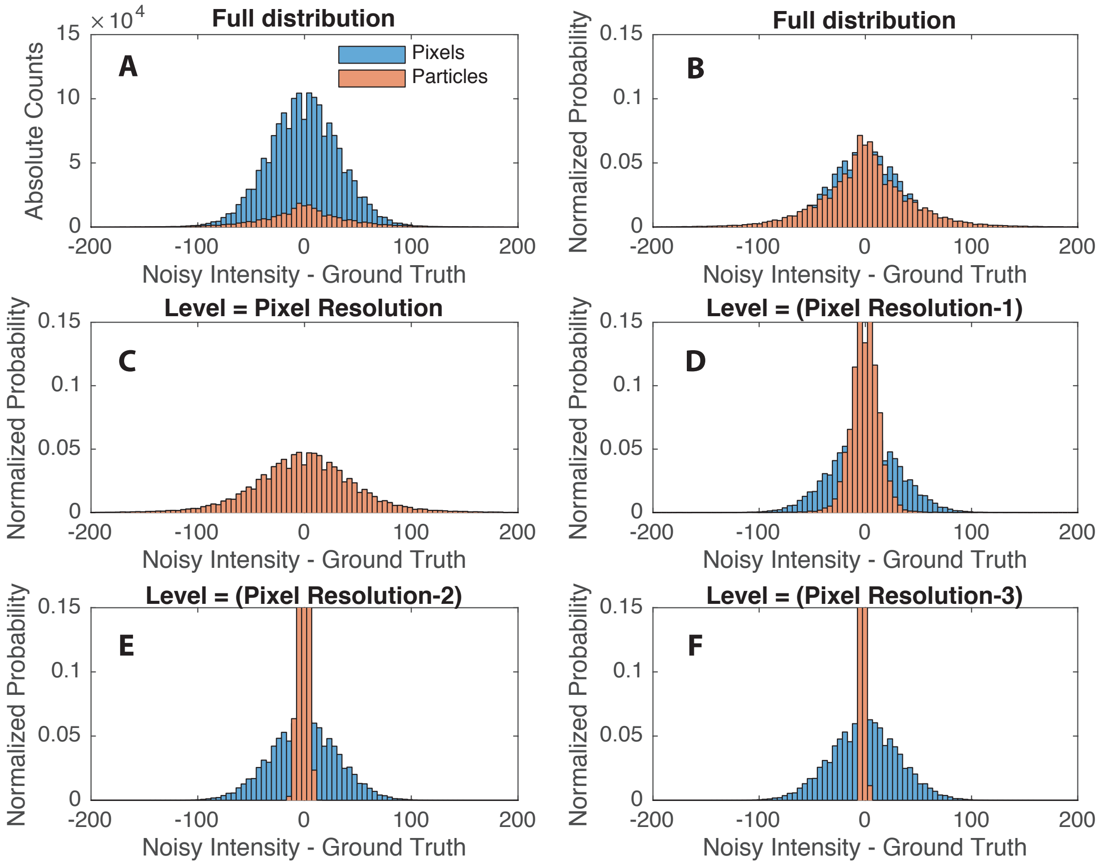

Supplementary Figure 11: The figure compares the noise distribution for a synthetic $N = 120^3$ sphere image with medium noise and blur for the APR ($E = 0.1$) and original image compared to the ground truth. The noise distribution for the original image is a Poisson approximation generated as in Supplementary Note: 15. Histograms **A** and **B** compare the distribution of the noisy pixels (blue), and noisy particles (orange), when subtracted from the ground-truth image. The left plot shows the raw histogram, and on the left the normalized distributions. The remaining four histograms (**C**-**F**) then shows sub-sets of normalized distributions grouped by Particle Cell level for the corresponding particle. For the pixels intensities, all pixel values where used that were contained within Particle Cells of the appropriate level. The **C** distributions are identical, as particles at the maximum level l_{max} are directly the pixel values.

Supplementary Equation 21, instead of the exact path integral in Supplementary Equation 19. Without restrictions on both E and the function f, unfortunately, I know of no upper bound on this difference. However, if we consider f that is infinitely differentiable and the limit as $E \to 0$, we observe that either: f will be constant in some interval and $R(y)$ will also reach some lower bound with a constant zero derivative, or, $R_b(y), R_c(y) \to 0$. In the first case trivially the two bounds are equal. In the second case, since f is assumed to be infinitely differentiable from its Taylor series expansion the difference between $|R_c(y) - R_b(y)| \to 0$ as $R_c(y), R_b(y) \to 0$. Hence, given assumptions in the small E limit, the solutions converge.

Bounds for Implied Resolution Function

Next, we consider what the relationship between $R_b(\mathbf{y})$ and the Implied Resolution Function $R^*(\mathbf{y})$ generated by the Optimal Valid Particle Cell set $\mathcal{V}$. Given that $R_b(\mathbf{y})$ and $R^*(\mathbf{y})$ both satisfy the Resolution Bound, but, $R^*(\mathbf{y})$ is restricted to be piecewise constant then necessarily $R_b(\mathbf{y}) \leq R^*(\mathbf{y})$. The difference between these two solutions represents the loss in adaptation resulting from constructing our solution of Particle Cells instead of allowing continuous adaptation. Since the solutions are both optimal (R^* over restricted solutions) for the Resolution Bound, we can bound the worst case difference between these two solutions.

Let us have some $\mathcal{V}$ that satisfies the Resolution Bound for $L(\mathbf{y})$. We then ask, in the worst case how much larger could R_b be compared to R^*? That is, what is the bound on $\frac{R_b(\mathbf{y})}{R^*(\mathbf{y})}$ given only knowledge of R^*.

We can evaluate this question by considering a bound for all particle cells $c_{\mathbf{i},l} \in \mathcal{V}$. Given that $c_{\mathbf{i},l}$ belongs to the optimal set, from Theorem 2, we know that its parent $c_{\mathbf{i}/2,l}$ must violate Theorem 1. Explicitly, that is

$$\{\mathcal{L} \cap \mathcal{ND}(c_{\mathbf{i}/2,l})\} \supseteq c^n_{\mathbf{i},l}. \tag{162}$$

Now, there are many combinations of $L(\mathbf{y})$ and $R_b(\mathbf{y})$ that could results in that this situation. However, the worst case, i.e. that allowing the largest $R_b(\mathbf{y})$ over the spatial domain of the particle cell is unique (ignoring equivalent configurations). The worst case occurs when $L(\mathbf{y})$ is the largest distance from the particle cell at $\mathbf{y}^*$, and occurs exactly on the interval between two particle cells i.e. $L(\mathbf{y}^*) = \frac{\Omega}{2^{l-1}}$ and for $y^* \in s(c^n_{\mathbf{i},l})$. If we assume that $L(\mathbf{y})$ is a dirac delta, where $\mathbf{y}^*$ is the only non-zero point (again worst case as it provide minimal restriction on the solution). In this way the optimal continuous solution of the Resolution Bound for this is

$$R^*_b(\mathbf{y}, \mathbf{y}^*) \begin{cases} dist(\mathbf{y}, \mathbf{y}^*) & dist(\mathbf{y}, \mathbf{y}^*) \geq L(\mathbf{y}^*) \\ L(\mathbf{y}^*) & dist(\mathbf{y}, \mathbf{y}^*) < L(\mathbf{y}^*) \end{cases} \tag{163}$$

where $dist(.,.)$ is the Euclidean distance between two points. This can be trivially proven by directly considering the solution for an $R^*_b(\mathbf{y}, \mathbf{y}^*) + \delta$ for $\delta > 0$, and noting that the bound no longer holds. We can use this then to consider the direct upper bound, i.e. within $s(c_{\mathbf{i},l})$ how large can $R^*_b(\mathbf{y}, \mathbf{y}^*)$ be? If we consider the distance of the furthest point from $\mathbf{y}^*$ that is in $s(c^n_{\mathbf{i},l})$ we get a worst case of $R^*_b(\mathbf{y}, \mathbf{y}^*) \leq (4\frac{\Omega}{2^l})d^{1/2}$ and hence, we have

$$\frac{R_c(\mathbf{y})}{R^*(\mathbf{y})} \leq 4\sqrt{d} \tag{164}$$

where d is the dimension. Hence this corresponds to ratios bounded by 4, ≈ 5.65 and ≈ 6.93 in 1D, 2D and 3D respectively.

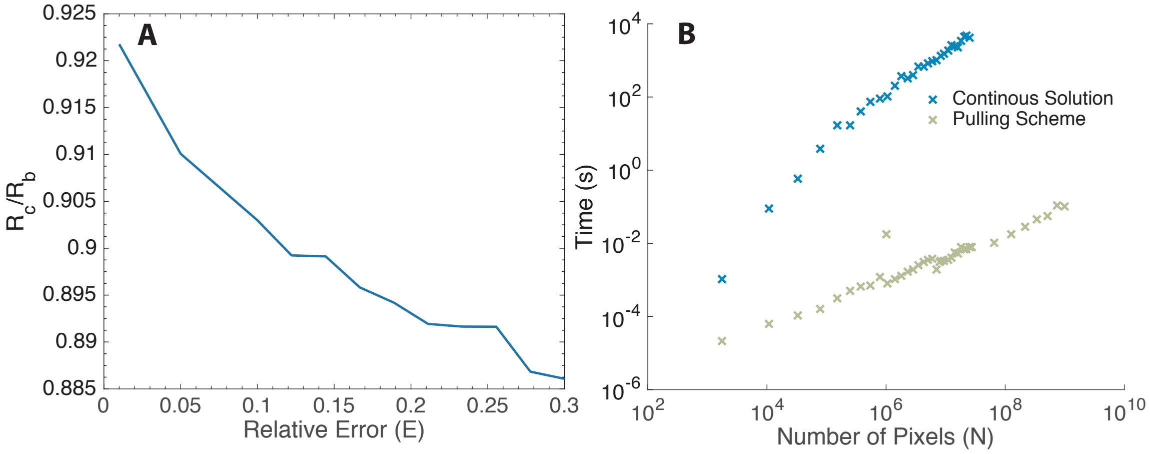

Supplementary Figure 12: **A** shows the ratio of a numerical estimate of the ratio between the optimal continuous solutions to the Resolution Bound R_b and Resolution Condition R_c for a Guassian 1D benchmark problem plotted against the relative error E. We note that smaller values of E resulted in a prohibitive computational cost. **B** shows the relative execution time in 3D of a numerical estimate to R_b and the execution time of the pulling scheme for a fixed ratio content benchmark plotted against increasing number of pixels N. We note that the continuous solution became computationally prohibitive beyond a maximum width of 128 (Beyond which the continuous solution took over 2 hours to estimate compared to less than .01 seconds for the Pulling Scheme).

Bounds on particle sampling

Can we construct a similar bound on the Particle Sampling $\mathcal{P}$. Here we consider samplings restricted to those that satisfy

$$\#\mathcal{P} = \int_{\Omega} \frac{1}{R(\mathbf{y})^d} d\Omega. \tag{165}$$

We note that if we assume an analytical form of $R(\mathbf{y})$ then $\mathcal{P}$ that are far smaller then those obeying the above bound can be constructed. However, we only consider samplings that follow Supplementary Equation 165. Given this assumption, we can then ask if we can also bound $\frac{\#\mathcal{P}}{\#\mathcal{P}_c}$, where $\mathcal{P}_c$ is some sampling satisfying Supplementary Equation 165 for $R_c(\mathbf{y})$. If we consider the same worst case scenario as above we get,

$$\#\mathcal{P}_c \leq \#\mathcal{P} \int_{s(c_{\mathbf{i},l})} \frac{1}{(dist(\mathbf{y}, \mathbf{y}^*))^d} d\mathbf{y} \tag{166}$$

where $c_{\mathbf{i},l}$ and $\mathbf{y}^*$ are as above, and we note that the argument is independent of the exact level l, and so we consider the ratio of each individual particle cell to be the same giving the multiplication factor. In one and two dimensions this has closed form, in 1D we have

$$\begin{aligned} \#\mathcal{P}_c &\leq \#\mathcal{P} \int_3^4 \frac{1}{(y)^d} dy \\ &= \log(\frac{4}{3}) \#\mathcal{P} \end{aligned} \tag{167}$$

and in 2D

$$\begin{aligned}
\#\mathcal{P}_c &\leq \#\mathcal{P} \int_3^4 \int_3^4 \frac{1}{(\sqrt{x^2+y^2})^d} dydx \\
&= \#\mathcal{P}(-2G + \frac{1}{2}\pi \log(\frac{4}{3}) + i \left(\text{PolyLog}(2, \frac{-3i}{4}) - \text{PolyLog}(2, \frac{3i}{4}) \right)) \qquad (168)
\end{aligned}$$

where G is Catalan's constant and PolyLog is the PolyLogarithm. This corresponds to $\frac{\#\mathcal{P}}{\#\mathcal{P}_c}$ ratios being bounded by 3.47 in 1D, 24.3 in 2D, and using numerical integration in 3D 221.252. However, with the exception of possibly 1D, I know of no methods to realize these ratios. Further, there bounds are also not likely tight in practice, and I am unclear as to their utility.

Observed numerical bounds

To find more likely ratios of $\frac{\#\mathcal{P}}{\#\mathcal{P}_c}$ in practice, we numerically estimated the continuous optimal Resolution Functions using an $\mathcal{O}(N^2)$ brute force approach. The brute force approach relies on testing increasing sized $R(\mathbf{y})$ for each location. The first plot in Supplementary Figure 12 shows the ratio of R_c over R_b for the test function in Supplementary Figure 14 for decreasing E. We find that as suggested by the discussion above $\frac{R_c}{R_b} \to 1$ as $E \to 0$. Unfortunately, the solving for smaller values of E than 0.01 was too computationally costly.

To illustrate this high computational cost, in the second plot Supplementary Figure 12, we show a comparison in 3D between the brute force solution to the Resolution Bound and the Pulling Scheme for a fixed ratio benchmark. We find that the brute force solution takes between two and six orders of magnitude longer to compute. This corresponds to the brute force solution taking over 2 hours for an image of $N = 128^3$, compared to less than .01 seconds for the Pulling Scheme. We note that efforts were undertaken to optimize the brute force scheme and reduce the computational cost including acceleration using OpenMP to provide a 'fairer' comparison with the Pulling Scheme. This high cost placed a limit on the numerical analysis that could easily be done comparing the two solutions. Next we provide numerical results on the relationship between the implied Resolution Function R^* and the continuous estimate of R_b. We focus here on 3D. The first plot of Supplementary Figure 13, shows the mean ratio of $\frac{R_b}{R^*}$, averaged over all pixel locations against increasing number of objects for both noisy and noise-free original images. We find the average ratio is less than 3.2 across both benchmarks, with a decrease in the mean ratio for an increase in objects for the images. Therefore, on average we find the ratio is two to three times less than the worst-case bound of ≈ 6.93.

We use the estimate of R_b to also estimate the $\frac{\#\mathcal{P}}{\#\mathcal{P}_c}$ ratios. From the above analysis, we found a worst case bound of 221.25. In the second plot of Supplementary Figure 13 we show the estimated ratios against increasing information content for sampling based on both the isotropic and integral neighborhood sampling used in the performance benchmarks (Supplementary Note: 5). We find the ratio becomes constant for increasing information content, with the isotropic sampling having a ratio of approximately 11, and 5.5 for the integral neighborhood sampling. Although this is only for one test example, we find a ratio that is much less than the worst case bound given above.

First, we found that the bound between R_c and R_b in 1D was close to one for reasonable ranges of $E << .3$, and tends to one as $E \to 0$. In 3D using the later discussed implementation, we found the observed mean ratio of the implied and continuous resolution functions in 3D was between 2 and 3, depending on the image content and level of noise. Further, we find that the worst-case bounds for particle ratio $\frac{\#\mathcal{P}}{\#\mathcal{P}_c}$ are do not appear to be tight in practice, finding ratios of less than 11 in 3D benchmark examples (compared to the worst-case bound of 225.21).

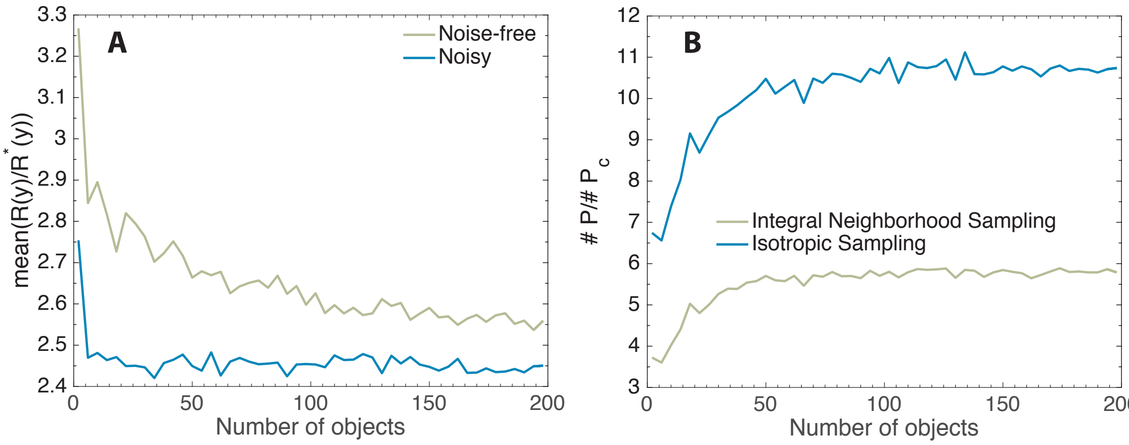

Supplementary Figure 13: **A** shows the mean ratio of the numerical estimate of the optimal continuous Resolution Function $R_b(\mathbf{y})$ over the implied Resolution Function $R^*(\mathbf{y})$ using the integral neighborhood sampling optimization in 3D against number of objects for both noisy and noise-free images. **B** shows the ratio of the number particles in the APR $\#\mathcal{P}$ divided by the theoretical sampling $\#\mathcal{P}_c$ based on R_c. We plot the ratios for both the isotropic and integral neighborhood sampling (Supplementary Note: 5) in 3D against the number of objects.

Supplementary Note 9: Generalized higher-order Reconstruction Conditions

In the previous derivations, we have placed conditions on the reconstruction of the function value everywhere. Here we show that the same procedure allows bounds on arbitrary derivatives of f.

So now we wish to calculate some high order derivative α, with order m derivative operators. This requires that f is $m + |\alpha|$ times differentiable. Here, we replace the classic Reconstruction Condition with the (α, m)-Reconstruction Condition, defined as

$$|\sum_{\mathbf{x}_p \in \mathcal{N}(\mathbf{y}, R(\mathbf{y}))} f_p \xi_{\alpha,p}(\mathbf{y}, \mathbf{x}_p) - \frac{\partial^{|\alpha|} f(\mathbf{x})}{\partial \mathbf{x}^{\alpha}}| \leq \sigma_\alpha(\mathbf{y}) E_{\alpha,m} \tag{169}$$

where α uses multi-index notation, to represent the desired derivative and $\xi_p^{\alpha}(\mathbf{y})$ is the derivative reconstruction kernel with convergence order m. Following the same steps as above, if we require the following conditions

$$\sum_{\mathbf{x}_p \in \mathcal{N}(\mathbf{y}, R(\mathbf{y}))} (\mathbf{x}_p - \mathbf{y})^{\mathbf{k}} \xi_{\alpha,p}(\mathbf{y}, \mathbf{x}_p) = \begin{cases} 1 & \text{if}, \mathbf{k} = \alpha \\ 0 & \text{elseif}, |\mathbf{k}| < m + |\alpha| \\ \text{bounded} & \text{otherwise} \end{cases}$$

then we have the following,

$$\epsilon_\alpha(\mathbf{y}) = \sum_{\mathbf{x}_p \in \mathcal{N}(\mathbf{y}, R(\mathbf{y}))} \sum_{|\mathbf{k}| = m + |\alpha|} (\mathbf{y} - \mathbf{x}_p)^{\mathbf{k}} \frac{|\mathbf{k}|}{\mathbf{k}!} \int_0^1 (1-t)^{|\mathbf{k}|-1} \frac{\partial^{|\mathbf{k}|}}{\partial \mathbf{x}^{\mathbf{k}}} f(\mathbf{y} + s(\mathbf{x}_p - \mathbf{y})) ds \xi_{\alpha,p}(\mathbf{y}, \mathbf{x}_p) \tag{170}$$

which we can bound by,

$$|\epsilon_\alpha(\mathbf{y})| \leq \gamma(m + |\alpha|) \max_{|\mathbf{k}| = m + |\alpha|} \max_{\mathbf{x} \in \mathcal{N}(\mathbf{y}, R(\mathbf{y}))} \frac{|\mathbf{k}|}{\mathbf{k}!} \left(\left| \frac{\partial^{|\mathbf{k}|} f(\mathbf{x})}{\partial \mathbf{x}^{\mathbf{k}}} \right| \right) R(\mathbf{y})^{m+|\alpha|} |\xi^{m+\alpha,p}| \tag{171}$$

where $|\xi^{m+\alpha,p}| = \sum_{\mathbf{x}_p \in \mathcal{N}(\mathbf{y},R(\mathbf{y}))} |\xi_{\alpha,p}(\mathbf{y},\mathbf{x}_p)|$. Now here, the coefficients, will be proportional to of $\frac{1}{R(y)^{|\alpha|}}$, hence we replace this with the following global bound of $|\xi^{m+\alpha,p}| \leq C_\alpha \frac{1}{R(y)^{|\alpha|}}$, where C_α is some constant that depends on the reconstruction function and local particle orientations, giving us now

$$|\epsilon_\alpha(\mathbf{y})| \leq \gamma(m+|\alpha|) \max_{|\mathbf{k}|=m+|\alpha|} \max_{\mathbf{x}\in\mathcal{N}(\mathbf{y},R(\mathbf{y}))} \frac{|\mathbf{k}|}{\mathbf{k}!} \left(\left| \frac{\partial^{|\mathbf{k}|} f(\mathbf{x})}{\partial \mathbf{x}^{\mathbf{k}}} \right| \right) R(\mathbf{y})^m C_\alpha. \tag{172}$$

Next, by making this reconstruction error satisfy our (α, m)-Reconstruction Condition Supplementary Equation 169 we get

$$R(\mathbf{y}) \leq \left(\frac{E_{\alpha,m}\sigma_\alpha(\mathbf{y})}{C^\alpha \gamma(m+|\alpha|) \max_{|\mathbf{k}|=m+|\alpha|} \frac{|\mathbf{k}|}{\mathbf{k}!} \max_{\mathbf{x}\in\mathcal{N}(\mathbf{y},R(\mathbf{y}))} |\frac{\partial^{|\mathbf{k}|} f(\mathbf{x})}{\partial \mathbf{x}^{\mathbf{k}}}|} \right)^{1/m}. \tag{173}$$

which if we again apply a smoothness assumption on σ_α making the usual substitution giving again

$$R(\mathbf{y}) \leq \min_{\mathbf{x}\in\mathcal{N}(\mathbf{y},R(\mathbf{y}))} (L_{\alpha,m}(\mathbf{x})) \tag{174}$$

where now

$$L_{\alpha,m}(\mathbf{y}) = \left(\frac{E_{\alpha,m}\sigma_\alpha(\mathbf{y})}{C^\alpha \gamma(m+|\alpha|) \max_{|\mathbf{k}|=m+|\alpha|} \frac{|\mathbf{k}|}{\mathbf{k}!} |\frac{\partial^{|\mathbf{k}|} f(\mathbf{y})}{\partial \mathbf{x}^{\mathbf{k}}}|} \right)^{1/m} \tag{175}$$

which is again in the correct form for using Particle Cells and the Pulling Scheme. We can see the condition in the previous section is simply the $\alpha = 0$ case.

Multiple resolution conditions

Above we have shown that we can formulate the Resolution Bound for a range of (α, m)-Reconstruction Conditions. What if we want more than one Reconstruction Condition? This case is simply satisfied. If we consider we have a set of Local Resolution Estimates $\mathbf{L}_i = \{L_{\alpha_i,m_i}(\mathbf{y})\}$ from $i = 1,..,q$ associated with q different (m, α)-Reconstruction Conditions (Supplementary Equation 169), then we get one Resolution Bound of the form,

$$R(\mathbf{y}) \leq \min_{\mathbf{x}\in\mathcal{N}(\mathbf{y},R(\mathbf{y}))} \left(\min_i L_{\alpha_i,m_i}(\mathbf{x}) \right) \tag{176}$$

using the fact that the minimum operation is associative. Hence, any combination of Reconstruction Conditions can be solved finding the Implied Resolution Function $R^*(y)$ using the Pulling Scheme using the minimum across the different (α,m)-Local Resolution Estimates. Hence, all of the results from the Particle Cells presented for the $m = 1$, $\alpha = 0$ case, directly extend without extra work to the multiple general (m, α)-Reconstruction Conditions case.

Supplementary Note 10: Image reconstruction methods

In the above discussions and main text, we have not specified a specific $\xi_p(\mathbf{y})$. We have only specified the two conditions any reconstruction must fulfill, specifically,

$$\sum_{\mathbf{x}_p \in \mathcal{N}(\mathbf{y},R(\mathbf{y}))} \xi_p(\mathbf{y}) = 1, \tag{177}$$

$$\xi_p(\mathbf{y}) \geq 0.$$

In essence, any average over points within the neighborhood $\mathcal{N}(\mathbf{y}, R(\mathbf{y}))$ is valid. Here we briefly describe three different approaches that are used in the following chapters, that produce reconstructions $\hat{f}$ that satisfy the Reconstruction Condition. Many other possible approaches exist, including using B-Splines or Wavelets for reconstruction, however, we stick to the following three simple cases here.

Piecewise constant reconstruction This first approach, is practically, the most simple and efficient. A piecewise constant reconstruction $\hat{f}_{pc}$ that satisfies Supplementary Equation 177 can be constructed as

$$\hat{f}_{pc}(\mathbf{y}) = \sum_{c_{\mathbf{i},l} \in \mathcal{V}} f_p \phi(\mathbf{y}, c_{\mathbf{i},l}) \tag{178}$$

where $\phi(\mathbf{y}, c_{\mathbf{i},l})$ is defined as in Supplementary Equation 37. Due to its simple structure, Supplementary Equation 178 can be very efficiently implemented and has low computational cost. Despite its simplicity, it seems to produces subjectively 'high' quality reconstructions. Because of these properties, we use it as the default reconstruction throughout the rest of this work. The draw back of this approach is not a 'smooth' reconstruction.

Smooth reconstruction

Instead, in the second approach, smooth reconstructions $\hat{f}_s$ can be used by utilizing a kernel function $\psi(\mathbf{x}) \geq 0$ in the following way

$$\hat{f}_s(\mathbf{y}) = \frac{\sum_{\mathbf{x}_p \in \mathcal{N}(\mathbf{y}, R(\mathbf{y}))} f_p \psi(\mathbf{x} - \mathbf{y})}{\sum_{\mathbf{x}_p \in \mathcal{N}(\mathbf{y}, R(\mathbf{y}))} \psi(\mathbf{x} - \mathbf{y})}. \tag{179}$$

Such smooth reconstructions could be useful for visualization purposes, when piecewise constant 'artifacts' may not be wanted, or for processing applications requiring a smooth representation.

A further smooth approach includes use of the piecewise construct reconstruction $\hat{f}_{pc}$. This can then be filtered consequentially in each direction using an average over each direction seperately with a filter size set by $R(\mathbf{y})$.

Worst-case reconstruction

For the analysis below, it is useful to be able to create the worst-possible reconstruction that satisfies Supplementary Equation 177, so we can show empirically that the Reconstruction Condition holds. If we consider any point $\mathbf{y} \in \Omega$, let $f_{min} = \min_{\mathbf{x} \in \mathcal{N}(\mathbf{y}, R(\mathbf{y}))}(f_p)$ and $f_{max} = \max_{\mathbf{x} \in \mathcal{N}(\mathbf{y}, R(\mathbf{y}))}(f_p)$, then any reconstruction satisfying Supplementary Equation 177 follows

$$f_{min} \leq \hat{f}(\mathbf{y}) \leq f_{max} \tag{180}$$

therefore, we define the minimum $\hat{f}_{min}$ and maximum $\hat{f}_{max}$, worst case reconstructions as

$$\hat{f}_{min}(\mathbf{y}) = \min_{\mathbf{x} \in \mathcal{N}(\mathbf{y}, R(\mathbf{y}))}(f_p), \tag{181}$$

and

$$\hat{f}_{max}(\mathbf{y}) = \max_{\mathbf{x} \in \mathcal{N}(\mathbf{y}, R(\mathbf{y}))}(f_p) \tag{182}$$

which represent upper and lower bounds on any reconstruction.

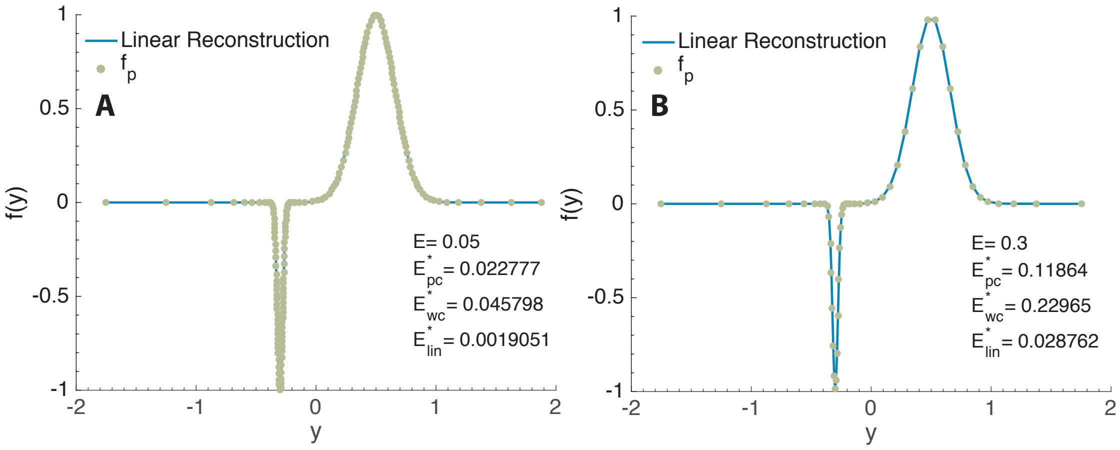

Supplementary Figure 14: The APR with $E = 0.05$ (**A**) and $E = 0.3$ (**B**) for $f(y) = e^{\frac{-(x-0.5)^2}{0.05}} - e^{\frac{-(x+0.3)^2}{0.001}}$ with $\sigma(y) = 1$ on the domain $\Omega = [-2, 2]$. The observed reconstruction errors (normalized infinity norm) are given inset for E^*_{pc} a piecewise constant reconstruciton, E^*_{wc} worst case reconstruction, and E^*_{lin} piecewise linear reconstruciton. For $E = 0.05$, $\#\mathcal{P}^* = 176$, and $l_{max} = 12$, for $E = 0.3$, $\#\mathcal{P}^* = 51$, and $l_{max} = 9$.

Supplementary Note 11: 1D Validation

In this supplementary note, we verify the theoretical results of the APR using a simple analytic 1D function. We use the most simple case where the Local Intensity Scale $\sigma(y)$ is a constant. First, we will briefly describe the algorithms that were used and then follow with a discussion of various results.

Implementation

The results in this section were produced using scripts in Matlab. The code takes a function f over a fixed domain Ω that can be queried at any point $y \in \Omega$. Given a user-set relative error E and the input function, the APR is then be computed.

The Pulling Scheme was implimented using the explicit tree storage method with a full representation of the Particle Cell set $\mathcal{C}$ from l_{min} to $l_{max} - 1$, where $\frac{\Omega}{2^{l_{max}}}$ represents the smallest distance between sampled particles. The equivalence optimization Supplementary Note: 5 was used. However, the integral neighborhood optimization was not used. l_{max} was set by finding the numerical maximum of the absolute value of the gradient, computed using central differences, and finding its associated particle cell level using $l = \max(l_{min}, \lfloor \log_2(\frac{\Omega}{L(y)}) \rfloor))$ and l_{min} was set to one. The natural Local Particle Set, $\mathcal{L}_n$ was then calculated by iterating over the domain at a sampling defined by $\frac{\Omega}{2^{l_{max}}}$. $\mathcal{L}_n$ was created by calculating $L(y)$ and then determining the associated Particle Cells setting and then setting the values in the $\mathcal{C}$ structure to one (For more details see the description of the 3D pipeline in Supplementary Note: 13).

We tested this pipeline, using a numeric, and symbolic version. In the symbolic version, all the function and gradient calls were symbolically evaluated. In the second, the numeric version, the function sampled at a spacing $\frac{\Omega}{2^{l_{max}}}$ was the input of the function. $L(y)$ was computed either using central differences for the numerical version. The pulling scheme was then used to calculate $\mathcal{V}_n$ and from this $\mathcal{V}$. Lastly, the particle set $\mathcal{P}^* = \{f_p\}$ was formed by sampling the function. As particle locations do not align with the sampling used in the previous steps when using the numerical computation, linearly interpolated values were used. Unless explicitly stated, all results are shown for the numerical version.

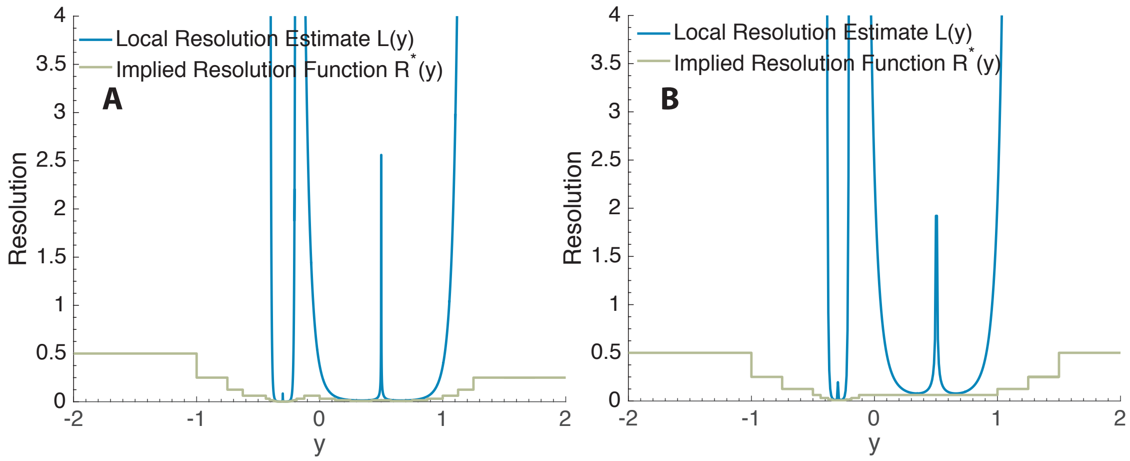

Supplementary Figure 15: Local Resolution Estimate $L(y)$ (blue) and Implied Resolution Function $R^*(y)$ (green) for the $E = 0.05$ (**A**) and $E = 0.3$ (**B**) examples from Supplementary Figure 14.

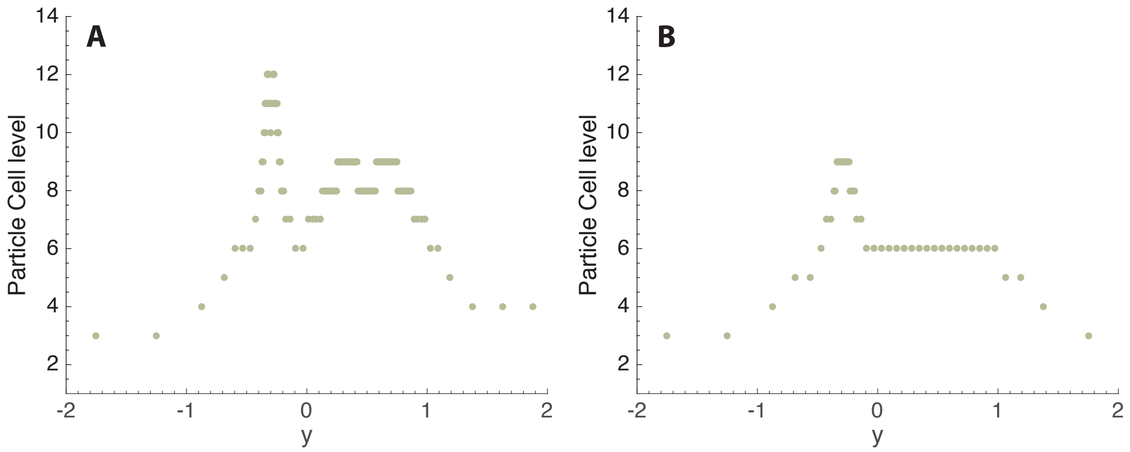

Supplementary Figure 16: Particle Cell level l, for all $c_{\mathbf{i},l} \in \mathcal{V}$ for the $E = 0.05$ (**A**) and $E = 0.3$ (**B**) examples from Supplementary Figure 14.

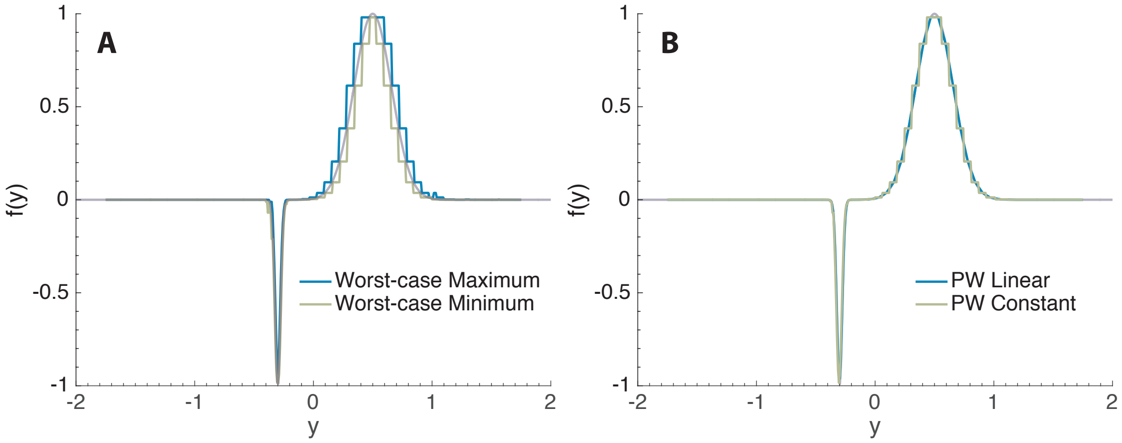

Supplementary Figure 17: Examples of three different reconstruction methods for the $E = 0.3$ APR from Supplementary Figure 14. **A** shows the maximum $\hat{f}_{max}$ (light blue) and minimum $\hat{f}_{min}$ (green) worst-case reconstructions (10) with the original function plotted in transparent blue. **B** shows piecewise constant interpolation $\hat{f}_{pc}$ (green) and piecewise linear inteprolation. The original function is plotted in transparent blue.

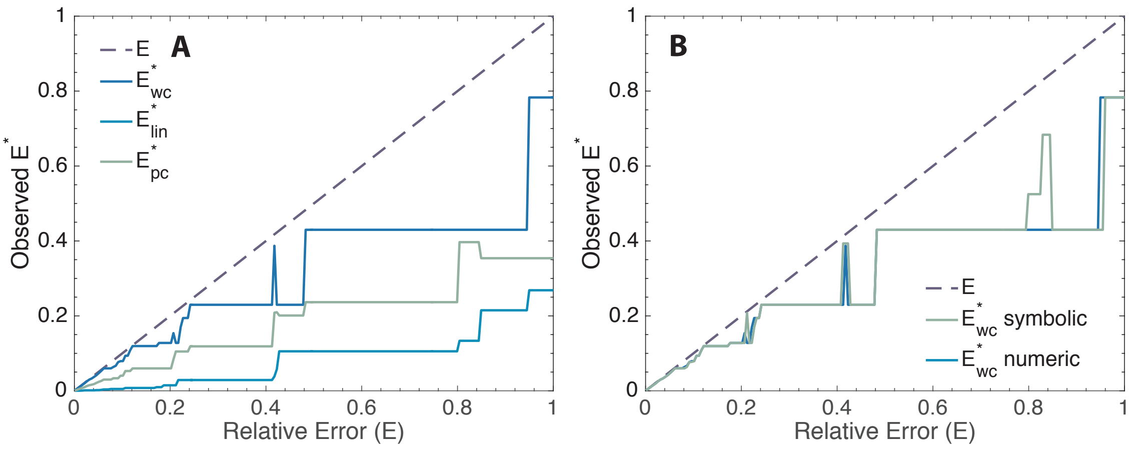

Supplementary Figure 18: The observed reconstruction errors for the APR and function as in Supplementary Note: 14 for a linear range of 200 values of relative error E from 0.001 to 1. In both plots the dotted dark blue line indicates $E^* = E$, the representing Reconstruction Condition that the APR reconstruction should be below. **A** shows the observed reconstruction errors for worst case E^*_{wc} (blue), piecewise linear E^*_{lin} (light blue) and piecewise constant E^*_{pc} (green). **B** shows the worst case reconstruction error E^*_{wc} when the gradient is computed analytically (green) and numerically using central differences (blue).

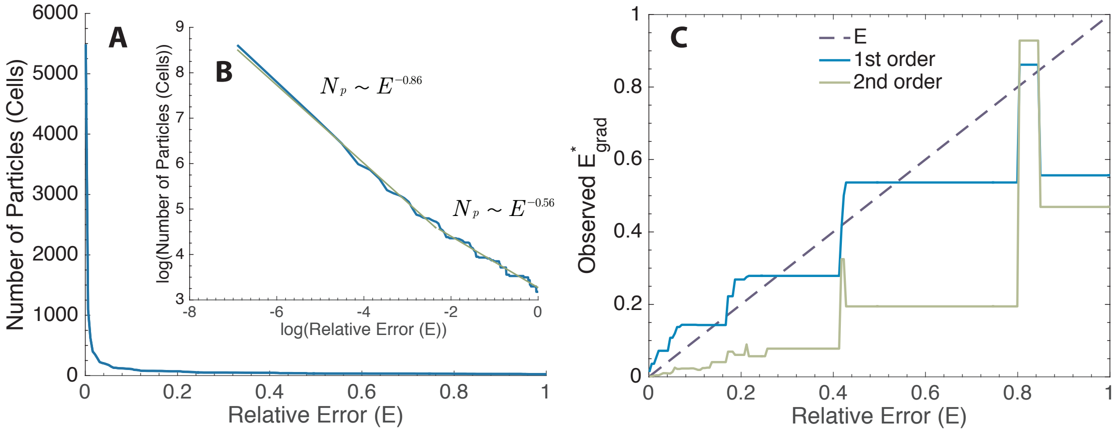

Supplementary Figure 19: **A** shows the number of particles ($N_p = \#\mathcal{V}$) for the APR and function as in Supplementary Figure 14 for a linear range of 200 values of relative error E from 0.001 to 1. **B** is the same data (blue) on a log-log plot, with two linear fits (green). The first fit is for $E \leq 0.05$, with exponent -0.86 and R-Square: 0.994 and second for $E > 0.05$ with exponent -0.56 and R-Square: 0.975. **C** shows the observed reconstruction error of the gradient computed on the same series of APRs. The observed reconstruction error of the gradient is the infinity norm of the gradient normalized by the maximum absolute value of the gradient. The dotted line shows the relative error bound, the light blue shows a first order gradient, and green second order gradient.

1D example

Here, we explore the APR for a simple 1D function for a function composed of a narrow negative and broad positive Gaussian function. Supplementary Figure 14, shows the APR represented as particles at f_p in green, and a piecewise linear interpolation in blue, for a high relative error $E = 0.05$ and low relative error $E = 0.3$ (Function definition in caption). We only use this example here; however, the results are consistent across general differentiable functions that have been tried. From the two plots, we can see that the particles are adapting to the different length scales in the problem, having a low density of particles in the flat areas and resolution increasing near the two peaks. Further, we can see that the impact of increasing the relative error is an increase in the resolution in the already higher resolution areas. In the inset, we show the observed reconstruction errors E^* of the two APRs for a range of different reconstruction methods. We define the observed reconstruction error E^* for a set of points $\bar{x}$ as

$$E^* = \max_{x \in \bar{x}} \left(\frac{|\hat{f}(x) - f(x)|}{\sigma(x)} \right) \tag{183}$$

where $\hat{f}$ is the reconstructed value from the APR. A subscript is usually given to indicate which reconstruction method was used, and $\bar{x}$ is the set of all points sampled at a spacing of $\frac{\Omega}{2^{l_{max}}}$.

For the 1D examples, we use three different constructions. E^*_{pc} is based on a piecewise constant nearest neighbor reconstruction $\hat{f}_{pc}$, E^*_{wc} is the worst-case taking the maximum reconstruction error for both $\hat{f}_{min}$ and $\hat{f}_{max}$ as described in Supplementary Note: 10, and E^*_{lin} is from a piecewise linear (between particles) reconstruction. Supplementary Figure 17 shows the reconstructions for the three cases for the APR with $E = 0.3$. For the case of $E = 0.05$ the reconstructions, except the worst-case, are indistinguishable by eye from the function. From theory, this observed reconstruction error should be less than or equal to E for all of these methods.

Returning to the values in Supplementary Figure 14, we see that this is the case. As expected, the worst-case reconstruction has the highest value, followed by the piecewise constant, and then piecewise linear reconstructions.

Next, we show details of the APR formation, and how the change of resolution between the two relative error values arises. The increase in E, from 0.3 to 0.05, results in a scaling of the Local Resolution function $L(y)$, shifting it to a smaller value. The lower values then result in a more constrictive Resolution Bound resulting then in a smaller Implied Resolution Function. This is shown in Supplementary Figure 15 where the Implied Resolution Function $R^*(y)$ (green) and the Local Resolution Estimate $L(y)$ (blue). However, across these figures, discerning the changes in resolution in high-resolution areas (small R^*) is difficult. However, this is easily done instead by directly visualizing the Particle Cell level l. Supplementary Figure 16, shows the changes in resolution by Particle Cell level l for the two different relative errors. We note that the Particle Cell level l for the higher relative error $E = 0.05$ seems to be more responsive to the features of the function then $E = 0.3$.

Reconstruction Condition

Above we showed that the Reconstruction Condition holds for two values of relative error E. What about the arbitrary E? To address this, we computed the APR and reconstruction errors E^* for 200 values from 0.001 to 1 for the three reconstruction methods. The results are plotted in **A** of Supplementary Figure 18. For small values of E the reconstruction errors show a linear response to E, and for higher values show a piecewise constant response. Across all values, the worst-case reconstruction, as predicted, is the highest. Further, although it comes close to the bound, represented by the dotted line, it never crosses it. These results, therefore, confirm that the Reconstruction Condition holds across E for our test function.

Numeric vs. symbolic gradient

The derivation of the APR assumes full knowledge of the gradient of the function $\frac{\partial f}{\partial x}$. In Supplementary Note: 7, we briefly discussed some theoretical arguments on how errors in the gradient would affect the observed reconstruction error E^*. To test the impact of this, we compared the worst-case reconstruction error of the APR computed with exact knowledge of f through symbolic evaluation of $\frac{\partial f}{\partial x}$ and the numeric version computed from knowledge of f only at samples of distance $\frac{\Omega}{2^{l_{max}}}$. All previous results have been with the numeric version. The result is shown in **B** of Supplementary Figure 18. For small values of E the results appear identical, however, for a few points at higher E, there are some differences. Indeed, the reconstruction error for the numeric code is smaller, except at isolated points for E near 1. Arguably, since the bound only requires E be below the bound, the lower value results from more particles being used, and therefore the higher analytical solution is 'better'. However, in this example, the difference between the two regarding the number of particles was small (1-2).

Number of particles

Intuitively, we should expect that the smaller E, the fewer particles that should be required to form the optimal solution to the Resolution Bound with Particle Cells, and therefore the number of particles should decrease with E. This is the case, and is shown in **A** of Supplementary Figure 19. The plot shows both the numeric and analytic version number of particles against the relative error E. Only one curve can be seen because the differences are indistinguishable when visualized this way. The plot shows that not only does the number of particles decrease with E, that it does so in a non-linear way. To explore this, in **B** of Supplementary Figure 19 we show the same results in a log-log plot. We find what appears to be two different regimes, corresponding

to linear regions in the log-log plot. In the figure, we also show linear fits for these two regions. For small values of E, the number of particles N_p, appears to scale like $E^{-0.86}$ and for higher values like $E^{-0.56}$.

Gradient

Satisfying the Reconstruction Condition, only guarantees the reconstruction of the function f at a specified relative error E, and does not bound the derivative. However, the error of the gradient should still scale with E. We empirically explore the gradients reconstruction error, defined as

$$E^*_{grad} = \max_{x \in \bar{x}} \left(\frac{|\frac{\hat{\partial f}}{\partial y}(x) - \frac{\partial f}{\partial y}(x)|}{\sigma_{grad}} \right) \tag{184}$$

where we set σ_{grad} to be equal to the maximum absolute value of the gradient across the interval. The normalization by the maximum absolute value of the gradient is to make the results comparable to the E bound for f. The results are shown in **C** of Supplementary Figure 19, where the gradient is computed using both 1st order, and 2nd order in h DC-PSE [6] derivative estimates. We find that in both cases as the error decreases in E. For the first order derivative, the error is above the bound set by E, however, for the higher order 2nd derivative, we see that the reconstruction error in the gradient is always below E.

Discontinuities

Lastly, for the 1D case, we explore the case where f is no longer in C^1 and contains discontinuities. We do this by adding two Heaviside step functions to the previously used example from Supplementary Figure 14. The existence of discontinuities violates the assumptions of the formulation of the APR. However, practically discontinuities can be handled when using the numerical version, given the introduction of a fixed maximum level l_{max} for the initial sampling. We do this by using sampling set by the previous example for the input $f\{\bar{x}\}$, but letting l_{max} for the APR be determined by the numerical computation of the derivative and $L(y)$. We show the resulting APR's for the same relative errors $E = 0.05$ and $E = 0.3$ in Supplementary Figure 20, with the observed reconstruction errors again inset. We find that the two piecewise constant reconstruction methods still satisfy the Reconstruction Condition, but the worst-case method does not. The piecewise reconstruction methods meeting the bound is the result of only computing E^* at the sampling points given by $\bar{x}$, which coincides with the highest sampling distance in the APR. Therefore, at the high-resolution regions, the reconstruction is simply the particle values f_p for these methods. However, the worst-case reconstruction effectively uses all points within $R^*(y)$. In this case, the reconstruction fails at the discontinuity. However, the same would occur for any discrete sampling across the discontinuity using an isotropic kernel with support greater than one point.

Supplementary Note 12: 2D wavelet comparison

APR implementation

The APR was implemented in 2D in Matlab following the same procedure described in 1D above. Where the particle intensities are estimated using all pixels in the Particle Cell.

Wavelet thresholding implementation

For comparison, we used `ddencmp` function in Matlab to produce the Haar wavelet transform of images and performed global, hard thresholding using `wdencmp` to perform wavelet thresholding. Where we present

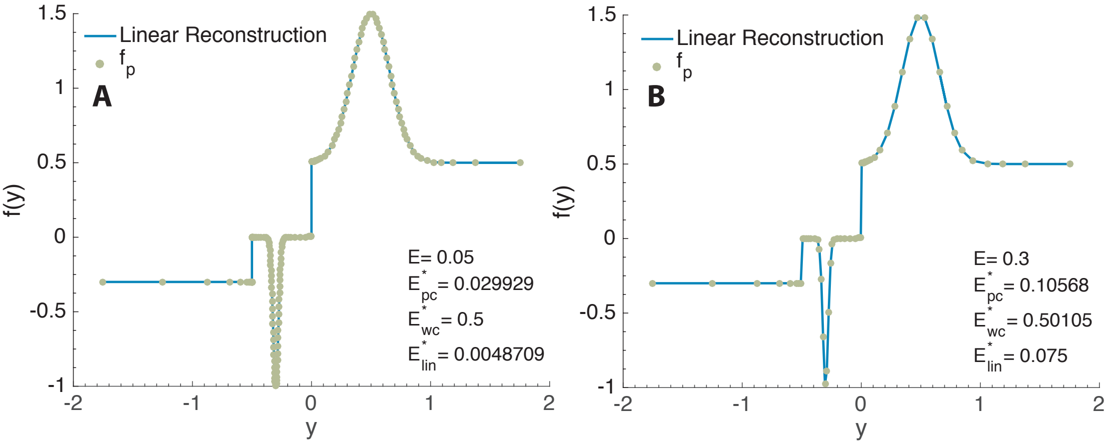

Supplementary Figure 20: The APR with $E = 0.05$ (**A**) and $E = 0.3$ (**B**) for $f(y) = e^{\frac{-(x-0.5)^2}{0.05}} - e^{\frac{-(x+0.3)^2}{0.001}} + 0.5*\text{Heaviside}(x) - 0.3 * \text{Heaviside}(-.5 - x)$ with $\sigma(y) = 1$ on the domain $\Omega = [-2, 2]$. The observed reconstruction errors (normalized infinity norm) are given inset for E^*_{pc} a piecewise constant reconstruciton, E^*_{wc} worst case reconstruction, and E^*_{lin} piecewise linear reconstruciton. For $E = 0.05$, $\#\mathcal{P}^* = 200$, and $l_{max} = 18$, for $E = 0.3$, $\#\mathcal{P}^* = 57$, and $l_{max} = 9$.

benchmarks similar to those produced in for wavelets in [7].

Error norm comparison

For both the APR and the Haar wavelet transform the number of coefficients required to represent the image (in the case of the APR, the number of particles) are decreased by increasing E for the APR and increasing the threshold used for wavelets.

The analysis was performed on a classic benchmark image (MIT cameraman shown in Supplementary Figure 22). For each generated wavelet transform of APR, the image was then reconstructed and the L_1 and L_2 errors compared to the original image. For the APR, piece-wise constant reconstruction was used.

The results for both images are presented in Supplementary Figure 22 in log-log plots. Across images, we see that the Haar wavelet thresholding provides a more optimal trade-off between the number of coefficients and reconstruction error measured both in terms of the L_1 and L_2 norm.

This is expected, as given the same power of two decompositions between the APR and the Haar wavelet transform, any APR could be represented using a Haar wavelet transform losslessly (with respect to APR particles), with a number of coefficients that is either equal to, or less than number of particles in the APR.

Further, the wavelet transform and thresholding relies on a fixed basis, or reconstruction function, whereas the APR can produce an image with guaranteed error bounds with a wide class of isotropic reconstruction functions across the domain. This flexibility comes at the cost of a larger number of coefficients for a given reconstruction error.

Supplementary Note 13: 3D florescence image APR pipeline implementation

In this supplementary section, we briefly outline how we have implemented the steps for forming the APR for noisy $3D$ fluorescent images. We make use of the optimizations for the integral neighborhood sampling

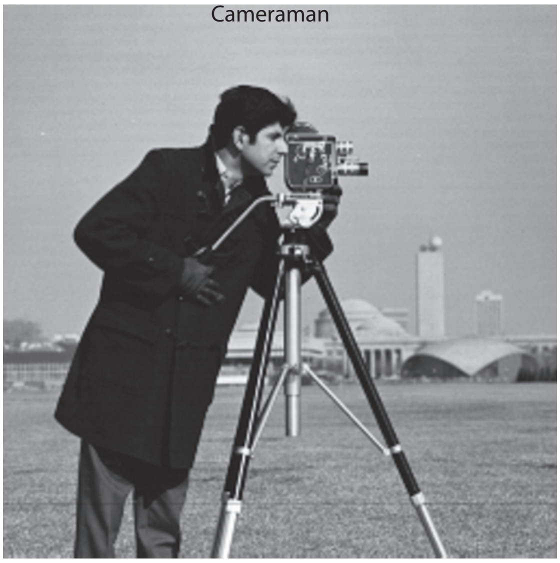

Supplementary Figure 21: Classic 512 $\times$ 512 benchmark image Cameraman (512 $\times$ 512 used for the optimal error scaling results in Supplementary Figure 22

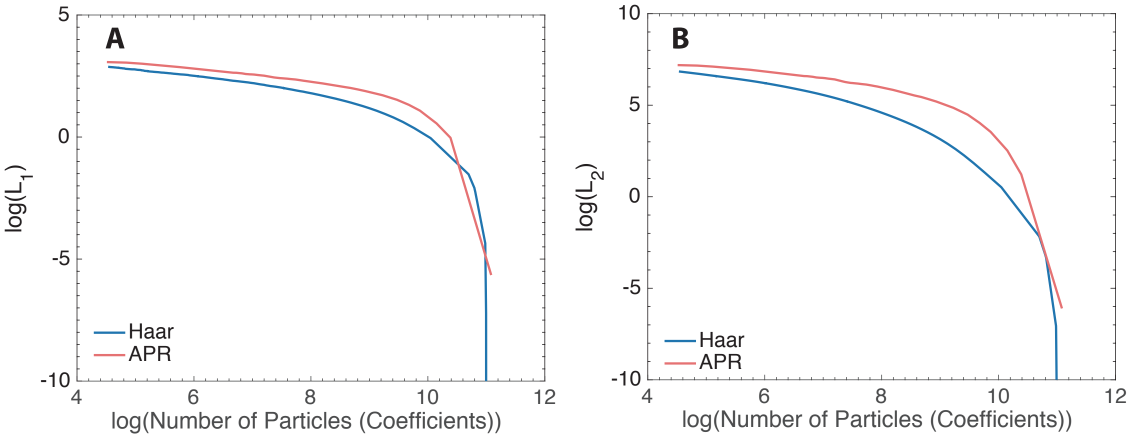

Supplementary Figure 22: The scaling results for the L_1 (**A**) and L_2 (**B**) errors plotted as a log-log plot against the number of non-zero coefficients in the thresholded Haar wavelet transform and the number of particles in the APR. The results are shown for the Cameraman benchmark image. For the APR the thresholds are reduced by increasing E, and for wavelets by increasing the threshold at which at which coefficients are set to zero. For the APR the piece-wise constant reconstruction was used.

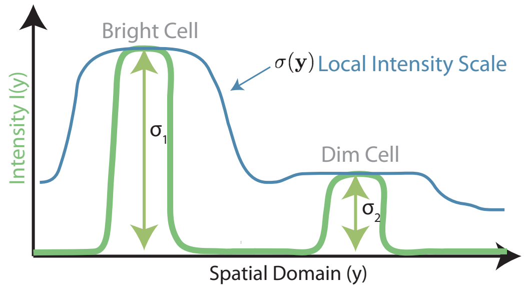

Supplementary Figure 23: The schematic shows the idea behind the Local Intensity Scale we use in this paper. The Local Intensity Scale should be slowly varying and reflect the range of intensities (from highest to lowest) of objects within a set length scale set by the width of the blur kernel in the image.

(Supplementary Note: 5) and equivalence optimization (Supplementary Note: 5). For the Pulling Scheme, we use explicit storage of $\mathcal{C}$ as described in Supplementary Note: 5. Note, in this section as we are now dealing with images, we will use I to represent the original noisy input image, instead of f as previously used.

We have implemented the pipeline in a C++ library utilizing shared memory parallelism across the pipeline using OpenMP [8]. We have favored the use of shared-memory parallelism over the use of GPU acceleration, due to the current larger availability of larger capacities of RAM when compared to GPU-memory allowing the processing of larger images on a shared-memory CPU implementation than one relying on fitting the whole pipeline into GPU memory. However, testing has indicated that the use of hardware acceleration, such as GPUs or Intel Xeon Phi's, can provide significant speedups for particular steps.

The main dependencies of the library, besides OpenMP, are for input and output of files. For reading images we use LibTiff, and for output, and writing the APR, we use HDF5 [9] and the HDF5 plugin for the BLOSC compression library [10]. Also, a Java wrapper has been created using SWIG.

When implementing the APR for 3D LSFM data, three main choices had to be made. First, how to calculate the gradient magnitude $|\nabla I(\mathbf{y})|$, second, what form of Local Intensity Scale $\sigma(\mathbf{y})$ to use and how to calculate it, and last, how to sample the image intensity at particle locations $I_p = I(\mathbf{y}_p)$. A summary of these steps is shown in Supplementary Figure 24. All decisions have been made with the objective of meeting the Representation Criteria through optimizing both robustness to noise and computational efficiency.

Pipeline input and memory requirements

All input images used in this paper are read in from 16 bit unsigned int single channel tiff images. The time taken to load the image is not accounted for in the timing benchmarks in this paper. The pipeline requires approximately $2\frac{5}{8}$ times the memory of the original image, and the maximum memory size is only restricted by the available RAM, and the global 64 bit unsigned integer access key.

Smoothing B-splines

To be able to estimate function gradients in the presence of noise we fit cubic smoothing B-splines as introduced in [11]. This introduces a regularisation smoothing parameter λ that determines how closely the fit splines must fit the original sample points. We implement these filters using the IIR approach described in

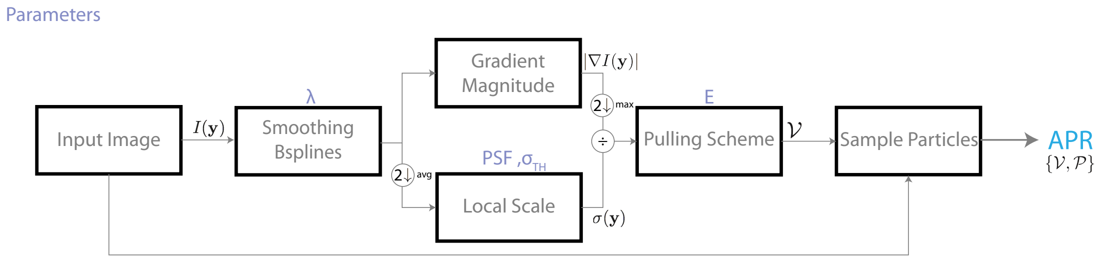

Supplementary Figure 24: Flow chart showing APR pipeline for fluorescent images, first smoothing B-splines are fit to the image, then the gradient magnitude $|\nabla f|$ and local scale $\sigma(\mathbf{y})$, is computed. The Local Resolution Estimate $L(\mathbf{y})$ is then computed and used to construct the input for the Pulling Scheme that then computes the OVPC set $\mathcal{V}$. The particles are then sampled from the original image, forming the APR. Required parameters are given above the boxes in purple.

[11], using the impulse response for setting the recursive boundary conditions. A strength of this approach is that the algorithm is $\mathcal{O}(1)$ regarding parameter choice λ, and $\mathcal{O}(N)$ in pixels, therefore displaying consistent computational performance across parameter values.

The output of the B-spline coefficients is used for gradient estimation and the Local Scale Function $\sigma(y)$.

Gradient magnitude $|\nabla I|$

Using the computed smoothing B-spline coefficients, the gradient in each direction is computed using the finite difference stencil $(-1/2h_i, 0, 1/2h_i)$. Where h_i is the sampling distance in that direction. These are then squared and combined to form the gradient magnitude as below

$$|\nabla I| = \sqrt{\left(\frac{\partial I}{\partial x}\right)^2 + \left(\frac{\partial I}{\partial z}\right)^2 + \left(\frac{\partial I}{\partial y}\right)^2}. \tag{185}$$

For noise-free benchmarks, simple finite differences on the original image are performed instead of smoothing B-Splines. Once calculated, the gradient magnitude is then downsampled by a factor of two in each direction, taking the maximum value over each patch of 8 pixels. This is done as the Local Resolution Estimate is only required at this resolution due to the equivalence optimization and to satisfy the smoothness assumption.

Local Intensity Scale $\sigma(\mathbf{y})$

In this paper we desire the Local Intensity Scale to be a smoothly varying function that captures the local range of the input image over a certain length scale, allowing the adaptation to cope with changes in contrast across the image domain with varying sources. We set this length scale as a function of the inherent length scale provided by the optical process through the Point Spread Function (PSF). This length scale is then used to adjust the size of the windows for the filters in the algorithm. The filter here is effectively a scaled smoothed estimate of the local standard deviation of the image with filter size adjusted to the estimated standard deviation of a Gaussian approximation to the PSF, PSF_w in each direction relative to the sampling size, and was inspired by [12]. In practice, the PSF is usually anisotropic in the z direction, this can be accounted for by an additional scaling parameter, but we do not explore this here. This parameter PSF_w in terms of pixels must be given by the user as input. As discussed below, we find the results relatively insensitive to the exact value of PSF_w. In an effort to satisfy the smoothness assumption (Supplementary

Note: 4) and given the Local Resolution Estimate $L(y)$ is calculated at spatial points downsampled by two, we directly calculate it on a downsampled by two smoothed B-spline image.

The first step of the Local Scale Function is two calculate

$$\sigma(\mathbf{y})^* = A_0\mu_2(|I_2(\mathbf{y}) - \mu_1(I_2(\mathbf{y}))|) \tag{186}$$

where A_0 is a scaling constant set by the filter window sizes, μ_1 and μ_2 represents box, or mean, filterers on the image with window sizes w_1 and w_2 respectively, and $I_2(\mathbf{y})$, is the smooth B-spline image downsampled by a factor of 2 in each direction using averaging. The box filter window sizes w in each direction and scaling factor has been empirically to minimize the under-resolution to $\mathcal{V}^{\text{ideal}}$ from the "perfect" APR discussed in Supplementary Note: 16 and set for integer values of the $s = \{1, 2, 3, 4, 5, 6\}$ in terms of pixels as

$$w_1 = \{1, 1, 1, 2, 2, 3\} \tag{187}$$

$$w_2 = \{2, 3, 4, 4, 5, 6\} \tag{188}$$

where non-integer values are rounded to the nearest value. The constant normalisation factor A_0 is required to normalize the estimate to that of the local intensities, and set empirically against the implied "ideal" σ^{ideal} for the calculated σ^* (again see Supplementary Note: 16) in Particle Cells at $l = l_{max}$ for each filter size combination as

$$A_0 = \{25.15, 37.70, 60.82, 30.24, 35.35, 20.47\}. \tag{189}$$

The result is thresholded in the following way

$$\sigma(\mathbf{y}) = \begin{cases} \max(\sigma^*(\mathbf{y})), \sigma_{th}) & \sigma^*(\mathbf{y}) > \frac{\sigma_{th}}{2} \\ 64000 & \textit{otherwise} \end{cases} \tag{190}$$

where σ_{th}, is set to the scale of the smallest content in the image that is wanted to be captured, e.g. the difference between the foreground and background of the dimmest object in the image. Otherwise, for noisy flat regions, as in image background, $\sigma \to 0$, resulting in the noise being captured (Supplementary Figure 25).

The box filters, are performed in a separable manner, in each direction using summed area tables [13], also known as integral images, to allow box filters computationally $\mathcal{O}(1)$ with respect to the window size.

For real exemplar data Supplementary Note: 17, an intensity threshold was also included, to allow the exclusion of unwanted dim image content in the background of the image or image camera defects. At this step any additional information, or filters, could be used to determine which part of the image wish to be captured, this could include information from different channels in an image, or from, different time-steps, such additions are use-case specific, but could yield a significant reduction in particle numbers.

Local Resolution Estimate $L(y)$ and Local Particle Set $\mathcal{L}$

The gradient magnitude $|\nabla I|$ and Local Scale Function $\sigma(\mathbf{y})$ are combined to create the Local Resolution Estimate $L(\mathbf{y}) = \frac{E\sigma(\mathbf{y})}{|\nabla I|}$ and then the Local Particle Cell set $\mathcal{L}_n$. When using the equivalence optimization Supplementary Note: 5, $L(\mathbf{y})$ is only required at locations that align with a down-sampled by two image (the maximum of the gradient is used over the patch at the original resolution).

The first steps requires setting the relation between the Particle Cells and the image domain. Given an image with N pixels, and image dimensions N_x,N_y and N_z, such that $N = N_x * N_y * N_z$, with maximum dimension N_{max}, and minimum dimension N_{min}. We can then set the minimum and maximum levels l for the APR as $l_{max} = ceil(\log_2 N_{max})$ and $l_{min} = \max(2, l_{max} - \lfloor \log_2 N_{min} \rfloor)$, and then our augmented domain length $|\Omega^*| = 2^{l_{max}} h_{min}$, set such that the maximum resolution coincides with the original pixel sampling.

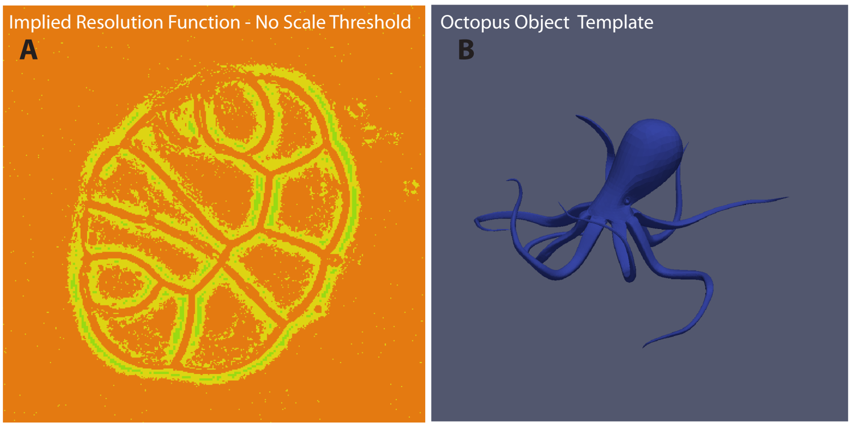

Supplementary Figure 25: **A** The Resolution Function $R^*(\mathbf{y})$ for the same image shown in Figure 3E, without the use of any thresholds for local scale estimation ($\sigma_{th} = 0$). The color scale is identical to that shown in Figure 3E. In the absence of a threshold, the Local Intensity Scale tends to zero in the background areas, and therefore fully resolves the background noise. This is mitigated by using a minimum local scale threshold σ_{th}. The original data is from exemplar data set 10 in Supplementary Table 3. **B** shows a volume rendering used for the octopus template used to generate the object function for synthetic images in Supplementary Figure 29

Then for each downsampled pixel value we calculate, the level l, of the Particle Cell $c_{\mathbf{i},l}$ it belongs to as

$$l = \max(l_{min}, \lfloor \log_2(\frac{|\Omega^*|}{L(y)}) \rfloor) \tag{191}$$

The spatial co-ordinates $\mathbf{i}$ of the particle can be calculated as $\mathbf{i} = \{\lfloor x\frac{2^l}{|\Omega^*|}\rfloor, \lfloor y\frac{2^l}{|\Omega^*|}\rfloor, \lfloor z\frac{2^l}{|\Omega^*|}\rfloor\}$. We wish to find all the unique $c_{\mathbf{i},l}$ that then form $\mathcal{L}$.

Instead of directly computing the coordinates, we make use of the relationship between the levels of the tree structure and parent-child Particle Cell relationships. Each full level l of the Particle Cells corresponds to a down-sampled version of the full image a fixed number of times, forming a classic image pyramid. To calculate $\mathcal{L}_n$ the image containing l for each downsampled pixel is downsampled to l_{min} using the max operation. For all values where the level in the structure, coincides with the down-sampled level the Particle Cell is added to $\mathcal{L}_n$ where the coordinates in the downsampled image x, y, z also coincide with the Particle Cell spatial coordinates and with l define a unique Particle Cell $c_{\{x_l, y_l, z_l\},l}$. Although this down-sampling results in missing $c_{\mathbf{i},l}$ that have higher level children in $\mathcal{L}_n$ however we know these Particle Cells do not impact $\mathcal{V}$ from the redundancy property (Supplementary Note: 5). Because the implimentation we use here also uses the same pyramid structure, instead of outputting the set $\mathcal{L}_n$, we simply set all values for which l is the current level of the pyramid to 1 and all other 0 and use this as input to the Pulling Scheme. This algorithm allows the construction of this reduced $\mathcal{L}_n$ in $\mathcal{O}(N)$.

Pulling Scheme $\mathcal{V}$

Given the Local Particle Cell set, $\mathcal{L}_n$ stored in an image pyramid with non-zeros indicated the present Particle Cells calculated from the previous step, we then run the Pulling Scheme using the full explicit storage of the Particle Cell tree as described in Supplementary Note: 5 using Algorithm 1 in Supplementary Note: 5. Practically, this is achieved by $(l_{max} - 1 - l_{min})$ unsigned 8 bit arrays of the the size 2^{3l} (See Supplementary Note: 5 for the memory cost in terms of N.).

The output of the algorithm is also stored in an image pyramid structure, with non-zero values aligning with SEED, FILLER, and BOUNDARY values representing the Particle Cells in $\mathcal{V}_n$.

Now that the valid Particle Cell set, $\mathcal{V}_n$, is calculated we then choose our particle sampling. This is done, as described in Supplementary Note: 5, where Particle Cells of type boundary and filler, have one particle placed in the center, and Particle Cells of type seed, are split into 8, higher resolution Particle Cells with one particle again at the center of each. The highest resolution particles coincide with the original image sampling, resulting in the construction of the particle sampling $\mathcal{P}^*$.

Intensity estimation I_p

Any method of estimation of the particle intensities $I_p = I(\mathbf{x}_p)$, could be utilized at this step. In the case of noise-free images, the closest, or interpolated pixel value is used. However, in the presence of noise, the use of information from $\mathcal{V}$, to improve the estimate of the intensity using an area of the original image would be more appropriate with $I_p = \hat{I}(\mathbf{x}_p)$, where $\hat{I}$ is some de-noised image. Here, given each particle is sampled at the center of the particle cell, we simply take the average of the intensity over all pixels within the Particle Cell. In the case of particles at the image resolution, this average would simply be the original pixel.

Reconstruction methods

For the comparison of the APR with images, a reconstruction method must be used. In Supplementary Note: 10 we discussed the reconstruction methods used in this section. Unless otherwise explicitly mentioned, it should be assumed that the piecewise constant reconstruction method (Supplementary Note: 10) is used. This was chosen for its computational efficiency, simplicity, and effectiveness.

Pipeline parameters

A summary of the algorithmic steps required to form the APR from an input image $I\{\bar{\mathbf{y}}\}$ are shown in Supplementary Figure 24. The parameters that must be set are shown in purple. These are the smoothing parameter λ for gradient estimation, the threshold parameters for the Local Intensity Scale σ_{TH}, the point speed function width (standard deviation of guassian approximation) PSF_w, and the desired relative error E. A detailed discussion of all parameters, their interpretation, and how they have been and can be, set is given in Supplementary Note: 14 below. For all of the benchmarks the parameters that have been used are described in Supplementary Note: 16.

In this paper, we have discussed and implemented one particular implementation of a pipeline for the APR optimised for large $3D$ images originally stored as tiffs. We have attempted to optimise the steps to reduce memory overhead and computation time, and to reduce the number of parameters and library dependencies. However, with the exception of requiring an estimation of $L(y)$, and an implementation of a pulling scheme, we imagine the possibility of use of vastly different algorithms, implementations and definitions of the estimation of the local scale function, gradient magnitude, and particle intensity.

Supplementary Note 14: Pipeline parameter summary

The required parameters for the APR pipeline presented here can be grouped into two categories, those that reflect information on the properties of the original image, and those that impact how and what the APR represents in the image.

Image parameters

The main parameters is the standard deviation of a Gaussian approximation to the point spread function (PSF) in each direction in pixels, PSF_w. This value is used to set the width of window functions used in calculating the Local Intensity Scale. We find the results are relatively insensitive to the exact value, and for Exemplar data, have used a fixed width of 2 pixels.

Reconstruction parameters

These parameters impact how the APR is formed from the underlying image and includes the relative error bound E, the gradient smoothing parameter λ, and the minimum local scale threshold σ_{th}.

The relative error bound E, determines the allowed distance between the original image, and the APR representation. From noisy synthetic data a value in the range of $0.08 - 0.15$ seems optimal in terms of PSNR for a range of noise levels that coincide to typical fluorescence imaging. We have found this reflected in qualitative experience with real data-sets as found in the exemplar data sets. Further, we found that for highly anisotropically sampled datasets, a value in the lower range of $0.08 - .1$ was usually appropriate, likely compensating for the resolution loss in one direction, where as for more isotropic data sets, higher values in the range of $0.1 - 0.15$ seemed optimal. However, in all cases, the results are insensitive to the exact value.

The gradient smoothing parameter λ controls the how much smoothing is done in the fitting of B-splines for local resolution function estimation. With a higher value resulting in greater smoothing. This smoothing is required due to the amplification of noise properties of standard gradient operators in the presence of noise [11]. The absence of this smoothing would result in erroneous high-gradients and over-sampling. How to set this parameter depends on the signal to noise ratio of the original image. With values ranging from 0.5 to 4, seemingly optimal over standard signal to noise ranges, with lower values for higher signal to noise ratios. Again, results are not especially sensitive to this result, with a value too low, resulting in over-sampling and likely fitting of noise, and a value set too high, resulting in the APR not adapting to the fine grain structure.

Lastly, the minimum Local Intensity Scale threshold σ_{th} represents the minimum local scale that will be allowed across the image domain. This is required due to the behavior of the Local Intensity Scale $\sigma(\mathbf{y})$. In flat regions, the response tends towards the average noise range. Hence, given the gradient will be non-zero in these regions it will result in the fitting of the noise by the APR, due to the normalization of the small gradients as shown in the first pane of Figure 25. σ_{th} is introduced to curb this effect. To reduce the impact of noise, the smoothed B-spline image is used as input to the local-scale function rather than the original. This results in a reduced response of flat background areas in the Local Intensity Scale, allowing for the threshold to still function at low signal to noise ratios. Further to a minimum bound, values at half this value are then set to a maximum response. This is in effect to drive the $L(\mathbf{y})$ to large values and result in the APR ignoring these regions.

Setting this value is therefore subjective, and image dependent, as real-images often contain dim signals from sample contamination or auto-fluorescence, that may or may not wish to be captured. Corresponding to b in our image formation model (194). If the faintest objects that wish to be captured can be identified in the image, the value can simply be set to the local range between background and foreground for this object. Unlike the other parameters, the setting of this parameter too high can result in significant changes in the properties of the APR, due to it resulting in signal effectively being ignored. Therefore, a conservative under-estimate is suggested. For the exemplar benchmarks, this was set by simple visual inspection of the original image. Alternatively, given assumptions on the minimum signal to noise ratio, this value can be estimated using an estimate of the background noise level. We found setting the value to 6 times the average background noise level is effective across the Exemplar datasets. However, this can still result in the inclusion of auto-fluorescence or background content, that may wish to be removed by manual selection of the parameter.

An image intensity threshold can also be used, where the gradient is set to zero where the intensity is

below some level I_{th}. This was not used in the benchmark data but has proved useful when dealing with real data, for removing unwanted background signal. Similarly, additional information can be included in the APR formation, most simply this can be done using a binary mask. Providing a simple means for inclusion of information from other channels, prior knowledge, or extra image processing steps.

Supplementary Note 15: 3D synthetic data

To test the properties of the APR for 3D Lightsheet Flouresence Microscope (LSFM) data we use synthetically generated image data. We generate synthetic images following a Object function and image formation model we describe below. Synthetic data is used as it allows us to control image parameters, such as image size, content, and noise level in addition to full knowledge of the noise-free, ground-truth image and Object function. We provide an overview and some technical details below.

Object function

Here we model LSFM data as a set of labeled objects, e.g. cell nuclei, distributed in space that are the input to an image formation process that produces images. We define our Object function (ignoring time) defined on $\Omega \subset \mathbb{R}^3$ with M objects as

$$O(x, y, z) = \sum_{i=1}^{M} O_i(x, y, z) \tag{192}$$

where each object is a function of compact support $O_i(x, y, z)$ and the function is set to zero outside of Ω for simplicity. Further, we allow object is composed as

$$O_i(x, y, z) = B_i O^*(x - x_i, y - y_i, z - z_i) \tag{193}$$

where $O^*(x, y, z)$ is a piecewise constant function of compact support, that we call the template object, and B_i is a constant we call the brightness of object i. In all but one case, the template object used below is a sphere of varying size.

Image formation

Given a particular Object funtion, we form an image $I\{\mathbf{y}\}$, approximating the image formation process. The first step involves the simplified version of the image formation process [14] and discrete approximation of

$$\begin{aligned} I^*(x, y, z) = \iiint_\Omega &(O(u, v, w)+ \\ &b)PSF(x - u, y - v, z - w)dudvdw \end{aligned} \tag{194}$$

where b represents the background intensity is set to be a constant and PSF as set as a non-spatially varying Gaussian with a standard deviation in each direction of PSF_w. For efficiency, the convolution is only done once over the template object, allowing a high sampling approximation to the Object function, without explicitly storing it. The ground truth image is then formed by integrating over the pixel (voxel) volume (h_x, h_z, h_y) to create the pixel intensity for each location as

$$I_{gt}\{x, y, z\} = \int_{x-h_x/2}^{x+h_x/2} \int_{y-h_y/2}^{y+h_y/2} \int_{z-h_z/2}^{z+h_z/2} I^*(u, v, w)dudvdw \tag{195}$$

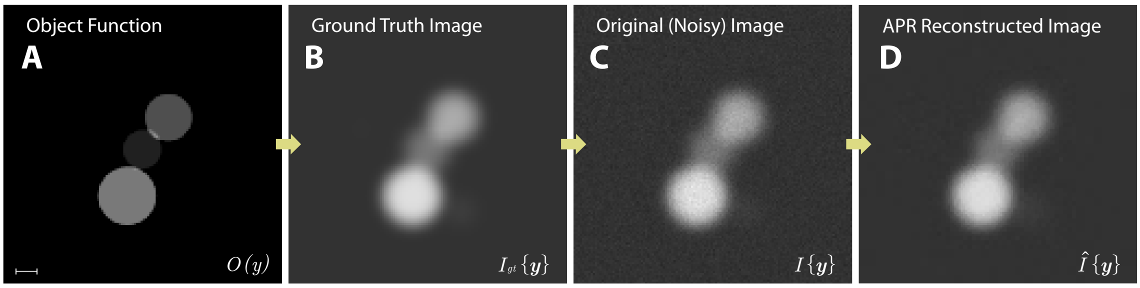

Supplementary Figure 26: Flow chart showing the generation of synthetic images used for benchmarking the APR. First template objects are generated of a certain size, given locations (x_i, y_i, z_i) , and brightness B_i, to define the Object function $O(\mathbf{y})$ (**A**). The Object function is then blurred through convolution with a Gaussian kernel PSF, and then sampled to produce the Ground Truth Image $I_{gt}\{\mathbf{y}\}$ (**B**). This ground truth image is then corrupted by a Gaussian approximation to Poisson Noise η, to generate the Original Image $I\{\mathbf{y}\}$ (**C**). This original image is then transformed into an APR. The APR can be then used to produce a reconstructed image $\hat{I}\{\mathbf{y}\}$ that can be compared with both the original and ground truth image for benchmarking (**D**). Scale bar indicates 10 pixels.

for fixed locations $\bar{\mathbf{y}}$, the spacing of pixels and pixel volumes does not need to be the same (isotropic). However, this is the case for the benchmarks here. Note, we have also integrated of z dimension as a simplification. Again a discrete approximation to the integral is used. The last step involves the corruption of the image by noise as

$$I\{x, y, z\} = I_{gt}\{x, y, z\} + \eta(x, y, z, I_{gt}\{x, y, z\}) \tag{196}$$

where $\eta(x, y, z, I_{gt}\{x, y, z\}) \sim \mathcal{N}(I_{gt}\{x, y, z\}, I_{gt}\{x, y, z\})$ a Gaussians noise with mean and variance equal to the intensity of the pixel as an approximation to Poisson noise [15]. In fluorescence imaging, the image can be corrupted by multiple different noise sources with different properties including components that have spatial structure [16]. However, here we only consider the case of Poisson, or shot, noise arising from statistical quantum fluctuations by using a Gaussian approximation. The image $I\{x, y, z\}$ at locataions $\hat{\mathbf{y}}$ we denote as $I\{\hat{\mathbf{y}}\}$, it is this image that is transformed into the APR.

Step Summary

Supplementary Figure 26 provides an example of 2D slices of the steps in the synthetic image generation pipeline. Throughout the benchmarks below, we alter the synthetic images regarding image size, information content, quality, and sampling. We will briefly describe how this is done for each, relating to the parameters mentioned above. Figure 3C, provides examples of what the original image of a fixed sphere template looks like under different conditions.

The image size can be changed by setting the appropriate size of the domain Ω, pixel locations $\mathbf{y}$ and pixel size h_x, h_y, h_z.

Given our Object function model of the image, we can define the level of information content to be proportional to the number of objects M in the image. Therefore, we can scale the image content for a given size image and sampling, by increasing the number of objects M. The objects are given random locations uniformly distributed across the domain and often have a random uniform distribution of brightness B_i, within the range B_{min} and B_{max}. An example is shown in the **G** in Figure 2.

We alter the degree of image blur and its shape using the width of the Gaussian kernel and its standard deviation parameters PSF_w. Here, we show results for three levels of blur; we call small, medium, and large blur. They correspond to a standard deviation in terms of pixels of 1, 3, and 6 respectively.

Here we consider either noise-free, that is η is set to zero, or the noisy case using a Poisson noise approximation. The image quality can be then altered, by changing the relative magnitude of the η compared to the object brightnesses B_i. The mean of η within any object i can be approximated by a combination of $b + B_i$. Therefore, we can increase or decrease the image quality by increasing or decreasing the ratio of $\frac{B_i}{B_i+b}$. This is done by keeping a fixed average object brightness B_i and then changing the background b. Hence, we are altering the average Peak Signal to Noise Ratio (PSNR) of the image. We show results here for three levels of image quality we call low, medium and high noise level. Figure 2D, gives examples for these levels of image noise.

Lastly, the degree of sampling can be changed. This involves decreasing the pixel size h_x, h_y, h_z and sampling $\mathbf{y}$ while keeping all other variables fixed in real variables. Practically, this means the blur width PSF_w defined in pixels has to be appropriately increased. The increase in sampling can be thought of as zooming in on the object, as with a camera lens.

Implementation Details

The synthetic image generation pipeline is implemented separately in a C++ library we have named SynImageGen, this code available from the authors and uses the ArrayFire [17] Library for GPU acceleration for generating the images. The use of the GPU limits the size of generated images to GPU memory, only allowing images up to 1000^3 to be generated. In fact, the generation of synthetic images usually accounted for the largest component of benchmarking. The pipeline is designed such that the parameters describing each synthetic image are sufficient to recreate any of the images in the pipeline. Below we will describe additional technical details of the synthetic image generation for producing the different image steps in Supplementary Figure 26.

The template images used here, are piecewise constant images, with objects of various intensity, size, and location placed within a fixed $3D$ image size. The process begins with the generation of a binary object template. This template is then used for multiple instances of the same object within the image domain. Objects templates can be generated by simple algorithms as is the case with the used sphere template. Alternatively, templates can be generated by a binarization of 3D polygon model files (vrml, obj) using binvox [18, 19]. This is the case with the more complicated octopus benchmark shown in Supplementary Figure 25 and used for results shown in Supplementary Figure 29 which was downloaded from [20]. However, as mentioned, for all other benchmarks provided here the generated sphere was used here due to its computational efficiency, and simplicity.

Objects were placed using a uniform random distribution within the volume, as not to overlap with the boundary, this is to reduce the impact of choice of boundary conditions for the pipeline on the results. Object intensities were set again with a uniform random distribution with a minimum and maximum intensity value set.

The ground truth image is then generated by convolving the image with a blur kernel and adding a fixed background intensity. The convolution was done using separable filtering using 1D Gaussians of set sigma PSF_w in each direction. This blurred image, then served as the ground truth, as it represents the fluoresce distribution that we wish would observe if it was not corrupted by noise.

The last step of the synthetic pipeline is the corruption of the image by noise. For each pixel, the following noise process is used and drawn from

$$\hat{I}(\mathbf{y}) \sim N(I(\mathbf{y}), I(\mathbf{y})) \tag{197}$$

where $N(I(\mathbf{y}), I(\mathbf{y}))$ is the normal distribution with mean and variance equal to the image intensity.

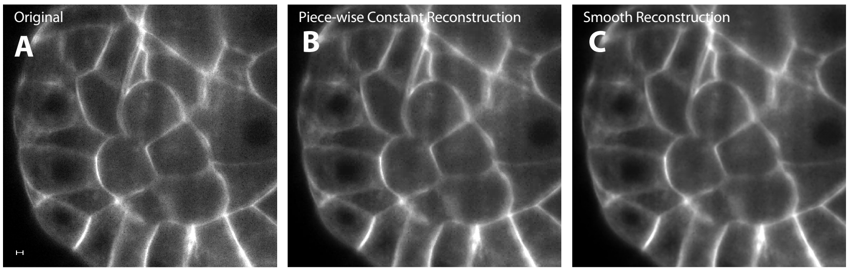

Supplementary Figure 27: Comparison between original image (**A**) and APR piece-wise constant Reconstruction (**B**) and APR smooth reconstruction (**C**). The smooth reconstruction is using all points within the integral interaction neighbourhood $/n$ and normalized Guassian weighting based on distance. Source: (Dataset number 10 Supplementary Table 3). The scale indicates 10 pixels.

Supplementary Note 16: Validation benchmark details

In this section, we give details of the synthetic benchmarks used to evaluate the properties of the APR with the results presented in the Validation section in the main text. For each data point in the benchmarks, a synthetic image is generated and used as input to produce an APR that is then reconstructed using the appropriate method (usually piecewise constant reconstruction) and summary and image statistics are calculated. This analysis is then saved in an HDF5 file that is then read, analyzed and plotted using Matlab. All scripts and data for the production of the plots in this paper are available on request. Further, to aid reproducibility, each file contains the git hash for the code commit used to produce the results (from LibAPR), the command line input parameters, and an exhaustive list of parameters used to generate the analysis. The parameters for the synthetic image generation are either as stated explicitly below, or as outlined in 14.

Benchmark parameter selection

See 14 for a detailed discussion of parameters, and their use. For the synthetic datasets, all image parameters are taken as known from the image generation process including the blur sizes. For the reconstruction, parameters are set in the as described below, unless explicitly varied as a parameter for the benchmark.

Default parameter set to $E = 0.1$, unless otherwise stated.

The minimum Local Intensity Scale threshold σ_{th} was set to the minimum bound for the random distribution of template intensities. This is a limitation of the results here and objectively should be set instead by an automated method. However, there is a large range of values over which the results are insensitive for this parameter.

Gradient smoothing parameter λ was set in an automated fashion, we utilized the fact that we knew the minimum standard deviation of the noise σ_{noise} of the benchmark image, being the $\sqrt{I_b}$, where I_b is the constant background intensity. We then ran parameter searches across different noise levels σ_{noise}, and parameter values λ, to find the minimum λ required to be still able to get three levels of resolution change with an object with brightness above the background set at the minimum local scale threshold σ_{th}. We then used the symbolic curve fitting toolbox Eureqa [21] to fit the value λ given the input variables give us

$$\lambda = (\frac{\sigma_{th}}{\sigma_{noise}} 0.498763)^{\frac{-1}{0.6161}} \tag{198}$$

which was used in the synthetic benchmarks, providing good results in both low and high PSNR benchmarks.

In all cases with error bars have been given, they reflect the estimate of the standard error.

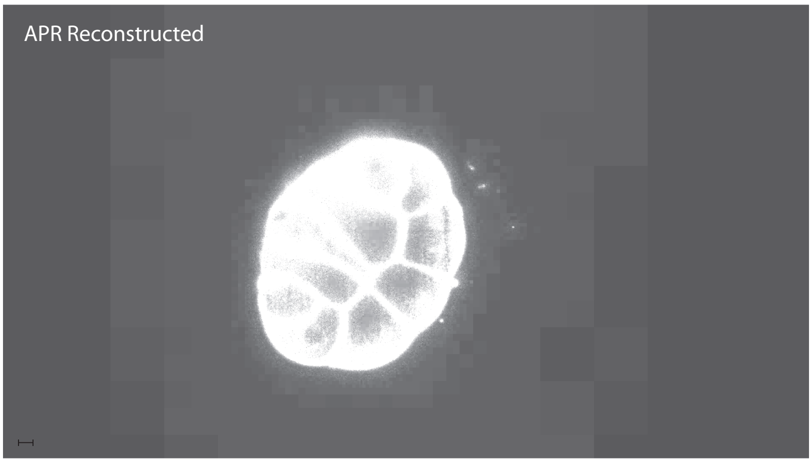

Supplementary Figure 28: Piecewise constant reconstructed APR shown in Figure 3E, with contrast adjusted to show the lossy nature of the APR in the background regions. (Dataset number 10 Supplementary Table 3). The scale bar indicates 40 pixels

Noise-free image Reconstruction Condition

Parameter values for results presented in Figure 3. Images of fixed size and number of objects are generated, and the required relative error bound E is varied. The Reconstruction Condition requires that the observed reconstruction error E^* is below E for all locations. Where the observed reconstruction error is defined as in Supplementary Equation 183.

An image size of 128^3 was used with five sphere templates randomly placed in the domain with brightness B_i varying uniformly between 500 and 5000 with a background intensity $b = 1000$. The blur kernels used have standard deviations of $PSF_w = \{1, 3, 6\}$, in pixels, corresponding to the low, medium and high blur, with isotropic sampling as $h_x = h_z = h_y = 0.1$. The minimum local scale threshold $\sigma_{th} = 500$. Due to the lack of noise, finite differences were used to approximate derivatives instead of smoothing B-splines. The relative error bound E, was run in two linear sections with 40 samples, from $0.001 - 0.1$, then from $0.1 - 1.0$, with 40 repeats for each relative error bound.

Noisy image Reconstruction Condition

The same benchmark as above was repeated but with the introduction of Poisson noise with results shown in Supplementary Figure 30.

The image parameters and settings were set as in the no-noise case above.

The average observed relative error for the noisy image was constructed by taking the average of the infinity norms of the individual original images.

Also in Supplementary Figure 30 data was presented for the image quality, measured by the Peak Signal to Noise Ratio (PSNR) and how it, varies with E, for noisy images. We do this for original images with different initial image quality (PSNR), by varying the signal to noise ratio. Further, on the right axis, a comparison between the reconstruction error from the APR, and the noise level in the original image is given as measured by the Mean Squared Error (MSE).

Different noise level images were created by fixing the background intensity $I_b = 1000$, and varying the brightness of the sphere templates, giving an estimated SNR of $\frac{\sigma_{noise}}{I_{obj}}$, where I_{obj}, is the intensity of

the original object template. Due to the Poisson noise corruption, the effective standard deviation of the noise level will be at-least $\sigma_{noise} = \sqrt{I_b} = \sqrt{1000}$. Therefore, we run the benchmark with 3 different object intensities $I_{obj} = \sqrt{1000}, 10\sqrt{1000}, 30\sqrt{1000}$, corresponding to the high, medium low noise images respectively.

An image size of 128^3, was used with 5 sphere templates randomly placed in the domain, with intensities and background set as discussed above. The medium blur kernel ($PSF_w = 3$) was used and isotropic sampling with $h_x = h_z = h_y = 0.1$. The minimum local scale threshold σ_{th}, was set to the object intensity set for the original image. The relative error bound E, was run in two linear sections with 40 samples, from $0.001 - 0.1$, then from $0.1 - 0.4$, with 10 repeats for each relative error bound.

In the benchmark, we compare two different methods of estimation of the particle intensities from the noisy input. In both cases, for Particle Cells with a larger resolution then pixel resolution an average of particles in the spatial domain of the Particle Cell is used. For those Particle Cells at pixel resolution in Supplementary Figure 30, we compare results for using the original pixel value or instead using a median filtered value. For all other results in this section, we use a median filtered value.

Also, in the second plot of Supplementary Figure 30, we also compare the results for the APR where the Particle Cells are computed from the noisy input, but the particle intensity values are computed from a noise-free image.

To measure image quality we use both the Peak Signal to Noise Ratio (PSNR), calculated as

$$PSNR = 10 \log_{10}(\frac{64000}{MSE}) \tag{199}$$

where MSE is the mean squared error and is calculated as

$$MSE = \frac{1}{N^*} \sum_{\mathbf{y} \in \hat{\Omega}} (I^*(\mathbf{y}) - \bar{I}(\mathbf{y}))^2 \tag{200}$$

where I^* is the ground truth image, $\bar{I}$ is the image being compared (either the original image, or reconstructed image).

Increasing information content

In this benchmark we assess how well the APR is adapting to the image content. This is done by increasing the number of objects in the image and comparing both the image quality and the number of particles, with results given for the same low, medium, high levels of image quality as for the reconstruction image quality benchmark.

The number of sphere templates randomly placed in the image was increased from $1 - 100$ in steps of 4, with 5 repetitions.

An image size of 300^3, was used, with a blur kernel between the low and medium used ($PSF_w = 2$) and isotropic sampling with $h_x = h_z = h_y = 0.1$. With the object intensity and background set as for the noisy image reconstruction benchmark. The relative reconstruction error was set to $E = 0.1$.

The ratio of the PSNR for the APR reconstructed image, PSNR(APR), and the PSNR for the original image PSNR(Original) is given, showing the relative image quality of the reconstruction to the original image. Computed as described for the reconstruction image quality benchmark.

To measure image quality we use the PSNR*, calculated as

$$PSNR^* = 10 \log_{10}(\frac{64000}{MSE^*}) \tag{201}$$

where MSE* is the mean squared error and is calculated as

$$MSE^* = \frac{1}{N^*} \sum_{\mathbf{y}\in\hat{\Omega}} (I^*(\mathbf{y}) - \bar{I}(\mathbf{y}))^2 \tag{202}$$

where I^* is the ground truth image, $\bar{I}$ is the image being compared (either the original image, or reconstructed image), and $\hat{\Omega}$ is the those pixels in the domain for which the local scale function is less then 60000. This effectively excludes the calculation of statistics of background areas in the image due to the action of the minimum local scale threshold σ_{th}.

Increasing image size

In this evaluation benchmark we assess the impact of the original image size, by holding the number of objects fixed, and increasing the image dimensions.

The benchmark was run at three different levels of information content, using 10, 50 and 200 sphere objects placed randomly within the image domain. For each level of information, the image size was increased from 50^3 to 1000^3 in steps of 50, with 5 repetitions.

A blur kernel with $PSF_w = 2$ was used and isotropic sampling with $h_x = h_z = h_y = 0.1$. The template intensity and σ_{th} were set as described from the medium PSNR original image.

Increase sampling

In this benchmark with results shown in Supplementary Figure 31, the sphere template object was held constant and in a fixed position in the center of the image. The sampling resolution was then increased while keeping all other variables fixed in real terms. Relative error was set to $E = 0.12$, and the $PSF_w = 10.75$. The sampling h_i ranged from a minimum of 0.027 to a maximum value of 0.3583 that corresponded to 200 different image sizes ranging from 50^3 to 650^3. The smoothing parameter $\lambda = 20\left(\frac{h_i}{50}\right)^2$ was heuristically set.

Evaluation of Local Intensity Scale

Given the proposed Local Intensity Scale σ, for our implementation is only heuristically motivated. It is of interest, therefore what the ideal Local Intensity Scale σ^{ideal} would be. Using σ^{ideal}, we could then test the appropriateness and performance of our choice. Although we have no closed form of the ideal Local Intensity Scale, we can instead use synthetic data to numerical compute a perfect APR and infer the σ^{ideal} from this. This perfect APR is constructed for a given synthetic image by generating an identical image with no-noise and with all objects of the same object brightness, i.e. $B_i = 1$ (see Supplementary Equation 193) and calculating its gradient magnitude $|\nabla I|^{\text{normalized}}$. Then σ^{ideal} is defined as,

$$\sigma^{\text{ideal}}(\mathbf{y}) = \begin{cases} \frac{|\nabla I(\mathbf{y})|}{|\nabla I(\mathbf{y})|^{\text{normalized}}} & |\nabla I(\mathbf{y})|^{\text{normalized}} \geq .001 \\ 64000 & \text{otherwise} \end{cases} \tag{203}$$

where $|\nabla I|$ is the gradient magnitude of the noise-free image. The perfect APR can then be constructed using $|\nabla I|$ and σ^{ideal} for the calculation of the "ideal" OVPC $\mathcal{V}^{\text{ideal}}$ and hence $R^{*\text{ideal}}(\mathbf{y})$. Sampling of intensities can then be done from the original noisy image to isolate the impact of any errors in adaptation.

To explore the limitations of the Local Intensity Scale σ used in our 3D pipeline, we use the perfect APR. For each benchmark the APR can then be generated using the pipeline, and then compared to the perfect APR, which is calculated using exact knowledge of both the gradients and proposed ground truth Local

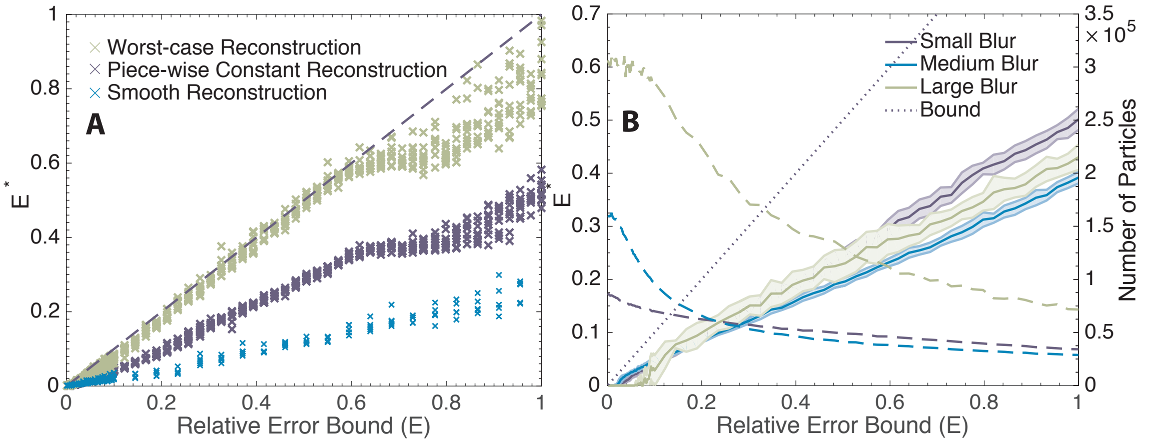

Supplementary Figure 29: **A** shows the medium level of blur benchmark, now showing the observed reconstruction error for three different reconstruction methods. The worst-case (green), piecewise constant (purple) and smooth reconstruction (blue) methods (See Supplementary Note: 10). Each cross represents an individual APR and Image comparison. **B** is a repetition of noise-free benchmark in Figure 3 with octopus template image (see Supplementary Figure 25) used instead of the sphere template for the Object function.

Intensity Scale σ^{ideal}. The perfect APR approach provides the ideal OVPC set $\mathcal{V}$, but then samples particles from the noisy original image. Therefore, we can then evaluate how well the used σ performs in different scenarios. Further reconstructions from the perfect APR, give a limit on the reconstruction accuracy under perfect adaptation.

For the benchmark image size of 80^3 was used with three different levels of objects $\{1, 10, 20\}$ (sphere with size = 20 pixels), and low, medium, and high image noise and blur set as in benchmarks above (Supplementary Note: 16 & Supplementary Note: 16). Each set of parameters was repeated 10 times and the results presented are the arithmetic mean. The Relative Error was set to $E = 0.1$. Each objects brightness B_i varied randomly over an order of magnitude.

For each image, the perfect APR, and APR are calculated, both sampling from noisy intensities. For each image, the ratio of the number of particles (Particle Cells) in the two representations is calculated $\frac{N_p}{N_p^{perefect}}$. Where N_p is the number of particles in the normal APR, and $N_p^{perfect}$ the perfect APR.

To evaluate reconstruction accuracy, the ratio of the PSNR of different image reconstructions from the APR where compared to the PSNR of the original noisy input image (where PSNR has been calculated as defined in Supplementary Note: 16). $\frac{PSNR(I_{pc})}{PSNR(I_{original})}$ is the PSNR of the piecewise constant reconstructed APR image (Supplementary Note: 10) divided by the PSNR of the original normal image. $\frac{PSNR(I_{pc}^{perfect})}{PSNR(I_{original})}$ instead is the ratio of the PSNR of the piecewise constant reconstructed perfect APR image divided by the PSNR of the original normal image. $\frac{PSNR(I_{smooth})}{PSNR(I_{original})}$ is the PSNR of the linear smooth reconstructed APR image (Supplementary Note: 10) divided by the PSNR of the original normal image. E_{pc}^{*} represents the observed reconstruction error for the APR using piecewise constant reconstruction.

Given the two different OVPC sets $\mathcal{V}^{ideal}$ and $\mathcal{V}$ we can compare the difference in particle cell resolution between the two. δ_+ and δ_- represent the ratio of Particle Cells in $\mathcal{V}$ that are of higher (+), or lower (-), level then the ideal case. That is by how much in different regions is the APR over, or under, resolving the image. Lastly, $\frac{\sigma}{\sigma^{ideal}}$ gives the mean ratio of the calculated σ and the ground truth ideal σ^{ideal} calculated over the Particle Cells at the highest level (l_{max})

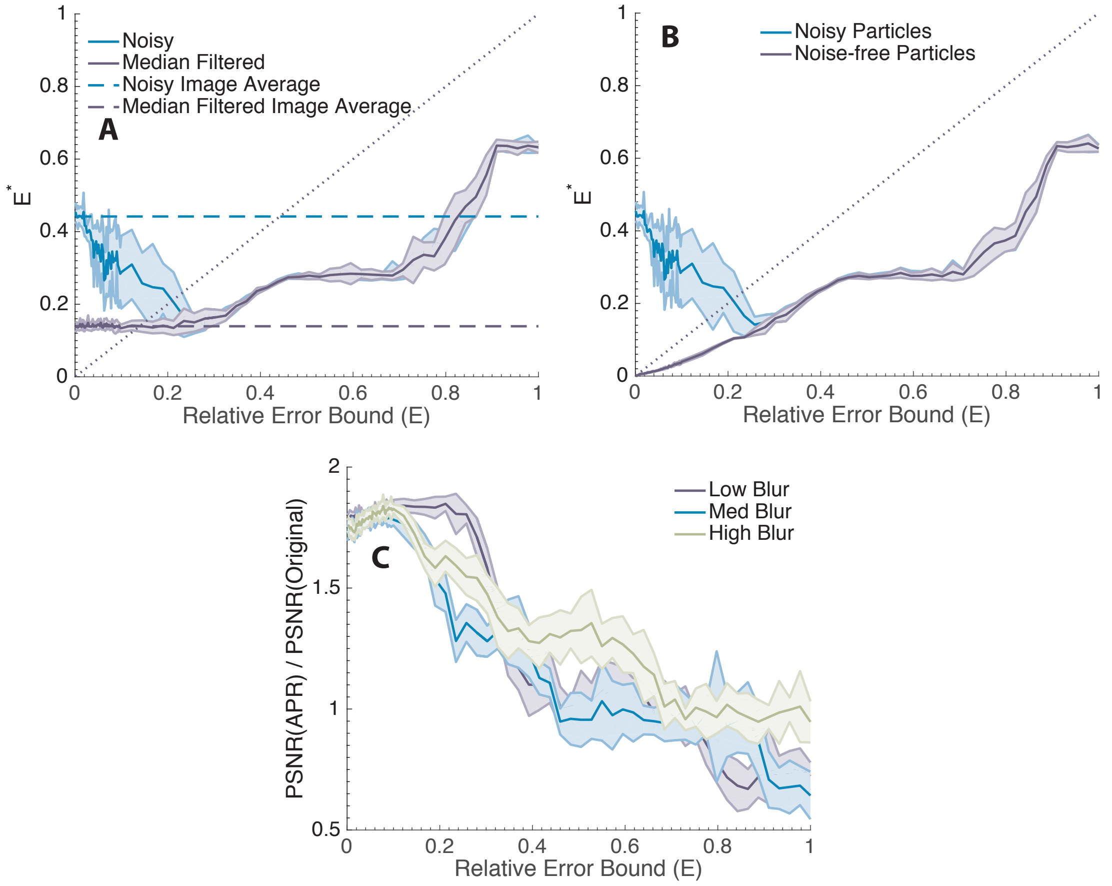

Supplementary Figure 30: **A** shows the observed reconstruction error E^* for piecewise constant reconstruction for a noisy image when for particles at pixel resolution the original noisy pixel values are used (blue) or a median filtered pixel value in purple (Medium image noise and blur). The dashed lines show the mean observed reconstruction error E^* of the original image (blue) and median filtered image (purple). The Reconstruction Condition $E = E^*$ given by dark blue dotted line. **B** compares the E^*, for the piece-wise constant reconstruction in the first plot (blue line) with particle values computed from noisy pixel values, with E^* of the same APR's computed from the same particles, with the intensities instead estimated from noise-free pixel values (purple). **C** shows the ratio of the PSNR of the APR for piece-wise constant interpolation (median filtered pixels used) and the PSNR of the original image against increasing set Relative Error Bound E for a medium image noise level and changing blur.

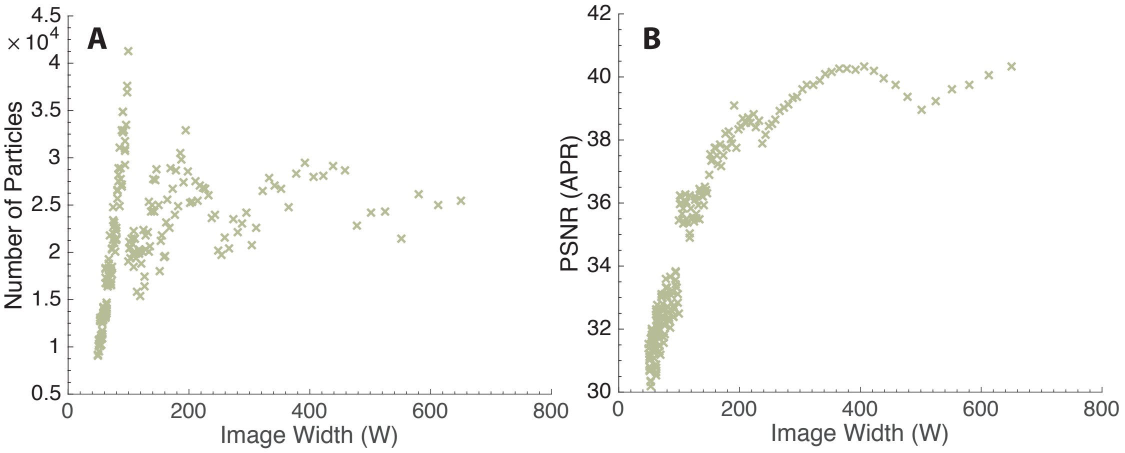

Supplementary Figure 31: **A** shows the number particles N_p, and **B** the PSNR of the APR for an image with fixed image content and blur size, but increased sampling, and hence width W. The benchmark is equivalent to choosing the resolution of a natural image for a fixed scene. (See Supplementary Note: 16 for parameters)

A small image size was chosen (80^3), as at 20 objects, the confinement results in 'effective' objects that can have brightness levels that vary from $\approx$ 1000 to $\approx$ 60000, almost two orders of magnitude. This is the result of objects being placed randomly and in smaller domains over-lapping between objects being common. (Beyond this level of objects the observed intensities consistently were above 64000, hence not being outside the scope of our u16bit pipeline used here).

In Supplementary Table 1 we find that across image noise level and the number of objects the reconstruction quality using the piecewise constant reconstruction of the APR is greater than the original image, except for low noise images with 20 objects. In contrast, for the perfect APR, it is always higher. However, for the APR using the piece-wise linear, smooth reconstruction we find the PSNR is again always higher. Regarding the number of particles, we find that for high noise images the APR uses more particles on average, whereas for medium and low noise images this depends on the number of objects. This coincides, with the values of δ_+ and δ_- indicating that for lower quality images the APR is over-resolving the image and for the 20 objects of medium and low noise level under-resolving. Therefore, it seems the APR is performing well, with exceptions of the 20 object case.

To further explore the behavior in Supplementary Table 2 we show additional results for the 20 object case showing the impact of the level of image blur. Here we can see that the performance is impacted by the level of image blur. With the medium and high blur scenarios resulting in the under-resolution of the solution. Interestingly, we find that in this case where the reconstruction quality is lower, the Reconstruction Condition, reflected by E^* has been met, as all values are below 0.1. This indicates that it is the Local Intensity Scale that is likely the cause of the reduction in quality when compared to the perfect reconstruction. This is further backed up by the comparison of ideal Local Intensity Scale given by $\frac{\sigma}{\sigma^{ideal}}$, being high in situations across all cases of 20 objects, and worst for high blur images. We note that in the high noise images, the errors occurred by any under-sampling are within the noise-level and therefore to not largely impact the results. It is only in the high image quality regime, where the errors in under-resolution result in a loss in image quality when measured by PSNR using piecewise construction.

In conclusion, these results indicate that the simple σ used here, although effective in many cases, is in-effective in crowded environments with large intensity fluctuations. Therefore, more sophisticated Local

Intensity Scales, would likely be ideal. One approach that would seem well suited, would be application specific learning of the functional form using deep learning [22].

Image Quality	Objects	$\frac{N_p}{N_p^{perfect}}$	$\frac{PSNR(I_{pc})}{PSNR(I_{original})}$	$\frac{PSNR(I_{pc}^{perfect})}{PSNR(I_{original})}$	$\frac{PSNR(I_{smooth})}{PSNR(I_{original})}$	$E_{pc}^{*}(E=.1)$	$\frac{\sigma}{\sigma^{ideal}}$	δ_-	δ_+
Low									
	1	3.571	1.557	1.843	1.599	7.634	1.023	0.002	0.627
	10	2.095	1.298	1.443	1.323	6.438	2.549	0.006	0.392
	20	1.719	1.255	1.357	1.278	6.552	3.516	0.021	0.296
Med									
	1	1.623	1.666	1.736	1.715	0.327	1.192	0.002	0.228
	10	1.028	1.307	1.330	1.354	0.239	2.531	0.075	0.080
	20	0.950	1.191	1.234	1.249	0.257	3.580	0.123	0.059
High									
	1	1.476	1.590	1.558	1.641	0.085	1.202	0.003	0.180
	10	0.947	1.124	1.192	1.221	0.067	2.447	0.072	0.041
	20	0.869	0.984	1.128	1.114	0.075	3.584	0.131	0.028

Supplementary Table 1: Evaluation of the Local Intensity Scale σ using the perfect APR benchmark

Image Quality	Image Blur	$\frac{N_p}{N_p^{perfect}}$	$\frac{PSNR(I_{pc})}{PSNR(I_{original})}$	$\frac{PSNR(I_{pc}^{perfect})}{PSNR(I_{original})}$	$\frac{PSNR(I_{smooth})}{PSNR(I_{original})}$	$E_{pc}^{*}(E=.1)$	$\frac{\sigma}{\sigma^{ideal}}$	δ_-	δ_+
Low									
	Low	2.753	1.103	1.335	1.112	13.387	1.982	0.002	0.532
	Med	1.439	1.260	1.338	1.285	4.637	2.052	0.016	0.271
	High	0.965	1.403	1.397	1.435	1.631	6.515	0.046	0.085
Med									
	Low	1.185	1.204	1.236	1.227	0.403	2.309	0.008	0.106
	Med	1.017	1.179	1.213	1.237	0.291	1.903	0.046	0.052
	High	0.647	1.189	1.252	1.282	0.077	6.527	0.314	0.020
High									
	Low	0.999	1.143	1.161	1.155	0.088	2.279	0.021	0.018
	Med	0.970	0.919	1.075	1.087	0.098	1.988	0.065	0.038
	High	0.639	0.889	1.148	1.101	0.039	6.483	0.306	0.027

Supplementary Table 2: Evaluation of the Local Intensity Scale σ, using the perfect APR benchmark

Supplementary Note 17: Benchmark data

In this section, we give additional technical details regarding the CR and exemplar benchmark data.

Computational Ratio (CR) benchmark data

To assess the performance of the APR for different levels of image content, we choose three different CRs for our synthetic benchmark data and then vary the original image size N. To represent low, medium, and high image content relative to image size, we generate data sets for varying image size N and number of objects M that approximately correspond to CRs of 100, 20, and 5 respectively. In Supplementary Figure 32, we show the maximum projection for examples of a CR5, CR20, and CR100 synthetic data sets with image size $N = 400^3$ (We use this CR'X' notation in figures and the remaining text). However, we could not determine a procedure for generating a precise CR for a given image. Instead, the datasets were generated using a linear

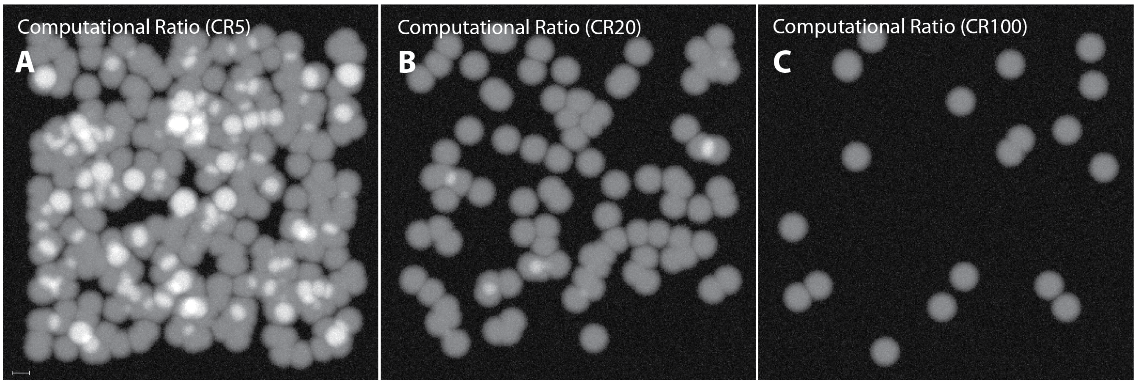

Supplementary Figure 32: Maximum intensity projection of examples of the Computational Ratio (CR) benchmark data that are used to represent **A** high (CR5), **B** medium (CR20) and **C** low (CR100) levels of information content ($N = 400^3$). The scale bar indicates 20 pixels.

estimate of the number of objects required to reach a certain ratio (see below). Generated in this way, the CR does vary across N, and the average CR values for $N = 200^3$ to $N = 1000^3$ are 5.8, 19.3, and 89.4 for the CR5, CR20, and CR100 cases respectively. The values of CR were set as to span realistic values as seen in the Exemplar benchmark data.

To achieve a certain CR, the number of objects must be changed with the image size N. The number of objects in the benchmarks was set as $\frac{N}{33400CR}$, that was determined empirically. The actual CR will not exactly be the ratio, but the above formula was found to provide good results for images of a width greater than 200. The images were isotropically sampled, and medium blur and medium PSNR setting were used as described in the benchmark evaluation section. The relative error bound $E = 0.1$, the gradient smoothing parameter $\lambda = 3.098$, as set by the automated scheme.

Exemplar datasets

To assess the performance of the APR on real datasets, 19 fluorescent microscopy datasets were also benchmarked. The datasets are across a range of image sizes, labels, specimen, and microscopes. A summary of the datasets, their properties, are given in Supplementary Table 3 and the parameters used in Supplementary Table 4 to create the APR. The CRs for the exemplar data sets range from a minimum of 5.6 to a maximum of 180, with a mean of 42.1 and median of 28.5. Parameters were set by experience and inspection of the original image. As mentioned in the discussion of parameters (Supplementary Note: 14), the parameter that can most greatly alter the result is the setting of the minimum local scale threshold σ_{th}, and the intensity threshold I_p. In all cases, parameters were set conservatively, including all content we could consider relevant to try and give lower bounds on the CR. In particular, the presence of auto-fluorescence influences this decision, and given the ability of the user to discriminate auto-fluorescents in many cases significantly higher computational ratios could be achieved.

Supplementary Note 18: Data structures

Given the Optimal Valid Particle Cell set $\mathcal{V}$ and particles $\mathcal{P}^*$, that form the APR, the last step is to store this information in memory in a data-structure that allows its efficient use in a wide range of tasks. The optimal

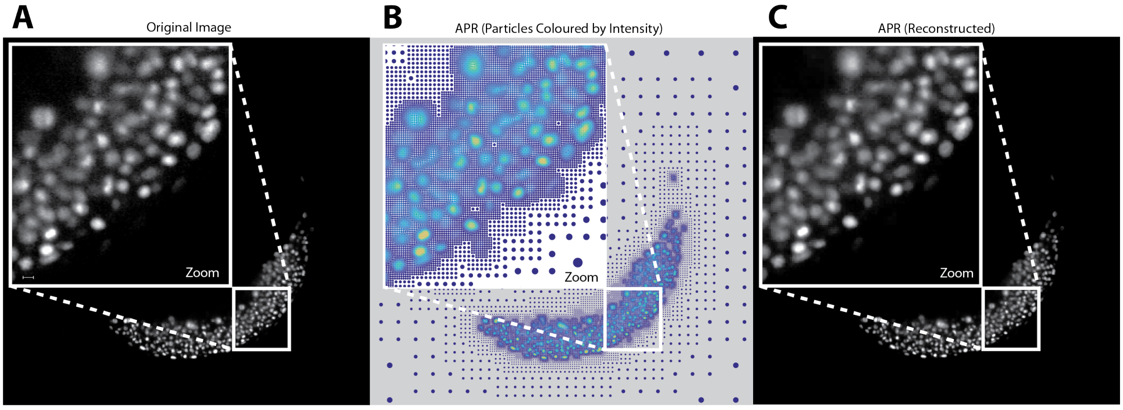

Supplementary Figure 33: A 2D slice showing the original image I (**A**), particles of the APR coloured by intensity (**B**), and reconstructed image $\hat{I}_{pc}$ (**C**) of labelled cell nuclei for a developing Zebrafish (Images courtesy of Gopi Shah, Huisken Lab, MPI-CBG Dataset number: 7 Supplementary Table 3) The insets show a close up of the same region. The particle rendering was created by rendering all particles from Particle Cells from which the image plane intersects.

data-structure will be dependent on the particular use-case, or algorithms, with which the APR is being used. Given the Particle Cells being a subset of a full oct-tree decomposition of the domain, for some tasks, a tree decomposition may be optimal. However, the majority of image processing algorithms have been designed to be implemented over pixel images stored as large contiguous arrays of pixels. This format has the advantage of fast and cache-efficient local neighbor access, and the implicit coding of each pixels spatial coordinates from the pixel data layout, providing performance and memory benefits. Therefore, we have opted to use data-structures that attempt to mimic these efficient properties of pixel images, namely, the implicit coding of spatial and resolution information, and fast neighbor access.

Here, we use the Sparse APR (SA) data structure. It is similar to compressed row storage for sparse matrices, in that only the y coordinate is stored in a sparse manner per level. The SA data structure separates the storage of the spatial coordinates and neighbor access in an Access Data class, from the storage of the particle properties such as intensity stored as a large contiguous array and is summarized in Supplementary Note: 34. The figure is in 2D but directly translates to 3D, where z is treated in the same manner as x. For every non-empty level and x and z spatial index, a red-black tree, using std::map, stores a key-value pair for each contiguous block of Particle Cells in the y spatial index direction. Each value-key pair is organised as follows $\{y_{begin}, \text{global index}, y_{end}\}$, where the key ($y_{begin}$) is the first y Particle Cell spatial index in the block, and the value contains first the global index of the first particle in the block, and the final y Particle Cell spatial index (y_{end}). On average, we find there are twenty times less key-value pairs then Particle Cells in the APR. The structure allows random access by finding the closest key by a tree search, from which the co-ordinate and global index can be directly calculated. The search cost, is worst case $\mathcal{O}(\log(G))$, where G is the number of key-value pairs for the given x, z, $level$ row. Across the Exemplar benchmark datasets, there was on average 7 key-value pairs per row.

Neighbor Access

Neighbours are accessed by utilizing the random access of the SA data structure, and the fact that all neighboring Particle Cells can only differ by one level, and that Particle Cells form a spatial partition. The neighbors in a particular direction are obtained by first querying the same Particle Cell level, then one lower level, and

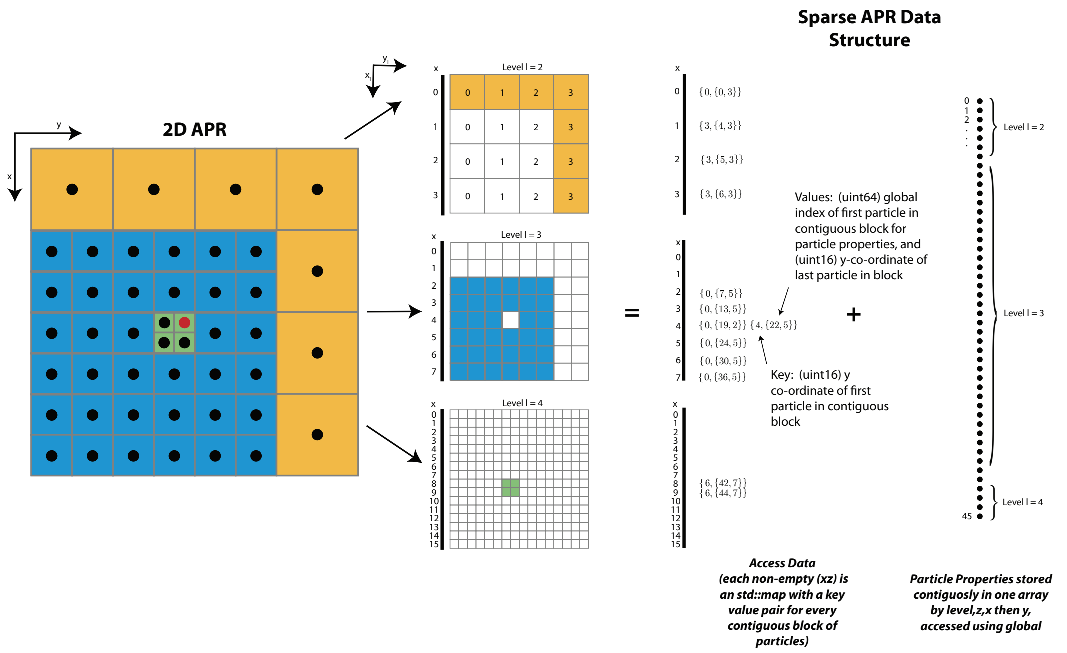

Supplementary Figure 34: Schematic showing the construction of the data structure, using a 2D APR example for illustration. The Sparse APR (SA) data structure contains an access structure, utilizes an std::map data structure that stores a key for every contiguous block of particles in the y direction, and is used for determining the y spatial coordinate of the particle and global index. Each contiguous block is identified by its first y-coordinate as the key, and its global index and final y coordinate. The other spatial coordinates (x, z) and resolution level (l) are implicit in the data layout. Particle properties such as intensity are then stored in a contiguous array.

finally the higher level is searched if the Particle Cell exists. Once the Particle Cell is found, the global index can be directly calculated and therefore all particle properties accessed. The neighbor access is further aided by the storage of the location of the last key-value pair that was accessed in each direction and level. This allows for the searches in the tree only to be needed when a new neighboring contiguous block is required.

Memory Cost

The storage of the SA data structure requires both the Access data and any particle properties. The memory cost of particle properties directly scale with the number of particles, with negligible overhead and therefore reflects the CR of the dataset. The cost of the Access data is less transparent and depends on the original size of the image, the spatial structure of the given APR, and the given compiler and hence the implementation of the red-black tree. The storage cost can be split into the cost of storing the x, z and level of the Particle Cells, and the cost of the std::map and key-value pairs. The first cost scales only with l_{max} and the x and z dimensions, the second is content dependent. Using memory profiling across the Exemplar benchmark datasets we estimated the memory cost, finding that the overhead of the access data has a mean of 8.7 and median of 5.4 bits per particle. Hence, the access data results in a roughly 50% over-head above the cost of storing particle intensities as 16-bit unsigned integers in memory.

**Dataset**	N_x	N_y	N_z	N	**Labelled**	**Specimen**	**Microscope**	**Source**
1	1920	1080	201	4.17E+08	Membrane	Phallusia	Custom LSFM [23] Hufnagel Lab, EMBL	[24] Ulla-Maj Fiuza, Lemaire Lab CRBM (CNRS)
2	3169	1097	181	6.29E+08	Vasculature	Zebrafish (*Danio rerio*)	Custom SPIM [25]	Stephan Daetwyler, Huisken Lab MPI-CBG & Morgridge Institute for Research
3	3083	970	148	4.43E+08	Vasculature	Zebrafish (*Danio rerio*)	Custom SPIM [25]	Stephan Daetwyler, Huisken Lab MPI-CBG & Morgridge Institute for Research
4	1824	834	809	1.23E+09	Nuclei	Fly (*Drosophila melanogaster*)	Zeiss Z.1	Tomancak Lab MPI-CBG
5	532	1352	11	7.77E+06	Nuclei	Fly (*Drosophila melanogaster*)	Custom LSFM [26]	Royer Lab CZ Biohub and Keller Lab Janelia Farm
6	1920	1200	80	1.84E+08	Nuclei	Fly (*Drosophila melanogaster*)	Zeiss Z.1	Tomancak Lab MPI-CBG
7	1094	1162	637	8.10E+08	Nuclei	Zebrafish (*Danio rerio*)	Custom SPIM [27]	Gopi Shah, Huisken Lab MPI-CBG & Morgridge Institute for Research
8	2354	972	39	8.97E+07	Nuclei	Mouse	Confocal	DZNE (Christopher Schmied)
9	1000	1820	975	1.77E+09	Nuclei	Flour Beetle (*Tribolium castaneum*)	Zeiss Z.1	Akanksha Jain, Tomancak Lab MPI-CBG
10	1920	1080	335	6.95E+08	Membrane	Phallusia	Custom LSFM [23] Hufnagel Lab, EMBL	[24] Ulla-Maj Fiuza, Lemaire Lab CRBM (CNRS)
11	1920	1120	178	3.83E+08	Nuclei	Fly (*Drosophila melanogaster*)	Zeiss Z.1	Tomancak Lab MPI-CBG
12	1000	1820	975	1.77E+09	Membrane	Flour Beetle (*Tribolium castaneum*)	Zeiss Z.1	Akanksha Jain, Tomancak Lab MPI-CBG
13	960	960	548	5.05E+08	Vasculature	Zebrafish (*Danio rerio*)	Custom SPIM [25]	Stephan Daetwyler, Huisken Lab MPI-CBG & Morgridge Institute for Research
14	1200	1920	95	2.19E+08	Nuclei	Zebrafish (*Danio rerio*)	Zeiss Z.1	Tomancak Lab MPI-CBG
15	3169	1097	181	6.29E+08	Vasculature	Zebrafish (*Danio rerio*)	Custom SPIM [25]	Stephan Daetwyler, Huisken Lab MPI-CBG & Morgridge Institute for Research
16	1000	1820	975	1.77E+09	Nuclei	Flour Beetle (*Tribolium castaneum*)	Zeiss Z.1	Akanksha Jain, Tomancak Lab MPI-CBG
17	3935	988	219	8.51E+08	Vasculature	Zebrafish (*Danio rerio*)	Custom SPIM [25]	Stephan Daetwyler, Huisken Lab MPI-CBG & Morgridge Institute for Research
18	3736	1432	379	2.03E+09	Nuclei	Mouse	Confocal	DZNE (Christopher Schmied)
19	1000	1820	975	1.77E+09	Membrane	Flour Beetle (*Tribolium castaneum*)	Zeiss Z.1	Akanksha Jain, Tomancak Lab MPI-CBG

Supplementary Table 3: Description and source of the nineteen exemplar benchmark datasets used in the performance validation and processing sections of the main text. Note the MPI-CBG is the Max Planck Institute of Molecular Cell Biology and Genetics, EMBL is the The European Molecular Biology Laboratory, and DZNE the Deutsches Zentrum fr Neurodegenerative Erkrankungen e.V. and CRBM the Centre de Recherches de Biochimie Macromolculaire

Dataset	E	λ	I_{th}	σ_{th}	PSF_w	APR (GB)	Image Size (GB)	CR	APR MCR	APR MCR (WNL q=2)	Pixel MCR (lossless)	Pixel MCR (WNL q=2)	Pipeline time (s)	Pre-processing
1	0.1	3	120	30	2	0.025	0.83	18.5	33.9	93.4	3.5	58.3	1.6	
2	0.1	3	998	100	2	0.024	1.63	39.2	67.7	228	3.4	10.7	3.2	
3	0.1	4	50	60	2	0.016	0.89	25.8	54.0	153	3.1	100.5	1.7	
4	0.1	5	98	200	2	0.207	2.46	8.4	11.9	34.1	2.1	10.8	5.6	Deconvolution
5	0.1	3	40	50	2	0.023	0.16	3.6	6.8	18.6	3.9	16.7	0.4	
6	0.15	3	950	500	2	0.025	0.37	10.4	14.8	39.1	2.0	20.7	0.9	
7	0.125	4	110	50	2	0.078	4.02	27.7	51.6	172	6.8	59.2	8.1	
8	0.1	1	5	5	2	0.001	0.90	206.8	705.2	1505	131	531	1.7	Enhanced
9	0.15	3	2000	1000	2	0.096	3.55	29.7	36.8	98.6	2.0	87.5	6.7	Deconvolution
10	0.1	3	110	200	2	0.021	0.83	22.8	40.1	115.5	3.6	26.7	1.6	
11	0.1	4	400	50	2	0.027	0.77	18.1	28.2	65.8	2.2	39.4	1.4	
12	0.125	3	3000	1500	2	0.059	3.55	48.9	60.1	167	2.0	125.9	6.7	Deconvolution
13	0.1	3	1100	500	2	0.045	1.01	16.4	22.5	55.4	2.1	10.8	1.9	
14	0.1	2	250	80	2	0.036	0.44	6.7	12.0	31.0	2.5	19.1	1.0	
15	0.1	3	998	100	2	0.020	1.26	37.6	63.1	205	3.1	9.5	2.2	
16	0.15	3	300	100	2	0.160	3.55	13.7	22.2	63	2.2	17.3	7.3	Deconvolution
17	0.1	2	1030	200	2	0.053	1.70	21.0	32.3	84	2.5	7.1	3.0	
18	0.15	0.5	10	5	2	0.004	4.06	372.2	1134.5	2347	154	581	7.6	Enhanced
19	0.15	3	800	300	2	0.055	3.55	43.3	64.3	184	2.2	86.6	6.9	Deconvolution

Supplementary Table 4: Summary of parameters used and summary statistics for the nineteen exemplar Benchmark datasets detailed in Supplementary Table 3 and used in the apr validation and processing sections of the main text. Note that for those images where preprocessing was undertaken, this was done prior to the recieving of the image by the source. For details on the lossy compression results and implementation see Supplementary Note: 20 .

Supplementary Note 19: APR formation timing

	Total (s)	Compute $\|\nabla I\|$	Compute σ	Initialize Pyramid	Compute $\mathcal{L}$	Pulling Scheme	Downsample Intensities	APR Data Structure	Data rate (MB/s)
Mean	3.658	58.8%	12.0%	0.4%	4.1%	3.5%	3.5%	4.0%	507.73
Median	2.198	60.6%	12.1%	0.3%	4.1%	3.4%	3.4%	3.5%	523.34
Standard Deviation	2.742	6.7%	2.0%	0.2%	0.5%	0.5%	0.5%	1.7%	45.86

Supplementary Table 5: **Timing summary of APR pipeline on Exemplar benchmark datasets**. Summary of the total time taken, and the percentage of each step in the pipeline, and the estimated data-rate of the pipeline for forming the APR from an image in memory.

Here we provide additional information for the computational cost, or execution time, for forming the APR from an original image with N pixels. The steps of forming the APR have been summarized the schematic in Supplementary Figure 24 above. The pipeline can be broadly grouped into three steps, first calculating the Local Resolution Estime $L(y)$ using filtering operations on the original image, then forming $\mathcal{L}_n$ and finding $\mathcal{V}_n$ using the Pulling Scheme and lastly constructing an APR data structure. Below, we first address the overall cost and then provide more detailed analysis for the Pulling Scheme. We have not included the time taken to load the image in memory in the analysis here.

Full pipeline

The pipeline has worst-case linear complexity in N, as all steps, excluding the Pulling Scheme, are linear complexity in N. Further, the pipeline cost is constant concerning choice of pipeline parameters (λ and PSF_w), with exception of the impact of E on the number of particles. Supplementary Table 5 summarizes the execution time of forming the APR using the Exemplar benchmark datasets. The total pipeline takes on average 3.65 seconds to form the APR data structure from an input image in memory. This translates to an average data rate of 507 MB/s per second on our benchmark machine using 10 OpenMP threads. Across all steps, the computation of the gradient magnitude is most costly taking on average 58.8 % of the pipeline total cost, while the Pulling Scheme only accounts for 3.5 %. The gradient calculation step includes the calculation of the smoothing B-splines, and its relatively high cost is a result of it being the only step performed on the full pixel image. We note that the Pulling Scheme and APR Data Structure steps cost depends on the final number of total particles. Supplementary Table 4 gives the Pulling Scheme and full pipeline time values for each dataset. Further, Supplementary Note: 35, shows the linear scaling of the pipeline, and speed up through shared memory parallelization using OpenMP. From the above values, we claim the implementation here is within the real-time values that have been given in the literature ([28],[29]), hence allowing potential calculation of the APR during acquisition.

Pulling Scheme

We also present additional benchmarks here using only serial execution. We tested the Pulling Scheme for randomly generated $\mathcal{L}$. This allows the ability to directly alter inputs to the Pulling Scheme without having to consider the whole pipeline and how to generate the appropriate synthetic image. In the first plot of Supplementary Figure 36, we show the scaling of the Pulling Scheme for a fixed ratio of $\#\mathcal{L}$ and N. In all benchmarks are run with Particle Cells in $\mathcal{L}$ sampled uniformly and randomly from level l_{min} to l_{max} with a set probability. First, we find confirmation of the worst case linear scaling represented by the Worst-case curve. This benchmark corresponds to the largest $\#\mathcal{L}$ for a given image size N. We also ran three other ratios, .1, .01, and .001, finding linear scaling for all. Each of these benchmarks corresponds to scaling together both the image size and the number of particle cells in $\mathcal{L}$.

In the second benchmark, we fix the image size and increase the number of Particle Cells in $\mathcal{L}$ that are randomly generated. In the second plot of Supplementary Figure 36, we plot the number of seed Particle Cells

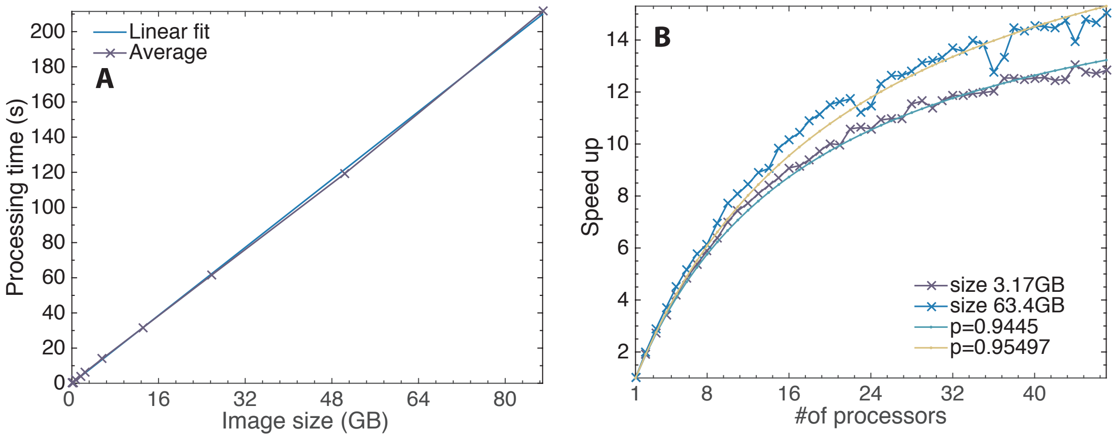

Supplementary Figure 35: **A** shows the linear scaling in N of the total processing time to generate the APR on 12 images ranging from 120x120x120 (4 MB) up to 3600x3600x3600 (88.9 GB) using 20 cores. The results were averaged over 5 repetitions. **B** shows a strong scaling of the pipeline, showing the speed up of the total processing time using an increasing number of CPU cores for fixed images of size 3.17 GB and 63.4 GB. The datasets were generated by tiling Exemplar dataset 17. For both datasets, Amdahl's law was fit (Speed up = $\frac{1}{(1-p)+\frac{p}{n}}$, where n is the number of CPU cores used), with the parallel fraction p reported in the inset legend. The benchmarks were run on a server node with two CPUs with in total 48 Intel(R) Xeon(R) CPU E7-4830 v3 @ 2.10 GHz cores, and 1 TB RAM

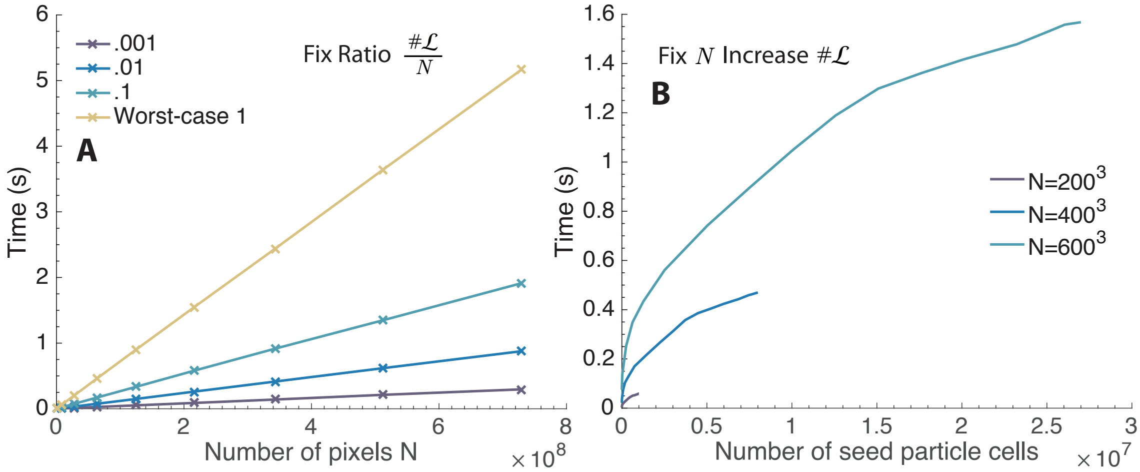

Supplementary Figure 36: **A** shows the Pulling Scheme execution time against image size for four different fixed ratios of $\frac{\#\mathcal{L}}{N}$. The Particle Cells in $\mathcal{L}$ where randomly generated and the results averaged across 10 realizations. All ratios showed linear scaling that was confirmed in a log-log plot. The yellow line corresponds to $\mathcal{L}$ containing all particle cells between l_{min} and l_{max}, representing the worst-case performance. **B** shows the average execution time for three different fixed image sizes N, plotted against the number of seed Particle Cells in $\mathcal{V}$. The number of seed Particle Cells is simply $\#(\mathcal{V} \cap \mathcal{L})$ and hence increases linearly with $\#\mathcal{L}$. The relationship does not represent a polynomial scaling.

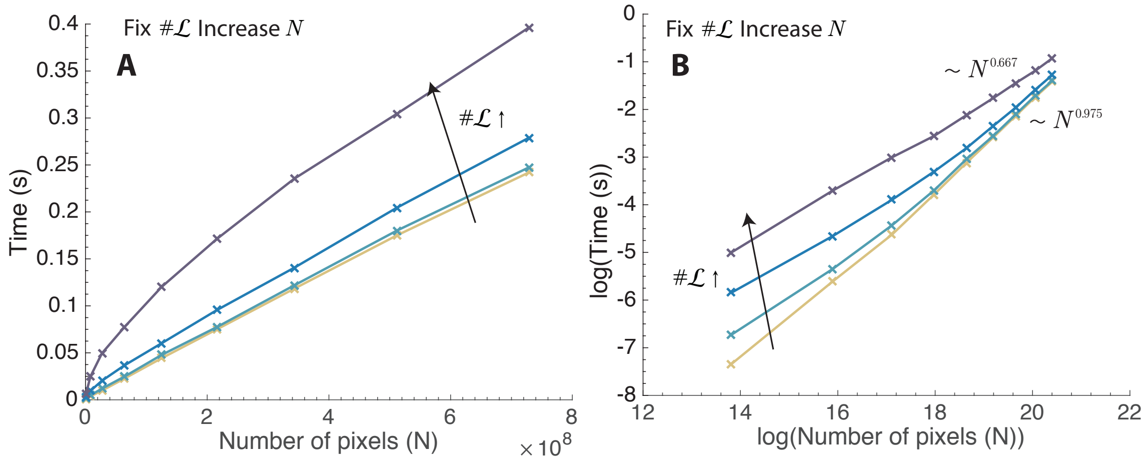

Supplementary Figure 37: **A** shows the average execution time against N four sizes of $\#\mathcal{L}$. The values were set at fixed ratios of the number of Particle Cells in the $N = 100^3$ case. The arrow notes the direction of increasing $\#\mathcal{L}$. The second plot shows the same data on a log-log plot. Linear regression shows sub-linear scaling for all plots for large N. We find that the polynomial scaling coefficient appears to decrease with increasing $\#\mathcal{L}$. The yellow line for the smallest value of $\#\mathcal{L}$ for the highest five N values had a gradient of 0.975 and Rsquare of 1, and the purple curve representing the largest value of $\#\mathcal{L}$ had a gradient of 0.667 and Rsquare again of 1.

in $\mathcal{V}$ for three different image sizes N. The number of seed Particle Cells is the number of Particle Cells given by $\#(\mathcal{L} \cap \mathcal{V})$. We find this is the appropriate variable when compared to $\mathcal{V}$ because the number of neighbor search operations is directly proportional to the number of seed Particle Cells, and not simply the absolute $\#\mathcal{V}$. For all image sizes N we find non-polynomial scaling. With the rate of increase in execution time decreasing as $\#(\mathcal{L} \cap \mathcal{V})$ increases. We note that the same relationship is seen for $\#\mathcal{L}$ (they are proportional), but arguably its is the number of seed cells that is the relevant variable. Empirically the relationship does not seem to be a polynomial nor logarithmic. In the last benchmark, we instead fix the total number of Particle Cells in $\mathcal{L}$ and consider the execution time as the image size N is increased. The results are shown in the first plot of Supplementary Figure 37 for four different numbers of Particle Cells. The four levels were set at ratios of $0.001, 0.01, 0.1$ and 1 of the maximum number of Particle Cells in the $N = 100^3$ image. For all four levels, we find sub-linear scaling in N. That is, the execution time increases at a decreasing rate as N increases. This is confirmed in the second plot, that shows the log-log plot of the same data. Interestingly, the polynomial growth coefficient decreasing as the number of Particle Cells in $\mathcal{L}$ increases. With the smaller number of Particle Cell benchmark being almost linear, scaling at $\sim N^{0.975}$, and the largest number of Particle Cells at $\sim N^{0.667}$.

From the fixed N benchmark we find that the dominant component of the computational cost comes from the number of seed Particle Cells in $\mathcal{V}$. However, the number of seed Particle Cells is constant across N. Therefore, as N increases only the 'cheaper' steps of adding boundary and filler Particle Cells are increased.

In summary, we have confirmed that the Pulling Scheme has worst-case linear scaling in N. Further, the computational cost for fixed N is proportional to the level of information content through the number of seed Particle Cells. However, we have no exact form for this scaling behavior, but it is sublinear. Further, for a fixed size of $\mathcal{L}$ and increased N we find sublinear relationship, with a scaling rate that is inversely proportional to the number of Particle Cells in $\mathcal{L}$.

Supplementary Note 20: File-storage of the APR

We store the APR using the HDF5 file format [9] and BLOSC HDF5 plugin [10] for lossless compression. For BLOSC, the Zstd compression algorithm is used with compression level 2 and shuffling activated. The required information for reconstruction of the SA data structure is stored. The storage of the Access data requires the storage of the key-value pairs and the locations of the non-empty rows. Particle properties are stored in a single contiguous array. Stored in this way the Particle Cell spatial and resolution information is highly compressed. The high compression is reflected in that on average 89% of the bytes are used storing the particle intensities. Further, in the limiting case where the number of particles is equal to the number of input pixels, the particle intensities account for 99.99 % of the storage cost.

Lossy APR Compression

In the basic lossless storage method for the APR, all particle intensities are stored in a lossless manner. In particular, those intensities at pixel resolution (accounting for the majority of particles), are directly sampled from the noisy pixels. Hence, additional lossy compression can be done to reduce the cost of storing these particles. Here we present the results of using the Within Noise Lossless (WNL) compression strategy presented in [30]. This is intended to highlight how the APR can be used to enhance existing compression algorithms both in terms of computational cost and performance.

The WNL method approach was re-implemented in LibAPR, both with and without the neighborhood prediction step. Here we used the approach of only applying the variance stabilization step for the APR as for the results here we found that for the APR this provided the better compression and computational cost trade-off.

Across all benchmarks the some parameters were fixed, namely $e = 1.6$ and conversion factor as $65363/30000$. For all the APR results presented in Table 1 and Supplementary Table 4, the quantization factor $q = 2.0$, and the background value was set automatically as $I_b = 0.9 I_t h$, where $I_t h$ is as set for the APR creation. The resulting quantized symbols are then compressed losslessly using the BLOSC HDF5 plugin for the standard APR. Further, the Particle Cell type is no longer stored in lossy compression benchmarks, to provide a clearer 'one-to-one' comparison with the pixel images, and it is not a requirement for APR processing.

In addition we compare the WNL approach run on APR (no prediction), and also on the original pixel image (no prediction). For the pixel image, the prediction step leads to improved compression performance, and therefore is used in the exemplar benchmarks in Supplementary Table 4. However, for the APR this the prediction step did not provide any noticable performance improvement. Here, for the setting of the background value $I_b = 1000$ to that of the ground truth image. Supplementary Figure 38 shows the PSNR of the image without the background and Memory Compression Ratio (MCR) trade-off for the compressed pixel image and APR for q ranging from 0.1 to 12. The PSNR is computed only in the foreground region, as otherwise the comparison with the APR is biased, as it denoises the background region resulting in a significantly higher PSNR when compared to the original image.

We find from the results of a synthetic sphere image in Supplementary Figure 38 that the behaviour of the PSNR for increasing quantization q is similar across both the APR and Pixel image. However, for a given q the achieved MCR of the APR is on average 3.7 times higher. Further, given the APR approach does not use prediction, the computational cost of the APR-WNL approach will be CR times faster than the comparable pixel-WNL algorithm within prediction as no neighbor access is required. The second pane of the figure then shows representative slices of the compressed and un-compressed images for both the original pixel and APR. We note the similarity in image quality across the Pixel and APR compressed approach, despite the increased MCR.

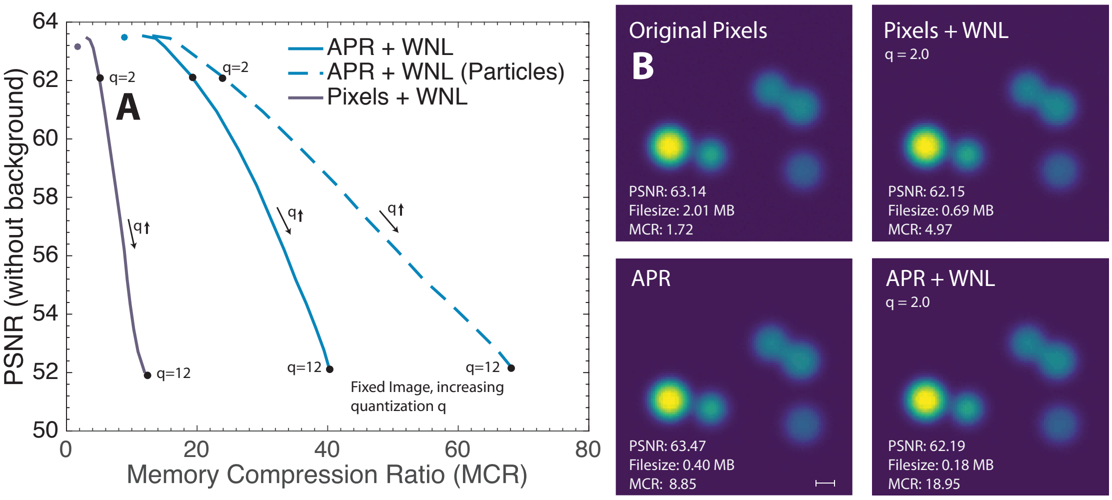

Supplementary Figure 38: **A** compares the results of performing of WNL compression strategy outlined in [30] directly on the original pixel image and on the APR particles for a 120^3 sphere test image corrupted by Poisson noise where the quantization level q is varied from 0.1 to 12. The plot shows the Peak Signal to Noise Ratio (PSNR), of the foreground regions set using the ground truth image, of the uncompressed image in the case of pixels, and piece-wise constant reconstruction for the APR, vs the Memory Compression Ratio (Original File Size/Compressed File Size). The MCR is also shown for the APR when only storing the particle intensities, and not the spatial information in $\mathcal{V}$ (dashed line). The blue dot represents the standard compressed APR, and the purple dot the losslessly compressed original image. **B** provides an examples of the test image at a fixed slice for the original image, pixels compressed with WNL ($q = 2$), the original APR, and the APR compressed with WNL and $q = 2$. The scale bar indicates 10 pixels.

For the exemplar data benchmark datasets the full results are given in Supplementary Table 4 for $q = 2$ on both the original pixel images and the APR. For the pixel image the prediction step was included as this provided an increase in compression ratio. On average the APR provided a 5.06 times higher MCR across the exemplars. For comparison in Supplementary Table 4 the MCR for the image losslessly compressed using the same approach as the APR data is provided.

Supplementary Note 21: APR particle graph

Many processing tasks on images require the formulation of pixel images as a graph, for example, graph-cut methods [31]. Pixels are set as the nodes, and edges are created between adjacent pixels. Although the APR has changing resolution across the domain, a similar symmetric particle graph can be constructed from the APR by using adjacent particle cells, or formally, an integral interaction neighborhood. Supplementary Figure 39, shows such a graph restricting neighbors across the faces of particle cells, analogous to a Von Neumann (or face-connected) neighborhood. In a classical pixel image graph, each node (pixel) would have the same number of neighbors. However, due to the adaptive sampling in the particle graph, the number of neighbors can vary as the resolution adapts. However, the maximum number and the minimum number of neighbors is bounded. The minimum number of neighbors is $2d$, as in the comparable pixel graph, and the maximum $n2^d$ where d is the dimension (However, empirically in 3D benchmark data the average number of neighbors is less than 6.3).

In the face connected particle graph, particle p_0 and p_1 are neighbors if the line integral of the inverse of

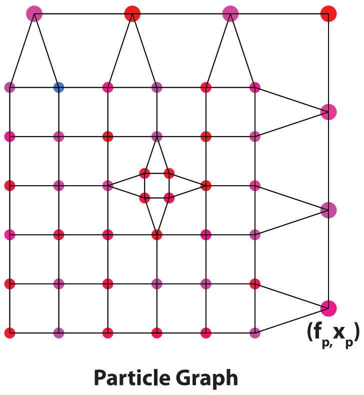

Supplementary Figure 39: The APR paritcle graph, shown in 2D. This aligns with connecting the particles that are in face-connected Particle Cell neighbours of the current Particle Cell.

the Implied Resolution Function is below a certain threshold

$$|(\mathbf{x}_{p_1} - \mathbf{x}_{p_0})| \int_0^1 \frac{1}{R^*(\mathbf{x}_{p_0} + s(\mathbf{x}_{p_1} - \mathbf{x}_{p_0}))} ds \leq \frac{1}{3}\sqrt{9 + d - 1} \tag{204}$$

where d is the dimension. In 1D this bound is 1, and ≈ 1.054 in 2D and ≈ 1.105 in 3D. Particle neighbors in the particle graph can be interpreted as those particle pairs for which the difference between the two value will be approximately $\frac{E}{\sigma(\mathbf{y})}$ where y is a position on the line segment between the points. Note, that these points in dimension greater than 1 exceed the integral neighbor bound by a small factor. If it is wished that the neighbours be guarantee a Reconstruction Condition $\hat{E}$, the APR can be construction with $E = \frac{3\hat{E}}{\sqrt{9+d-1}}$. Due to the isotropic nature of the Implied Resolution Function, this will guarantee that extending the neighborhood, the particles on the edge can be used for any reconstruction, within E.

Supplementary Note 22: Processing benchmark details

All examples are intended as a proof of principle and to indicate performance. Further development would be required to make these algorithms usable to the community, and this is left for future research. An effort was made to optimize both the APR and pixel code in the same fashion to provide reasonable comparisons. The impact of optimization is also motivation for the simple nature of the examples presented here. Shared memory parallelism was used in all steps cases where it was easily achieved using OpenMP [8]. As for the evaluation benchmarks, all performance benchmarks code and analysis data is available on request and are intended to be released open source.

Neighbor access

A core operation in many image-processing algorithms is access to the values of neighboring pixels. For this benchmark, we contrast the time taken to access the values of all face-connected neighbors of each pixel, or

particle. In a pixel image, excluding boundaries, there are always 6 face neighbors. When the pixel image is stored as a contiguous array, accessing these pixels is simply a fixed memory offset for each pixel. However, for the APR, due to the adaptive sampling, the number of face connected neighbors can vary per particles from 6 to 24 (However, in practice, the average number of neighbors per particles in the $CR = 5$ benchmark data sets was 6.23). Neighbour access of face connected neighbors is achieved as described in Supplementary Note: 18.

In this benchmark, for each pixel, and particle, the face-connected neighbor's intensities are averaged and stored in with another pixel image, or APR data-structure of float datatype. For performance, the order in which pixels, or particles, are accessed heavily impacts performance through the impact of the various caches of the processor. Therefore, here we run two benchmarks, one where the pixels, or particles, are iterated over in memory order, and the second where random pixels, or particles, are itearted over with a random ordering. The first case we call the Linear Neighbour Iteration, and the second Random Neighbour Iteration benchmarks. The two examples represent the two extremes of neighbor access in image processing algorithms, with linear iteration on a pixel image corresponding to arguably optimal memory access patterns, and random iteration, the worst case.

For the pixel linear benchmark, the pixels are iterated over in memory direction. For each pixel, the neighbors are looped over, again in memory direction, checking for boundary conditions, accumulating the value in a temporary variable that is then stored in a second array.

For the particle linear benchmark, the particles are iterated over, level by level, in memory direction of the SA data-structure. For both pixels and particles, the results were averaged over 10 consecutive runs.

For the pixel random benchmark, instead of iterating over pixels in memory direction, a pixel is chosen randomly from the dataset, and the neighbors are summed. For the particle random benchmark, similarly, a random particle is chosen, and then the neighbors are summed. In both cases the random pixels and particles are first pre-computed, then iterated over. In both cases, 10000000 random accesses where timed.

For the pixel benchmarks, the memory overhead is the original image and an array of the same size. The data-type of the input images was unsigned 16 bit integers and the output single precision float. Therefore the memory cost $MC = 6N$ Bytes for an image with N pixels. For the particle benchmark the SARA data-structure, with unsigned integer 16 bit intensity plus an additional single precisionfloat particle property was used. The memory cost is as described in Supplementary Note: 18.

Separable pixel filtering

In the pixel separable filter, the 1D filter is convolved successively in each direction. This is done by iterating over the particles in memory direction, checking the boundary conditions, then looping over the neighbor offsets, multiplying by the coefficient and accumulating this in a temporary variable. The temporary variable is then assigned to the output image array.

In the particle separable filter, first, a $2D$ image slice is interpolated using piecewise constant interpolation, with the slice translated such that the filter operation could be done in the contiguous memory direction. Due to the placement of particles at intervals at powers of 2, only the highest resolution aligns with a pixel layer, with other being between the intersection of two layers. All particles that are either aligned with the slice, or intersect, the slice are iterated over, calculating the filter value through accumulating in a temporary variable as for the pixels, and then assigning this to the output particle property. In the case of when the particle intersects between two layers, the output is then the average of the two filter values.

For the benchmarks given here a large filter $1D$ constant filter stencil of length 21 was used. Relative performance results are relatively insensitive to the size of the filter. For the particle filter, each direction was repeated 10 times to get the timing values. However, for each benchmark image, the filter on pixels was only run once. This was due to the higher computational cost restricting a higher amount of repetitions. However, for each CR and N combination, at least 20 independent repetitions were performed.

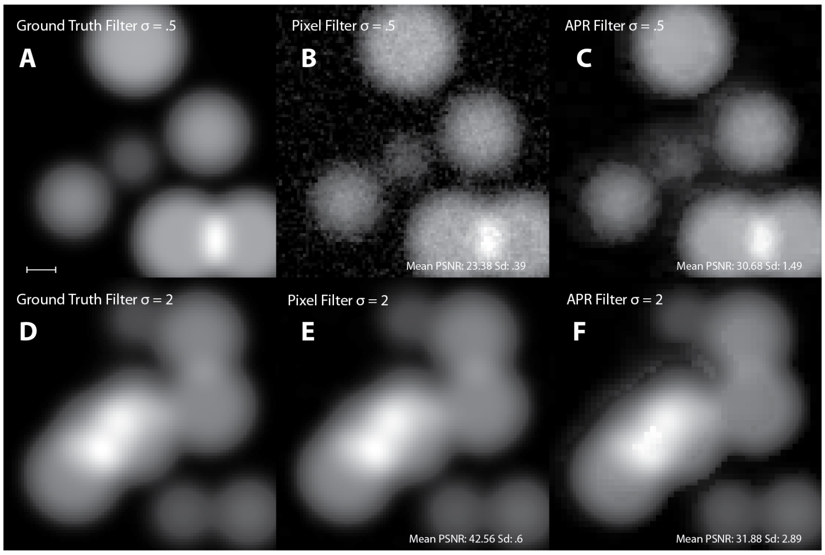

Supplementary Figure 40: Example images of the validation for separable filtering benchmark. The APR and pixel algorithms were run using a narrow Gaussian (**A**-**C**) and broad Gaussian (**D**-**F**), for the pixel filter for noise-free ground truth image (**A** and **D**), the original image (**B** and **E**) and the APR filter the reconstructed to an image (**C** and **F**). The mean and standard deviation of the PSNR compared to the ground truth filter over 100 for a CR5 image of size 250^3 is provided at the bottom of the images. The results show that the APR filter works well for small blur kernels. However, as the kernel gets larger the APR filter no longer produces accurate results, reflected in the low PSNR compared to comparable the pixel filter.

For the pixel benchmarks, the memory overhead is the original image and an output array of the same size. For both the used data-type of the images was float. Therefore the memory cost $MC = N8$ Bytes for an image with N pixels.

For the particle benchmarks, the computation required the SA data-structure, an additional particle property for the output, and an array for the temporary $2D$ image slice used. Therefore the memory cost is $MC = 6N_p + 16\frac{2^{2l_{max}-1}}{3} + 4N^{2/3}$ Bytes. If we ignore the over-head of the SA, and temporary array, the relative memory cost $RMC \approx 0.8 * CR$.

However, the results from these two approaches are different and do not present a fair comparison. To evaluate differences we compared the result of the pixel filter, and the APR filter interpolated to an image, to the ground truth image filtered by the pixel filter for 100 realizations for a fixed image size (250^3, CR5). For comparison, we also computed the result of the pixel filtering on a piecewise constant reconstruction from the APR. We did this for a small $\sigma = 0.5$ and larger $\sigma = 2$ blurred kernel.

Examples of the results are shown in Supplementary Figure 40. For the small blur kernel, the mean APR PSNR was 30.68 with a standard deviation of 1.49, for the original image 23.38 with a standard deviation of 0.395, and for the reconstructed APR 30.71 with standard deviation 1.26. Therefore, the APR filter provided accurate results. However, in the large blur kernel, the mean APR PSNR was 31.883 with a standard deviation of 2.89, for the original image 42.93 with a standard deviation of 0.6, and for the reconstructed APR 42.56 with standard deviation 1.14.

These results indicate that the APR filter no longer produces accurate results. The poor performance can also evident in the **F** in Supplementary Figure 40 where distinct artifacts can be seen. This is the result of the Implied Resolution Function not being valid for the intermediate filtering results in the separable scheme.

In such cases, it seems it would be necessary to result to pixel filtering on a reconstructed APR, as those results are equivalent for larger filters. This also indicates that the failing is not due to the piece-wise constant reconstruction. The poor performance is not a pure function of filter size, but depends on the spatial scales of the resulting function, with large filters designed for edge detection not showing similar issues (not shown).

Hence we conclude, that the above approach is only of specific use, and does not represent a direct replacement for pixel filtering. The example also illustrates the care that must be taken when adapting algorithms from pixels to the APR.

Graph cuts segmentation

For the last performance benchmark, we perform a binary segmentation with graph cuts using an external library. Here, we show how the APR can be used with existing techniques and libraries while still realizing computational and memory benefits due to the reduced number of computational points. For this, we use the maxflow-v3.04 library implementing the min cut-max flow algorithm presented in [31].

Further, we use an energy function that is defined using the information inherent in the APR, as an example of how it could be used. Because of this, then to compare with the same algorithm on the original pixel image, we first compute the energy on the particles and then interpolate them to original images to be then used for the energies for the pixel image.

To use the max-flow algorithm for segmentation, we must define two energy values for each pixel or particle, E_s, giving a likelihood of belonging to the foreground, and E_t, the likelihood of belonging to the background. Additionally, an energy is specified between neighboring pixels or particles in the graph. Here, we use again the face-connected pixel or particle neighborhoods, where we define a symmetric energy between the two neighbors p and p', as $E_{p,p'}$.

As discussed we first define the energy on particles, and then to form the graph for pixel image, we interpolate these energies to a pixel image. For the background and foreground energy, we use the following

$$E_s = 2000|I_p - I_p^{min}| \tag{205}$$

$$E_t = 2000|I_p - I_p^{max}| \tag{206}$$

where I_p^{min} and I_p^{max} are estimates of the local min and max scaled by the resolution of the particle. This is by treating the APR as a tree structure. Maximum and minimum values are propagated up the tree, taking the respective min or max of children values. The value is then averaged over the neighbors at each level in the tree. Then for each particle, the value in the tree k resolutions above is taken as the value for I^{min} or I^{max} respectively. The algorithm essentially creates an adaptive min or max. As the purpose of this benchmark is to focus on the computational and memory characteristics of the algorithm and not propose a new segmentation algorithm or energy, we do not go into further details here.

The edge energy between any two particles, or pixels, is taken as

$$E_{p,p'} = 100\frac{(s_p s_{p'})^2}{81}. \tag{207}$$

As discussed, once the energy has been computed over particles, those were then used to also create a pixel image with the same energies interpolated. Then the appropriate data structures for the max-flow algorithm were generated, the max-flow algorithm run, and the binary labeling of background or foreground extracted from the result.

In the benchmark performance analysis, we assess only the computational time, and memory cost, of the max-flow algorithm, and not the including the generation of the energy or setting up the graph.

Due to the use of an external library, we estimated the memory cost by performing memory analysis on the code with different particle and pixel sizes and empirically evaluating the value. For the performing max-flow on the pixel graph, we found the memory cost $MC = 411.6N$ and for the particle graph $MC = 436N_p$.

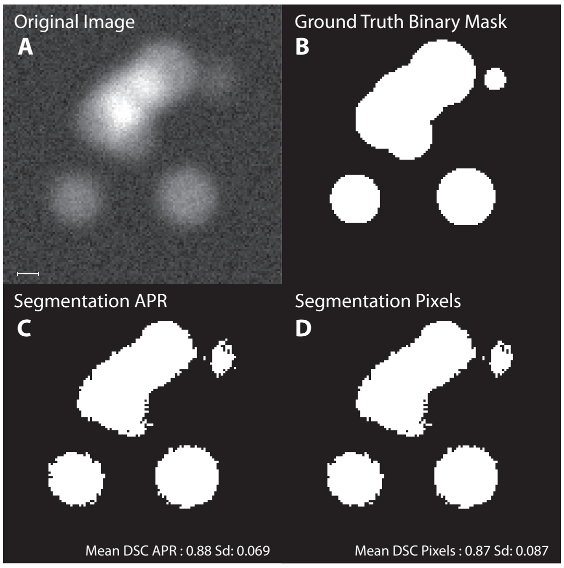

Supplementary Figure 41: Validation for segmentation performance benchmark. The same energy function was used for both pixel **C** and APR segmentation **D**. The ground truth binary image **A** is creating using the binarization of the Object function (Supplementary Note: 15). The APR and pixel segmentation give near identical results as shown by the computed Dice Similarity Coefficients (DSC [32]) mean and standard deviation over 100 repetitions for CR5 images of width 250 given in the figure. The scale bar indicates 10 pixels.

Providing very similar results, this is although the particles can have up to 24 neighbors, on average for the benchmark data sets there is only approximately 6.3.

To validate that the APR segmentation, we compared the APR and pixel image segmentations to ground-truth and calculated the Dice Similarity Coefficient (DSC) [32] for 100 repetitions of a CR5 image of width 250. We define the DSC in our case as,

$$DSC = \frac{\#(S_{gt} \cap S_p)}{\#S_{gt} + \#S_p}, \tag{208}$$

where S_{gt} is the set of pixels in the support of the Object function and S_p, is the estimated set of pixels from the segmentation algorithm, and $\#$ indicates the size or cardinality, of a set. To allow direct comparison with the pixel result, for calculation of the DSC a piecewise constant reconstruction from the APR segmentation was used. Figure 41, shows an example segmentation, with the original image, ground-truth segmentation, and segmentations results for the APR and pixel algorithm given. The calculated DSCs of both approaches are statistically identical, with the APR having a mean of 0.88 and a standard deviation of 0.069 and the pixel segmentation a mean of 0.87 and a standard deviation of 0.087. The closeness of the results can be seen in a visual comparison, with only isolated pixels being different. From above, we conclude that the results of the two approaches are comparable.

For applications to exemplar data, we developed a slightly altered energy function. The background and foreground energy were altered by using one iteration of APR adaptive smoothing (See Section Below), on both the intensity and adaptive min and max. The edge energy between particles was changed to be asymmetric to the following

$$E_{p \to p'} = 100 \exp \frac{I_p - I_{p'}}{d(p,p')(I_p^{max} - I_p^{min})}, \tag{209}$$

where $d(p, p')$ is the distance between the two particles. We found that this energy appeared to give reasonable results across a wide range of the exemplar data-sets with no adjustment of parameters except an intensity threshold for removal of background objects. Hinting that the information gained in the APR allows for regularization of the problem that may help with designing future algorithms with stable parameters across a range of problems.

Adaptive APR Filters

Although smoothing and gradient operations are not well suited to the separable filtering approach shown earlier, a more natural approach for the APR is to define filters not over pixels as for traditional filtering, but over particles. With the filter coefficients acting on the particle neighbors. Using particle neighbors results in the filter adapting its neighborhood size across the domain to the resolution set by the Implied Resolution Function R^* of the APR.

As a first example, we show benchmark results for an adaptive smoothing filter. As for the classic separable filters, each direction is filtered separately with a $1D$ filter $\{0.1, 0.8, 0.1\}$, and in succession with a 1D filter. In the case where the neighbor is of higher resolution, an average of the neighbor particles is used.

Adaptive APR filters are the APR equivalent of the separable pixel filters benchmarked above. Defining the filter over neighboring particles from the particle graph, instead of equally spaced pixels. As the distance between neighboring particles varies across the image, computing an adaptive APR filter is analogous to a spatially adaptive pixel filter with filter size changing to the content of the image.

We also tested an adaptive APR smoothing filter that involves taking a weighted average over neighboring particles. Multiple passes were made for greater smoothing. Comparative results for the same data as the gradient example are shown in Supplementary Figure 42. The adaptive APR smoothing filter showed a higher PSNR (37.8) increase than any fixed kernel Gaussian smoothing on the original image (maximum 33.42). This held for from one to seven passes. We have shown the results for four taps, as that was the best result for the adaptive APR smoothing filter. Therefore the adaptive APR smoothing filter provides an alternative for smoothing other than using a pixel filter approach discussed above.

As a second example we define adaptive gradient filters, similar to standard finite differences, with the coefficients adjusted for the distance between the particles. The gradient filter used here is $\{-\frac{1}{2h_-}, \frac{1}{2h_-} - \frac{1}{2h_+}, \frac{1}{2h_+}\}$, where h_+ and h_- are the distances between particles in the positive and negative directions respectively. In the case where a neighbor is of higher resolution, an average of the neighbor particles is used.

The gradient in each direction is calculated by taking the average of one-sided differences between neighboring particles in each direction (as a particle can have up to 8 neighbors in one direction, i.e. x,y, or z). Supplementary Figure 43, shows the results for benchmark data, where we compare the result with the gradient magnitude calculated using central finite differences on the original and ground truth images for 100 CR5 images of width 250. The adaptive APR filter had an average PSNR of 34.6 and standard deviation of 1.2394 while the average PSNR for the original image approach had an average PSNR of 16.78 with a standard deviation of 0.1802. Hence, the adaptive APR filter shows significantly more robustness to noise for our benchmark data. The algorithm also provides nice denoising properties for the exemplar datasets, as shown in Supplementary Figure 44 for one slice of an LSFM dataset.

APR visualization

A key processing task using LSFM data is visualization. Both for display of the original image data and any processed results. Visualization is a processing task, as the raw data can not be viewed directly, and must be processed 'in some way' to provide a visual representation. For 3D visualization, this is evident, as the image data represents an opaque 3D cube of integers. However, even for visualization of the original 2D image slices usually requires calculation, or manual setting, of a visual contrast range.

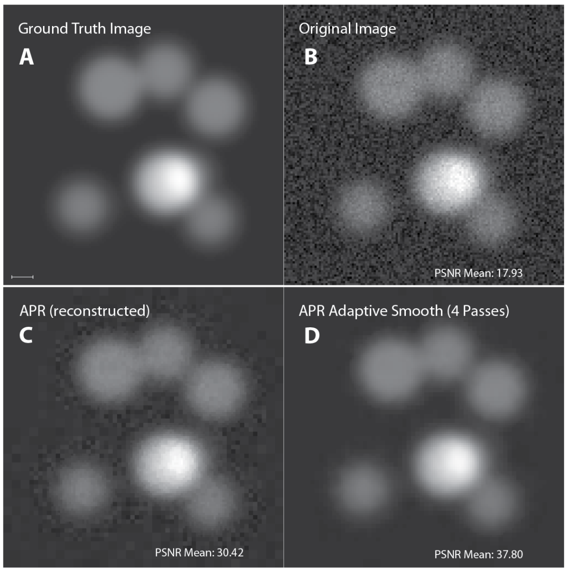

Supplementary Figure 42: Evaluation of APR Adaptive Smooth filter with synthetic data, obtained by filtering in each direction with $\{0.1, 0.8, 0.1\}$ directly with particle neighbors generating an adaptive filter, with multiple particles on one face being averaged. **A**, shows the original ground truth image, **B** shows the original image, **C** APR reconstructed image, and **D** shows an example result of four passes with the filter in each direction. For each image, the mean PSNR with reference to the ground truth image is shown over 200 repetitions for CR5 images of width 250. Four passes achieved the maximum PSNR for the APR. However, any number of passes of those tested (up to 7) exceeded the PSNR of any Gaussian filter on the original or reconstructed image (maximum PSNR of 34.4). The scale bar indicates 10 pixels.

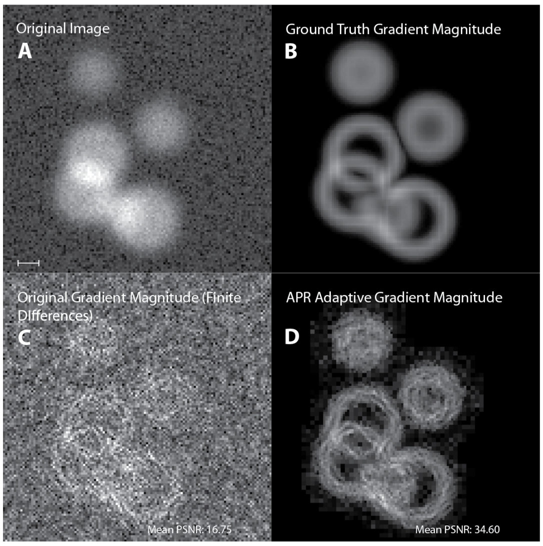

Supplementary Figure 43: Evaluation of Adaptive APR filter used to compute the gradient magnitude with synthetic data, obtained by filtering in each direction with $\{-\frac{1}{2h_-}, \frac{1}{2h_-} - \frac{1}{2h_+}, \frac{1}{2h_+}\}$, where h_+ and h_- are the particle spacings in the positive and negative direction respectively, and multiple particles on one face being averaged. **A** shows the original noisy image, **B** the ground truth gradient magnitude using finite differences, **C** the original image gradient magnitude using finite differences, and **D** APR Adaptive gradient magnitude. The mean PSNR with respect to the ground truth gradient magnitude is given averaged over 100 repetitions, for CR5 $N = 250^3$ images. The scale bar indicates 10 pixels.

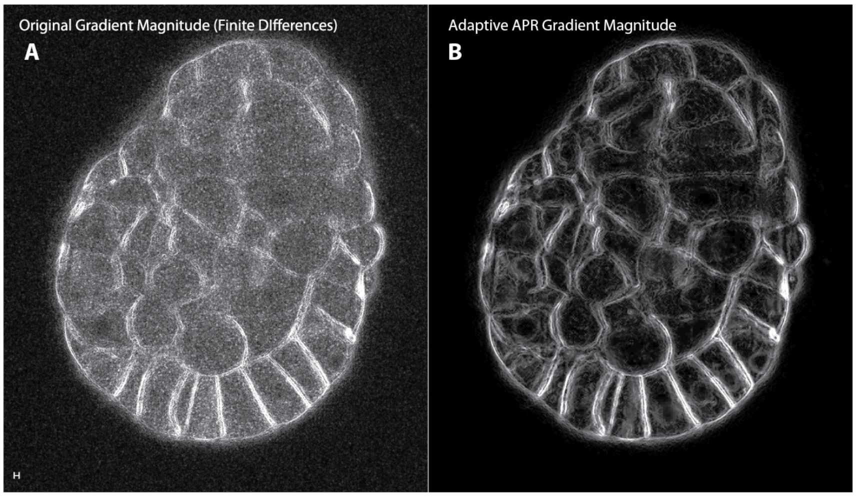

Supplementary Figure 44: Evaluation of Adaptive APR filter used to compute the gradient magnitude on an exemplar dataset 10 in Supplementary Table 3. Image **A** was generated using central finite differences to compute the gradient magnitude on the original image. Image **B** was obtained by using adaptive APR filtering in each direction with $\{-\frac{1}{2h_-}, \frac{1}{2h_-} - \frac{1}{2h_+}, \frac{1}{2h_+}\}$, where h_+ and h_- are the particle spacings in the positive and negative direction respectively, and multiple particles on one face being averaged, and then the image formed using piecewise constant reconstruction. The scale bar indicates 10 pixels.

The APR, rather than restricting the visualization possibilities, extends them when compared to the original image data. Given, as that a pixel image representation can be constructed from the APR, this is not surprising. Here, we discuss three different avenues of APR visualization that can be achieved without returning to the full pixel image. We do not benchmark the relative computational or memory performance below, showing the results as proof of principle. We leave the development of efficient implementations and studies of relative performance to future work.

The first visualization method we present, is visualization by reconstruction, on a slice by slice basis. If we only wish to view one slice at a time, the reconstruction can be done on a slice by slice basis. Hence, this does not require having to reconstruct the whole image (as used in the pixel filtering above). In addition to the multiple examples throughout the thesis above, Figure 6A gives examples of the APR reconstruction and comparison to the input image. In practice, we find that the piecewise constant reconstruction has been sufficient for visualization purposes. This could easily be implemented in real time per slice, as on a 2013 laptop, reconstructing a 1000×1000 slice took approximately .002 seconds. Although, given correct setting of the contrast control, such piecewise constant representations do show significant 'artifacts' as shown in Supplementary Figure 28. If these are not desired more smooth reconstructions could be used as described in Supplementary Note: 10 at an additional computational cost.

Memory cost for $2D$ slice reconstruction is the memory cost of the Sparse APR data structure (SA) plus the cost of storing the $2D$ slice. In comparison standard viewing of a standard pixel image in software such as Fiji [33] requires the storage of the full image in memory.

A perspective ray-cast allows for the visualizing $3D$ content by constructing a $2D$ image by simulating rays that would be seen by an observer from a particular location. However, because an image volume is just intensity values, an algorithm must be specified for turning the intensities seen by each ray into an observed value. The most common algorithm is simply to take the maximum value along the ray, the basis of the maximum projection. This technique is used in current state of the art visualization software such as [34]. Here we have implemented a maximum intensity perspective ray-cast algorithm for the APR. For comparison, we also implemented the comparable algorithm for a pixel image.

Following we describe the principle of the pixel algorithm and then use this as a reference for the description of the APR algorithm. The pixel algorithm involved rastering over each pixel, then calculating which ray this pixel would intersect with, and then updating this ray with the value if it is greater than its current value. This is in contrast to the alternative approach where the image volume is traversed individually for each ray. Each ray corresponds then to a pixel in the final viewed image.

For the APR algorithm, the main difference is that we assign each particle to a ray corresponding to its level l, effectively creating an image view at each resolution level. Once all particles have been traversed, the maximum operation is then propagated between levels from lower resolutions to the highest resolution. Resulting in a final highest resolution image that is viewed. We find that the APR algorithm has moderate overhead, with a PP ratio of approximately 0.75, when compared to the pixel algorithm.

The algorithms compute different results, however as discussed in the main text, they produce in most cases perceptively identical results in normal contrast ranges.

Supplementary Figure 45, shows an example of a maximum perspective ray-cast computed on the original image, and direct on the APR for an LSFM data set. Shown at the given contrast levels the two are virtually indistinguishable. However, there are still distinct differences, with Supplementary Figure 46 highlighting them using a different contrast range. The APR ray cast is done by casting multi-resolution rays through the image, level by level, and then combining the results in a final step. The algorithm has a computational and memory complexity that is $\mathcal{O}(N_p)$, only requiring the SA data structure. Such an algorithm could form the basis of useful visualization software, as currently in for the largest images (approximately $> 1000^3$) can not be visualized at the full resolution in the current state of the art software [34] due to memory constraints.

When computing the ray-cast on a pixel image, the memory required is that of the original image and the ray-cast result. This memory cost is similar to that of the APR ray-cast. The APR ray-cast requires the

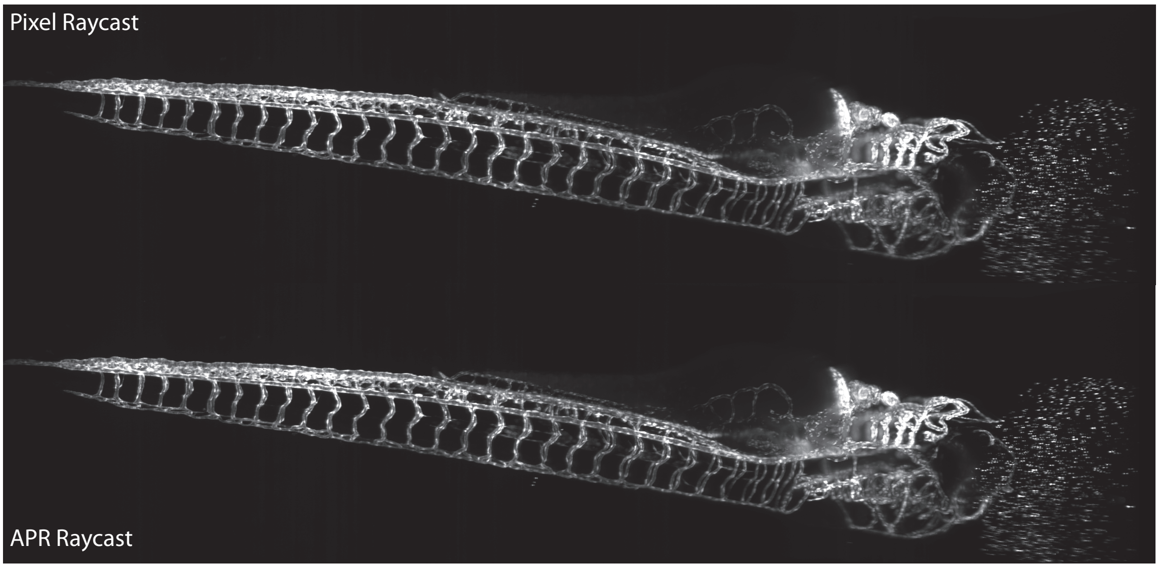

Supplementary Figure 45: Comparison for the same view perspective of an APR and Pixel maximum intensity ray cast of exemplar dataset 17 in Supplementary Table 3.

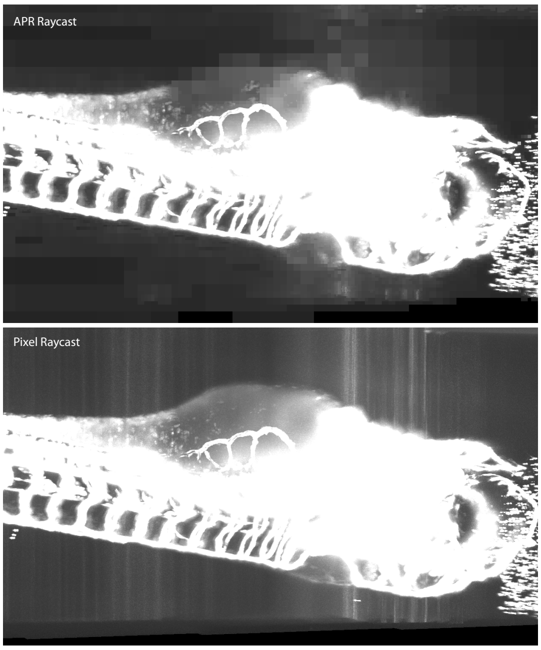

Supplementary Figure 46: Reproduction of the pixel and APR ray-cast example for the same view shown in Figure 45. Contrast has been adjusted to highlight the differences and loss of information for the APR ray-cast resulting from an intensity threshold. (Dataset number: 17 in Supplementary Table 3)

Sparse APR data-structure, and the ray-cast result, plus the down-sampled by two results. For reasonably sized data-sets in both cases the memory cost is dominated by the original image, and APR data-structure respectively. Therefore, the memory reduction for a particular dataset will be approximately $\frac{CR}{1.5}$ (reflecting the cost of storing the access data).

The last visualization method involves direct visualization of the APR. We have given various examples of this in 1D and 2D throughout the thesis above. Not only can the intensity be visualized, but also the particle cell level, location, and type. In the main text, Figure 5 shows an example of this for a small portion of LSFM data in 2D. With the use of thresholding or variable opacity, particles can also be directly rendered in 3D.

Ideally direct renderings memory overhead should only reflect the cost of the SA data-structure, however memory efficient algorithms for rendering have yet to be developed and depend on a GPU implementation and are thus left for future work. The current visualizations, are relatively memory inefficient, requiring the direct storage of the spatial coordinates as floats, in addition to the particle information.

Supplementary References

[1] Cheeseman, B. *The Adaptive Particle Representation (APR) for Simple and Efficient Adaptive Resolution Processing, Storage and Simulations*. Ph.D. thesis, Technische Universität Dresden (2017). URL http://www.qucosa.de/fileadmin/data/qucosa/documents/23424/Dissertation_Cheeseman.pdf.

[2] Saint Raymond, X. *Elementary introduction to the theory of pseudodifferential operators*, vol. 3 (CRC Press, 1991).

[3] Awile, O., Büyükkeçeci, F., Reboux, S. & Sbalzarini, I. F. Fast neighbor lists for adaptive-resolution particle simulations. *Computer Physics Communications* **183**, 1073–1081 (2012).

[4] Okikiolu, K. Characterization of subsets of rectifiable curves in rn. *Journal of the London Mathematical Society* **2**, 336–348 (1992).

[5] Donoho, D. L. & Johnstone, I. M. Ideal spatial adaptation by wavelet shrinkage. *biometrika* 425–455 (1994).

[6] Schrader, B., Reboux, S. & Sbalzarini, I. F. Discretization correction of general integral pse operators for particle methods. *Journal of Computational Physics* **229**, 4159–4182 (2010).

[7] DeVore, R. A., Jawerth, B. & Lucier, B. J. Image compression through wavelet transform coding. *IEEE Transactions on information theory* **38**, 719–746 (1992).

[8] Board, O. A. R. (2013). URL http://www.openmp.org/wp-content/uploads/OpenMP4.0.0.pdf.

[9] The HDF Group. Hierarchical Data Format, version 5 (1997-2017). Http://www.hdfgroup.org/HDF5/.

[10] Alted, F. Blosc, an extremely fast, multi-threaded, meta-compressor library (2017). URL https://github.com/Blosc/hdf5-blosc.

[11] Unser, M., Aldroubi, A. & Eden, M. B-spline signal processing. ii. efficiency design and applications. *IEEE transactions on signal processing* **41**, 834–848 (1993).

[12] Sage, D. Local normalization. http://bigwww.epfl.ch/sage/soft/localnormalization/ (2002 (accessed December 2013)).

[13] Crow, F. C. Summed-area tables for texture mapping. *ACM SIGGRAPH computer graphics* **18**, 207–212 (1984).

[14] Vicidomini, G. Image formation in fluorescence microscopy. In *From Cells to Proteins: Imaging Nature across Dimensions*, 371–393 (Springer, 2005).

[15] Mathews, J. & Walker, R. L. *Mathematical methods of physics*, vol. 501 (WA Benjamin New York, 1970).

[16] Van Vliet, L. J., Boddeke, F. R., Sudar, D. & Young, I. T. Image detectors for digital image microscopy. *Digital Image Analysis of Microbes: Imaging, Morphometry, Fluorometry, and Motility Techniques and Applications* 37–63 (1998).

[17] ArrayFire. Arrayfire library (2015). URL `http://arrayfire.com/`.

[18] Nooruddin, F. S. & Turk, G. Simplification and repair of polygonal models using volumetric techniques. *IEEE Transactions on Visualization and Computer Graphics* **9**, 191–205 (2003).

[19] Min, P. binvox, 3d mesh voxelizer (2017). URL `http://www.patrickmin.com/binvox/`.

[20] Repository of static 3d-meshes. URL `http://193.48.251.101:8080/3dsegbenchmark/dataset.html`.

[21] Schmidt, M. & Lipson, H. Eureqa. *Nutonian, Somerville, Mass, USA* (2013).

[22] Goodfellow, I., Bengio, Y. & Courville, A. *Deep learning* (MIT press, 2016).

[23] Krzic, U., Gunther, S., Saunders, T. E., Streichan, S. J. & Hufnagel, L. Multiview light-sheet microscope for rapid in toto imaging. *Nature methods* **9**, 730–733 (2012).

[24] Guignard, L. *et al.* Contact-dependent cell communications drive morphological invariance during ascidian embryogenesis. *bioRxiv* 238741 (2017).

[25] Daetwyler, S. & Huisken, J. Fast fluorescence microscopy with light sheets. *The Biological Bulletin* **231**, 14–25 (2016).

[26] Royer, L. A. *et al.* Adaptive light-sheet microscopy for long-term, high-resolution imaging in living organisms. *Nature biotechnology* **34**, 1267–1278 (2016).

[27] Schmid, B. *et al.* High-speed panoramic light-sheet microscopy reveals global endodermal cell dynamics. *Nature communications* **4** (2013).

[28] Schmid, B. & Huisken, J. Real-time multi-view deconvolution. *Bioinformatics* **31**, 3398–3400 (2015).

[29] Afshar, Y. & Sbalzarini, I. F. A parallel distributed-memory particle method enables acquisition-rate segmentation of large fluorescence microscopy images. *PloS one* **11**, e0152528 (2016).

[30] Balazs, B., Deschamps, J., Albert, M., Ries, J. & Hufnagel, L. A real-time compression library for microscopy images. *bioRxiv* (2017).

[31] Boykov, Y. & Kolmogorov, V. An experimental comparison of min-cut/max-flow algorithms for energy minimization in vision. *IEEE transactions on pattern analysis and machine intelligence* **26**, 1124–1137 (2004).

[32] Dice, L. R. Measures of the amount of ecologic association between species. *Ecology* **26**, 297–302 (1945).

[33] Schindelin, J. *et al.* Fiji: an open-source platform for biological-image analysis. *Nature methods* **9**, 676–682 (2012).

[34] Royer, L. A. *et al.* Clearvolume: open-source live 3d visualization for light-sheet microscopy. *Nature methods* **12**, 480–481 (2015).
