# Supplementary material for: Adaptive particle representation of fluorescence microscopy images
Source: Nat Commun. 2018 Dec 4;9:5160. doi: 10.1038/s41467-018-07390-9 (PMC6279843; doi:10.1038/s41467-018-07390-9)
Supplement: Supplementary file 3 — Description of Additional Supplementary Files [file 41467_2018_7390_MOESM3_ESM.pdf]

Description of Additional Supplementary Information

Supplementary Video 1: Particle Cell level and Piece-wise Constant Reconstruction: Visualization of the adaptation of the APR by Particle Cell level and piece-wise constant APR reconstruction compared with the original image for exemplar dataset 1 from Supplementary Table 3.

Supplementary Video 2: Graph-Cut Segmentation Example: Maximum projection ray-cast visualization and direct particle rendering of segmentation result for exemplar dataset 13 from Supplementary Table 3. Visualization done using the software Scenery.

Supplementary Video 3: Maximum Projection Ray-cast Comparison: Comparison of maximum projection ray-casts of the APR and of the original pixel image for exemplar dataset 2 from Supplementary Table 3.

Supplementary Video 4: Visualization by Direct Particle Rendering, Example 1: Example of visualization using direct particle rendering with the visualization software Scenery. Image curtesy of Royer Lab (CZ Biohub) and Keller Lab (HHMI Janelia).

Supplementary Video 5: Visualization by Direct Particle Rendering, Example 2: Example of visualization using direct particle rendering with the visualization software Scenery for exemplar dataset 17 from Supplementary Table 3.
